# Supplementary material for: Portimine A toxin causes skin inflammation through ZAKα-dependent NLRP1 inflammasome activation
Source: EMBO Mol Med. 2025 Feb 13;17(3):535–62. doi: 10.1038/s44321-025-00197-4 (PMC11903881; doi:10.1038/s44321-025-00197-4)
Supplement: Supplementary file 2 — Source Data checklist [file 44321_2025_197_MOESM2_ESM.pdf]

I have carefully looked at your figures and prepared a detailed list of the figure panels ("Source data checklist") for which we ask source data – see below.

Beside the panel number I note down some keywords such as 'Numerical Data' or 'Image Data' to classify the experiment and data type. This is to help you and our team to assemble and check the respective files.

I would like you to fill out the SourceData checklist and return it when you submit the revised manuscript and provide the requested source data files along with your resubmission.

In case you are not able to provide source data for one or more of the suggested panels, leave a comment in the blue box at the bottom of the page. You are free to upload any file/image to an external repository (for example BioImage or EMPIAR, see also the attached FAQs document) instead of uploading files to the submission platform. If you decide to do so, please leave a comment to indicate the respective accession number(s) in the blue box at the end of the page: please note that the same accession numbers must also be listed in the "data availability" section of your manuscript.

If during the revision process the panel numbering changes, update the panel number in the respective textbox in the column "New Panel #".

If you have generated new panels for which you can provide source data, please add a comment in the blue box at the end of the page and add the source data files in the corresponding figure folder as indicated in the attached FAQs list file.

When you submit the revised version, upload this document as a "Related Manuscript" file. (Please do not change the file name). Further description on how to upload the source data is provided in the attached FAQs.

You can find more information about SourceData at EMBO Press via this link https://www.embopress.org/pb-assets/embo-site/Guide%20for%20SourceData%20Submission-1656066810500.pdf.

Best,
Hannah

--
Hannah Sonntag, PhD
SourceData Scientific Coordinator,
EMBO https://sourcedata.embo.org

EMBOpress

SOURCEDATA Making data discoverable

SourceData Checklist

Manuscript ID: EMM-2024-19944

SD provided	New Panel #	Panel #	
✓	1B	1B	Image data: whole mount
✓	1C	1C	(LC-)MS: consider external repository (PRIDE)
✓	1D	1D	Image data: Blot
✓	1E	1E	Numerical Data
✓	1F	1F	Numerical Data
✓	2A	1G	Image and numerical data: Micr.image+quantif.
✓	2B	1H	Image and numerical data: Blot+quantif.
✓	3A	2A	Image data: Blot
	EV3[illegible]	2B	Image data: Blot
	EV3[illegible]	2C	Image data: Blot
✓	3B	2D	Image data: Blot
✓	3D	2E	Image data: Blot
✓	3D	2F	Numerical Data
✓	3C	2G	Image data: Blot
✓	3E	2H	Image and numerical data: Micr.image+quantif.
✓	3F	2I	Image data: Blot
✓	3G	2J	Image and numerical data: Micr.image+quantif.
✓	4A	3A	Image data: Blot
✓	4B	3B	Image and numerical data: Micr.image+quantif.

SourceData Checklist

Manuscript ID: EMM-2024-19944

SD provided	New Panel #	Panel #	
✓	4C	3C	Numerical data: Concentration measurement
✓	4D	3D	Numerical data: Kaplan Meier/survival curve
		3E	Image and numerical data: Micr.image+quantif.
✓	4E	3F	Image and numerical data: Micr.image+quantif.
✓	4F	3G	Image and numerical data: Micr.image+quantif.

In this version, the figures for which we do no provide raw results have been either replaced with new data from reviewer requests or transfered to EV figs because of new formatting of the article. Former Figs 2B and C are now in EV3 and former fig 3E has been replaced with EV4B. new figs with raw datas: 2C, 2D, 2E, 3C, 3D, 3E, 4G (all included in source data file)
