# Supplementary figures and images for: Portimine A toxin causes skin inflammation through ZAKα-dependent NLRP1 inflammasome activation
Source: EMBO Mol Med. 2025 Feb 13;17(3):535–62. doi: 10.1038/s44321-025-00197-4 (PMC11903881; doi:10.1038/s44321-025-00197-4)

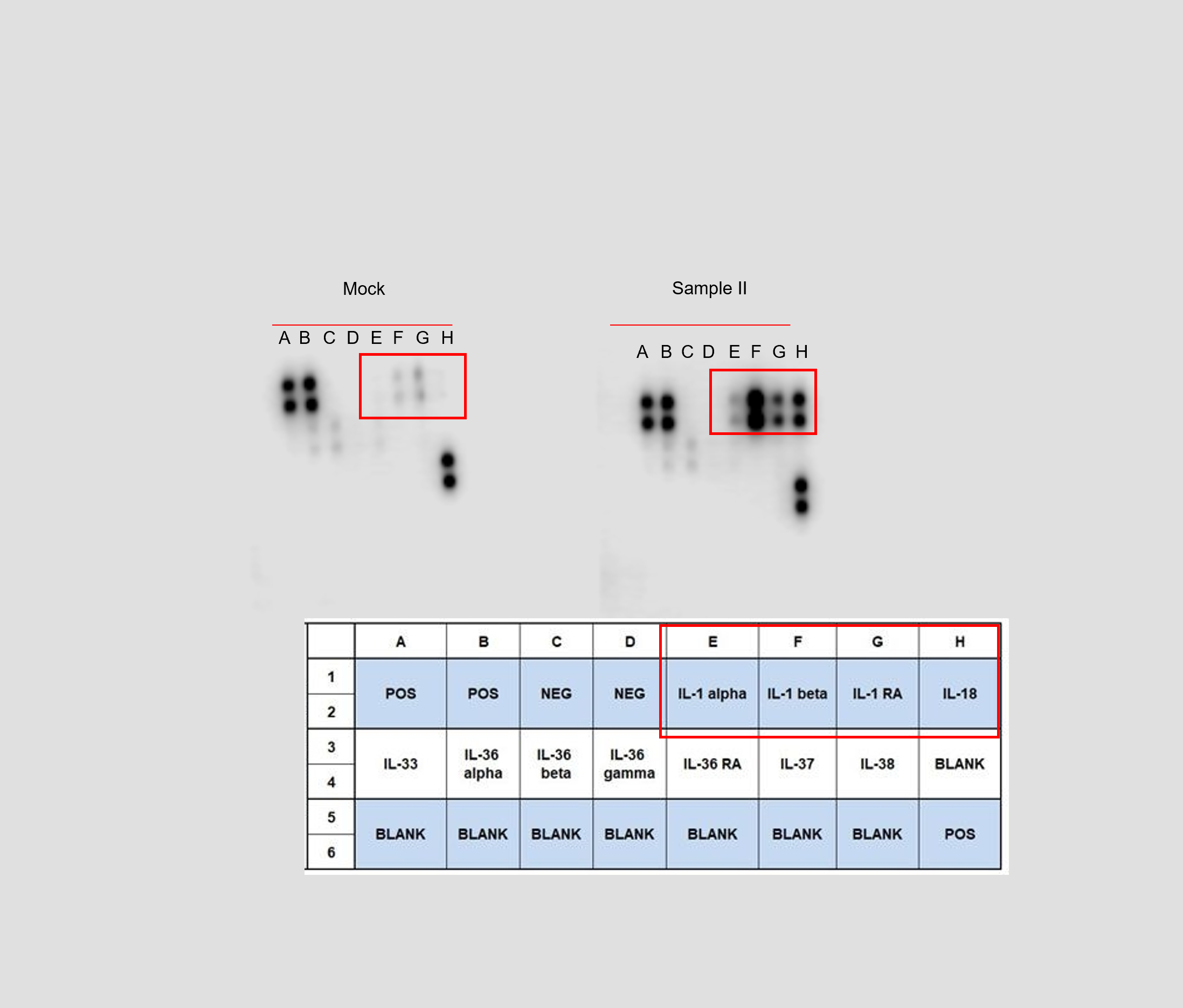

Supplement: Supplementary file 3 — Source data Fig. 1 [file 44321_2025_197_MOESM3_ESM.zip › Figure 1/Fig 1D/Fig 1D IL-1 cytokine array.tif]

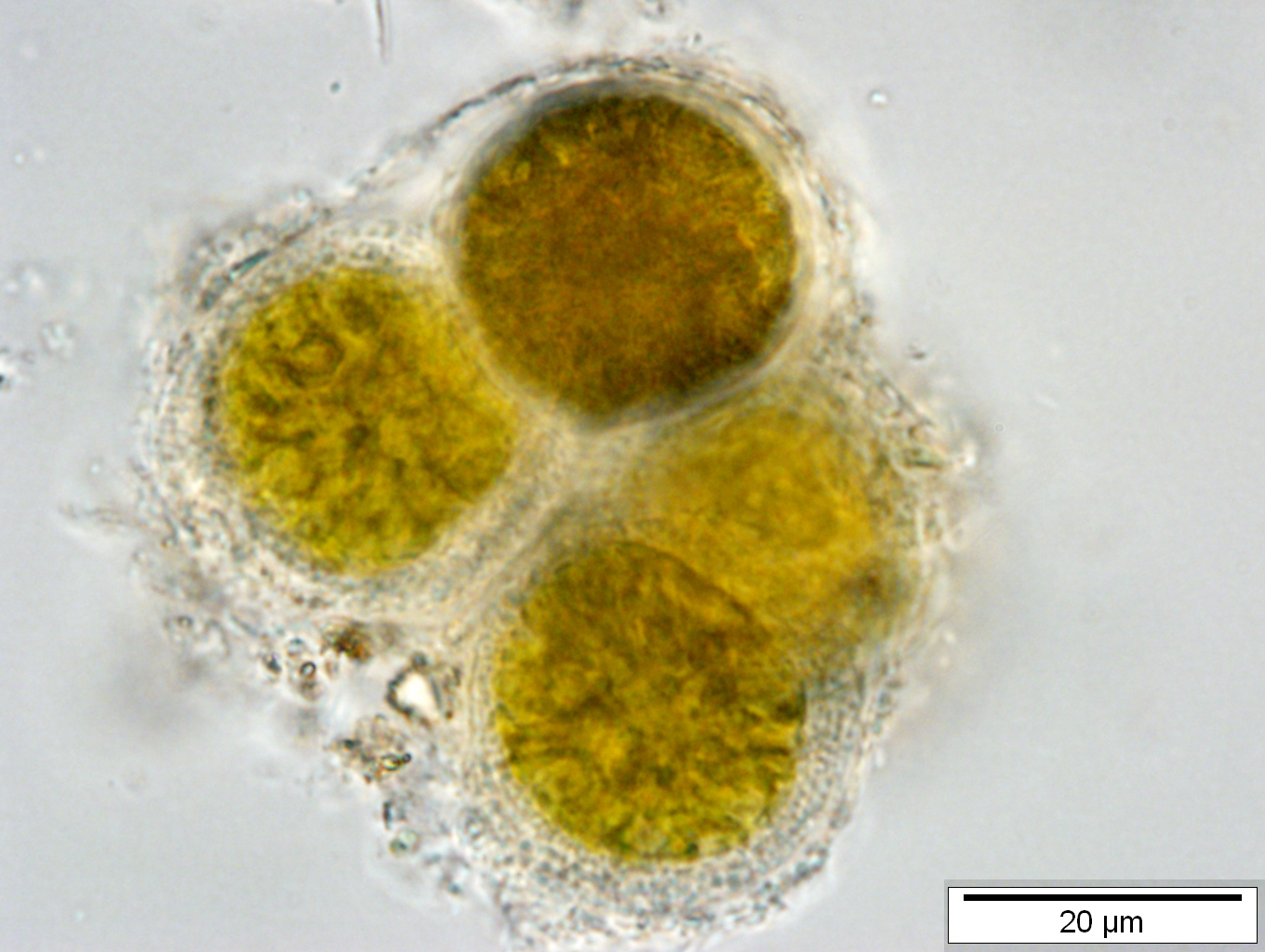

Supplement: Supplementary file 3 — Source data Fig. 1 [file 44321_2025_197_MOESM3_ESM.zip › Figure 1/raw data fig1B/10062020_233 with scale.tif]

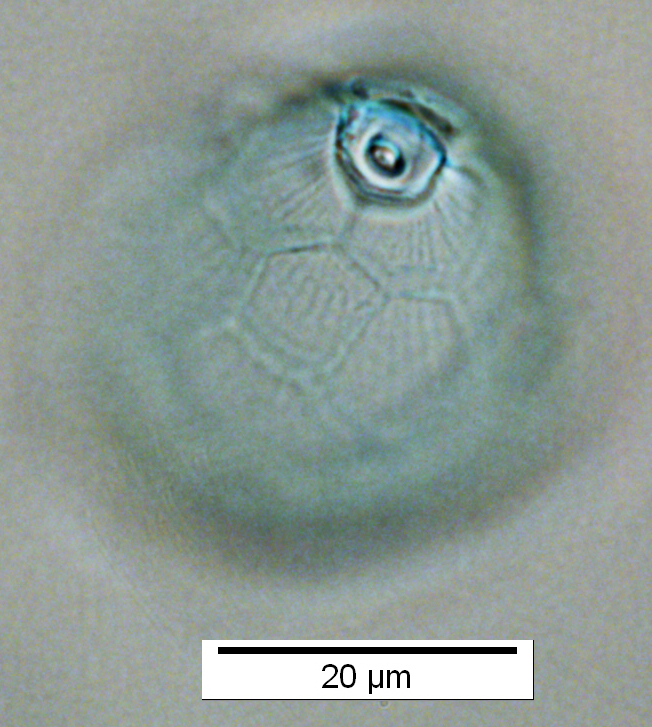

Supplement: Supplementary file 3 — Source data Fig. 1 [file 44321_2025_197_MOESM3_ESM.zip › Figure 1/raw data fig1B/10062020_308 modif.tif]

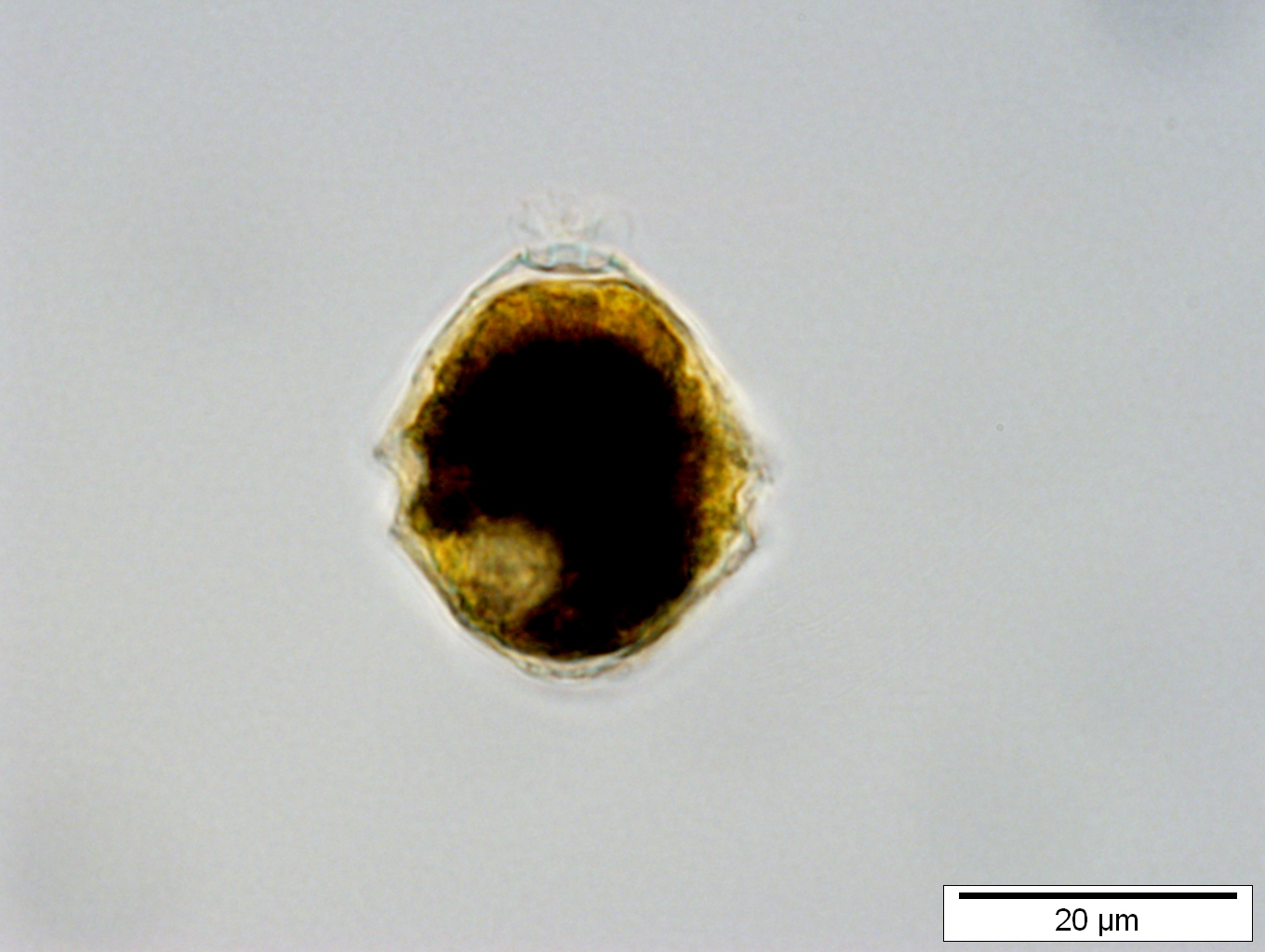

Supplement: Supplementary file 3 — Source data Fig. 1 [file 44321_2025_197_MOESM3_ESM.zip › Figure 1/raw data fig1B/10062020_187.jpg]

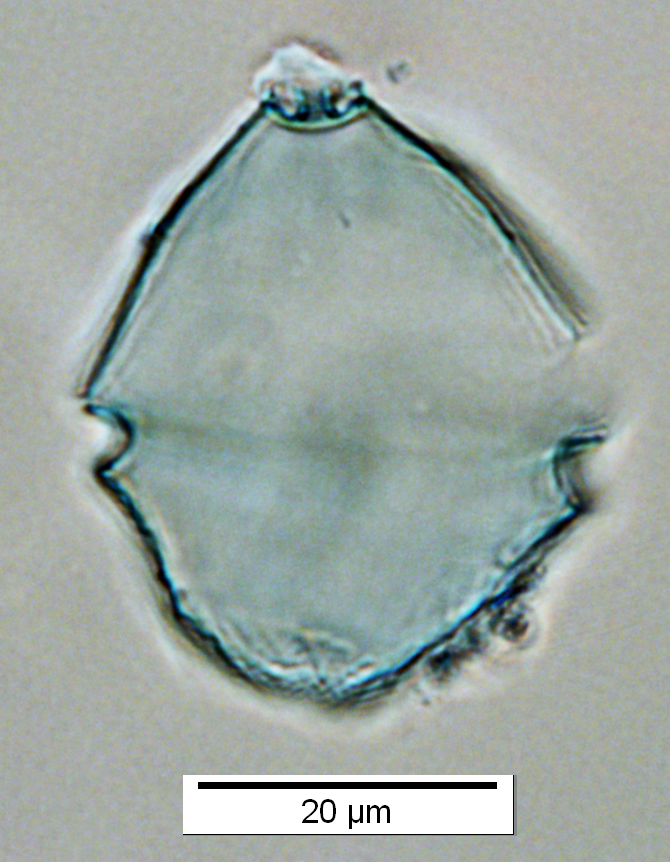

Supplement: Supplementary file 3 — Source data Fig. 1 [file 44321_2025_197_MOESM3_ESM.zip › Figure 1/raw data fig1B/10062020_322 modified.tif]

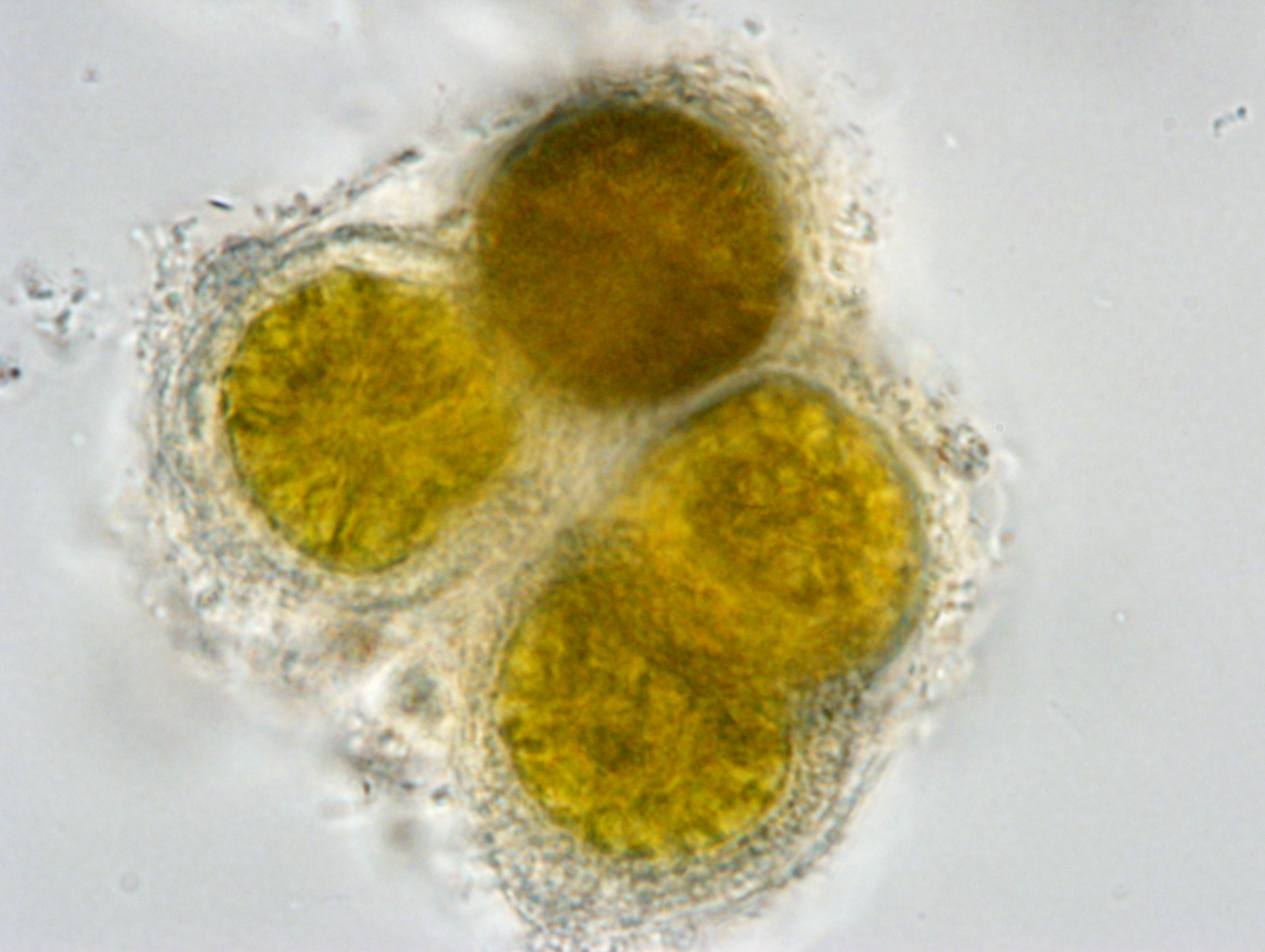

Supplement: Supplementary file 3 — Source data Fig. 1 [file 44321_2025_197_MOESM3_ESM.zip › Figure 1/raw data fig1B/10062020_231.jpg]

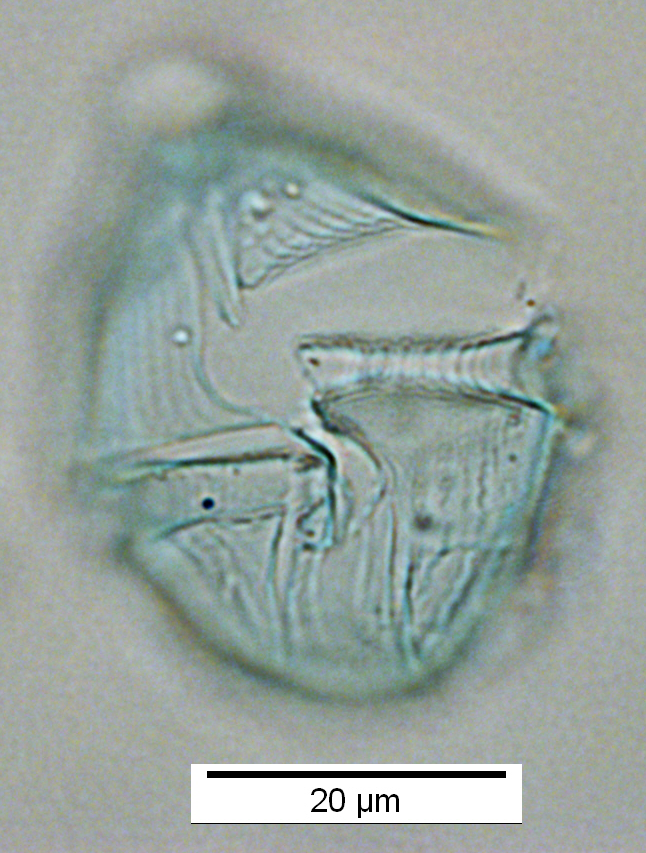

Supplement: Supplementary file 3 — Source data Fig. 1 [file 44321_2025_197_MOESM3_ESM.zip › Figure 1/raw data fig1B/10062020_320.tif]

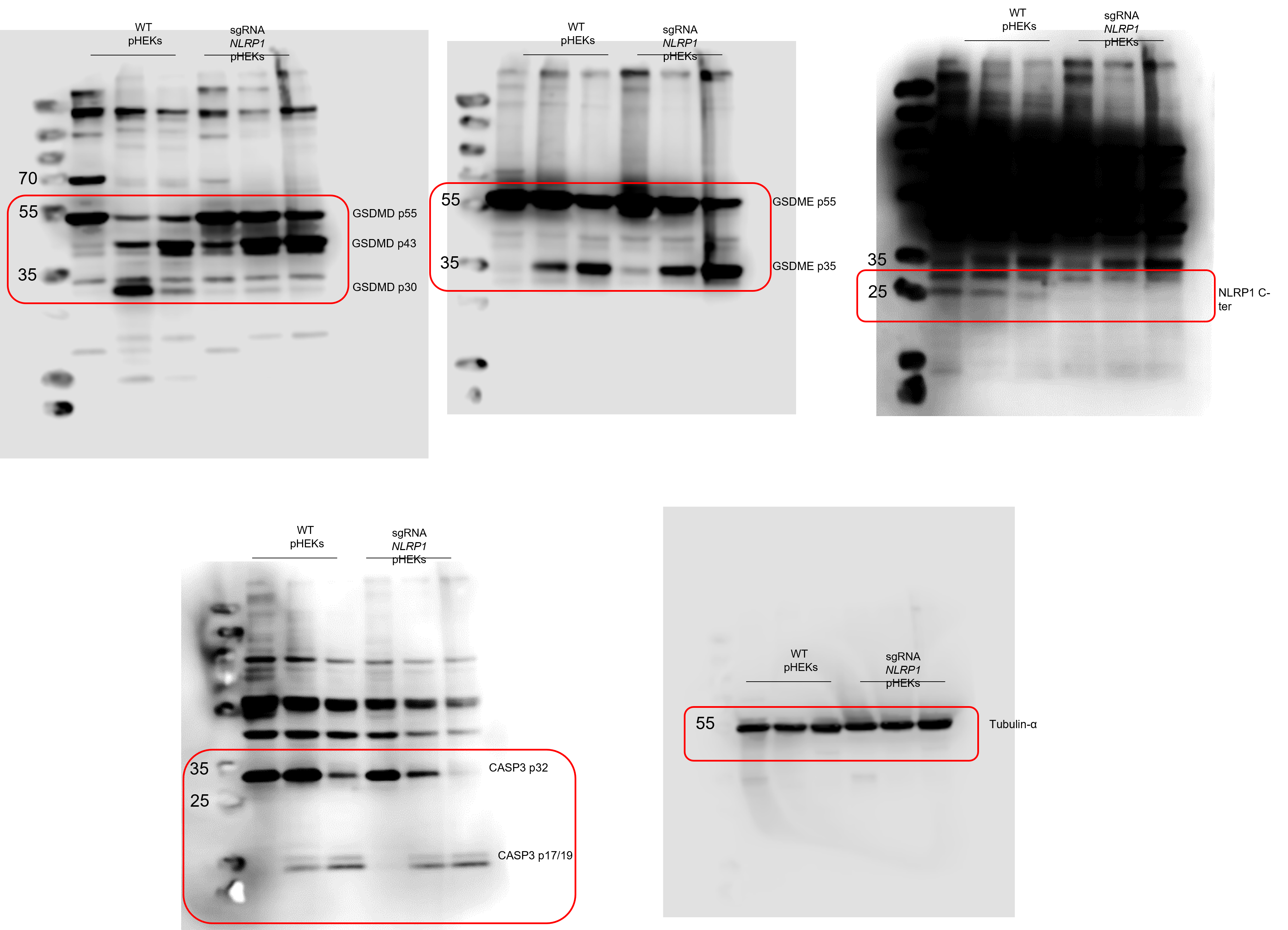

Supplement: Supplementary file 4 — Source data Fig. 2 [file 44321_2025_197_MOESM4_ESM.zip › Figure 2/2D/Blot membranes.tif]

## Source data Fig 2B

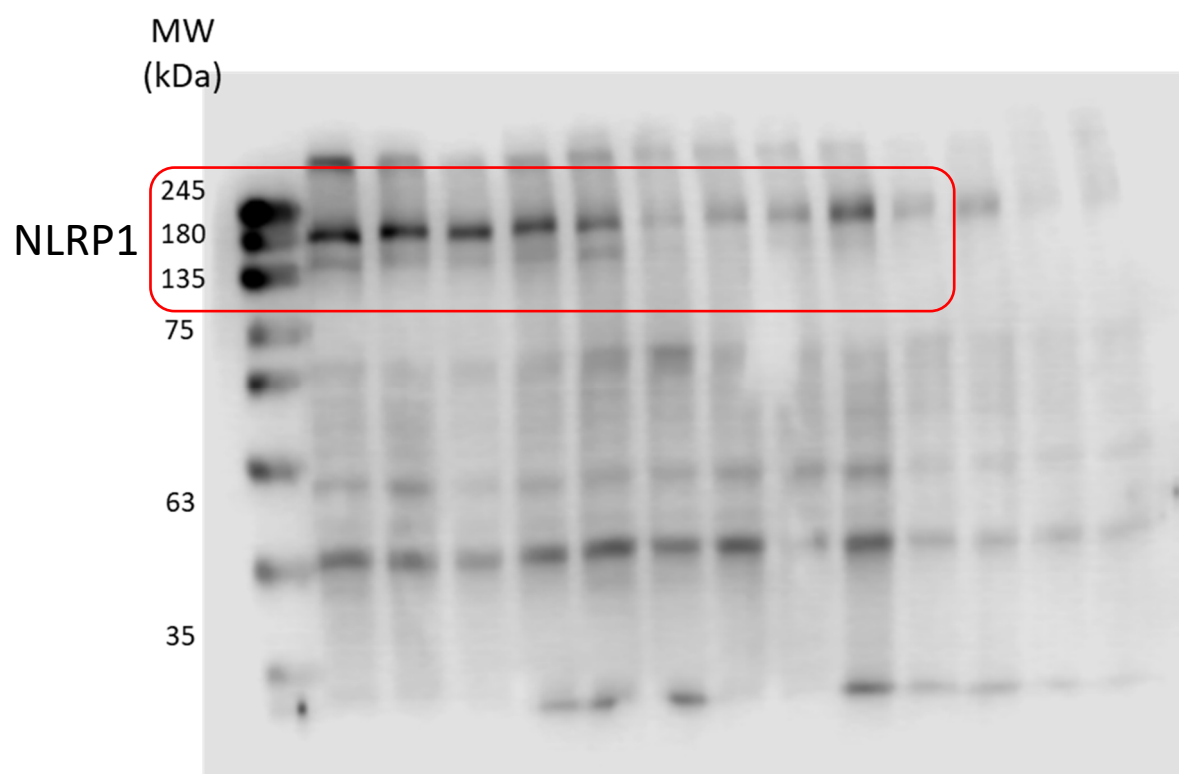

Tubulin- $\alpha$

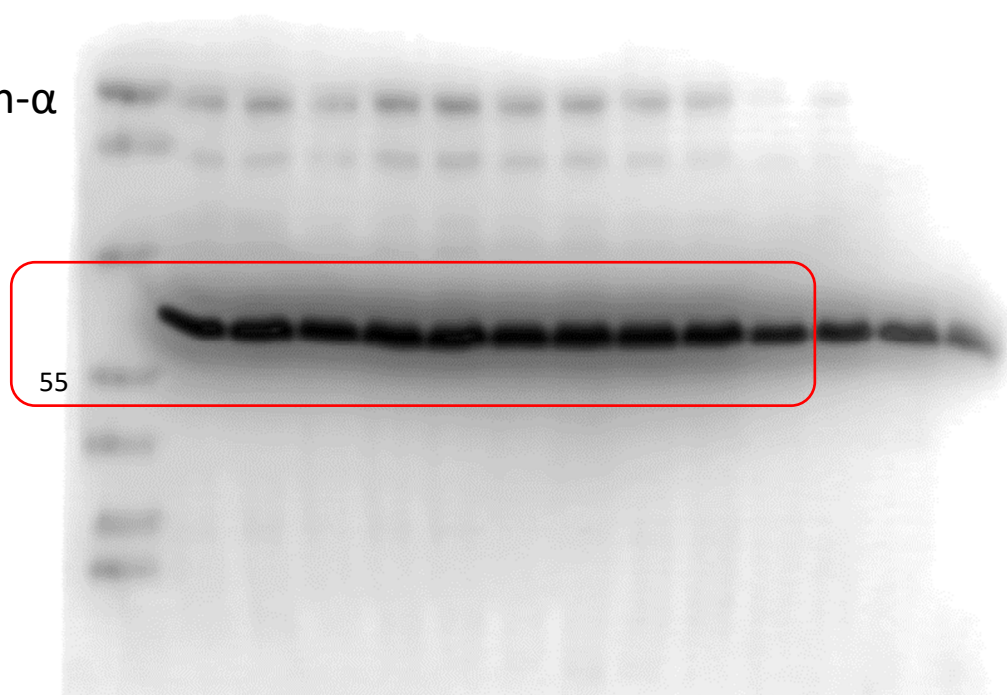

Supplement: Supplementary file 4 — Source data Fig. 2 [file 44321_2025_197_MOESM4_ESM.zip › Figure 2/2B/Immunoblots.pdf]

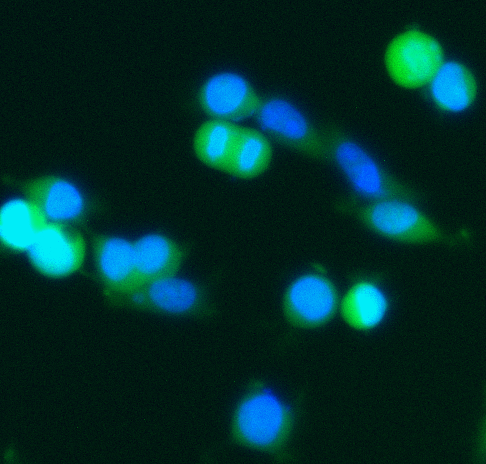

Supplement: Supplementary file 4 — Source data Fig. 2 [file 44321_2025_197_MOESM4_ESM.zip › Figure 2/2A/ASC GFP/RGB ASC GFP-selected area.tif]

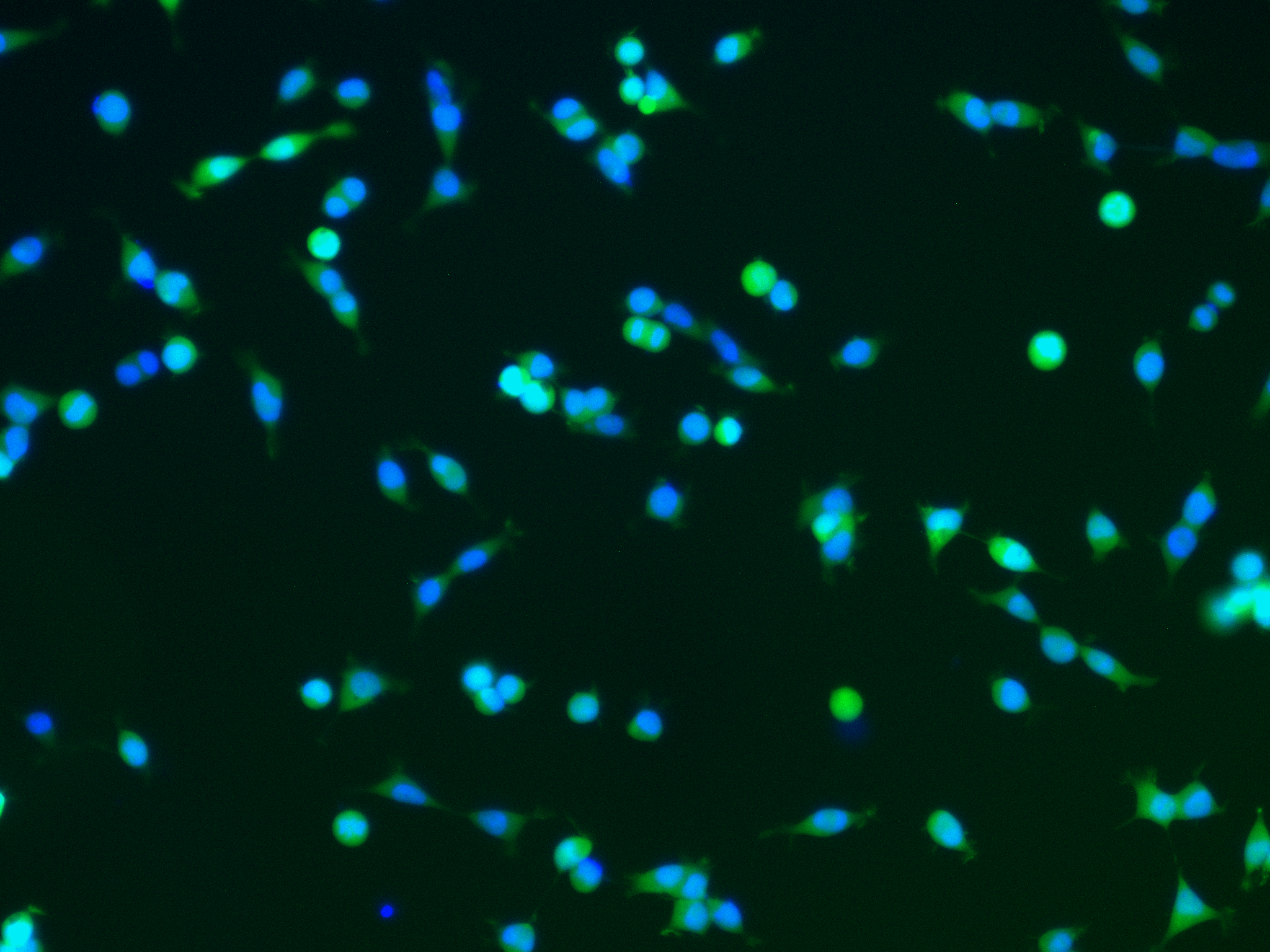

Supplement: Supplementary file 4 — Source data Fig. 2 [file 44321_2025_197_MOESM4_ESM.zip › Figure 2/2A/ASC GFP/RGB ASC GFP.tif]

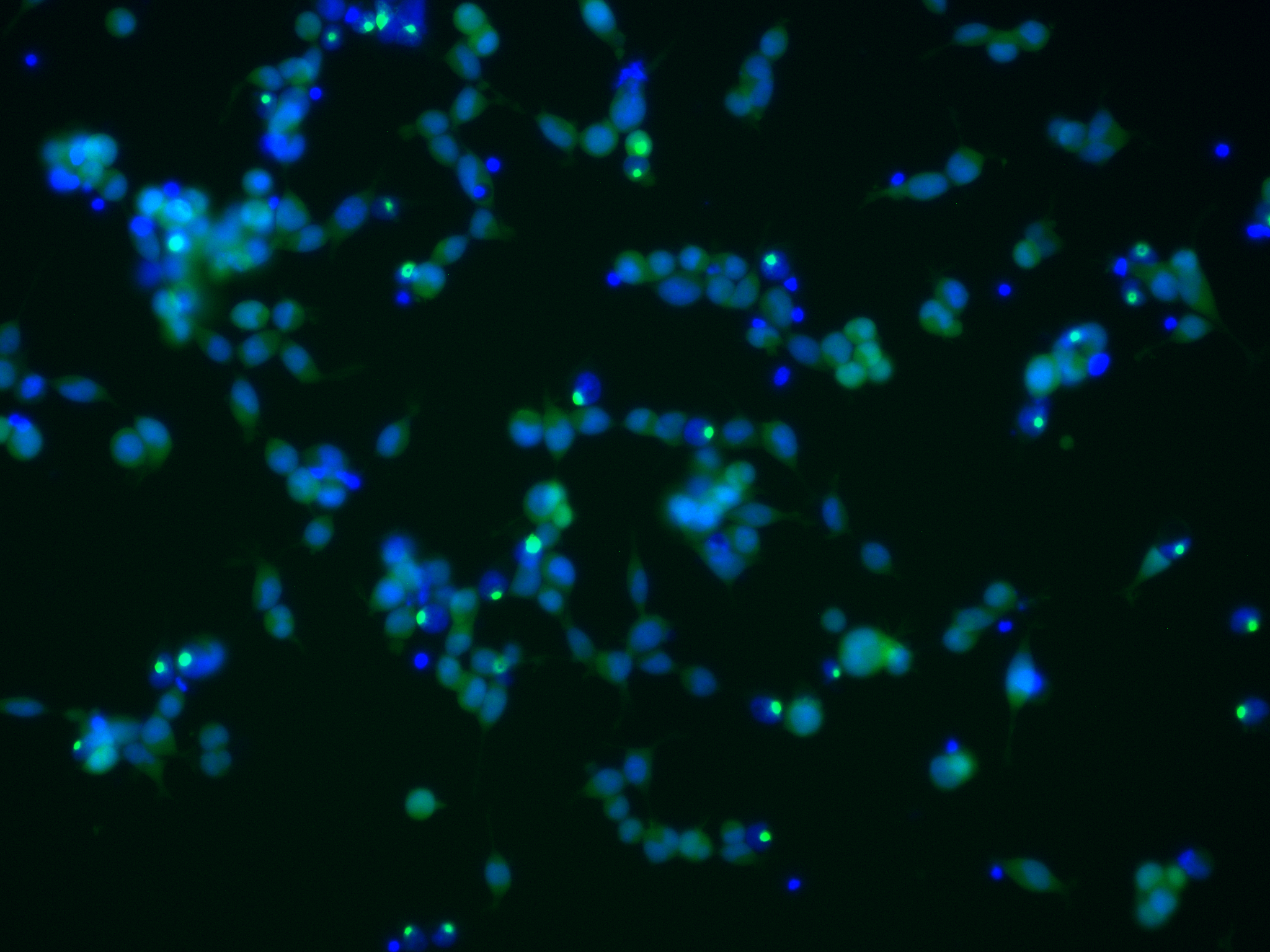

Supplement: Supplementary file 4 — Source data Fig. 2 [file 44321_2025_197_MOESM4_ESM.zip › Figure 2/2A/NLRP1-ASC GFP/NLRP1ASC GFP-RGB.tif]

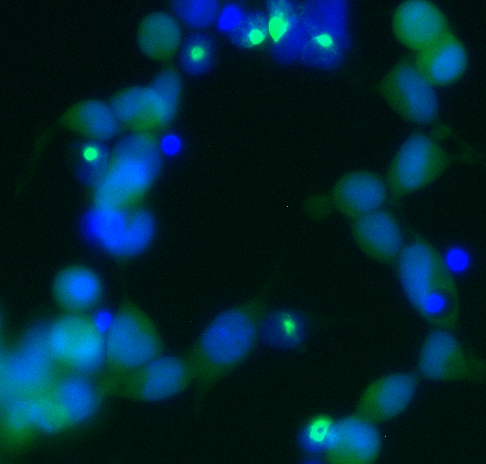

Supplement: Supplementary file 4 — Source data Fig. 2 [file 44321_2025_197_MOESM4_ESM.zip › Figure 2/2A/NLRP1-ASC GFP/NLRP1ASC GFP-RGB selected area.tif]

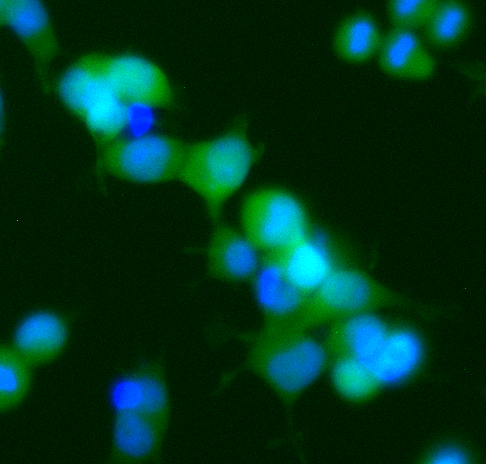

Supplement: Supplementary file 4 — Source data Fig. 2 [file 44321_2025_197_MOESM4_ESM.zip › Figure 2/2A/NLRP10-ASC GFP/NLRP10 ASC GFP- RGB selected area.tif]

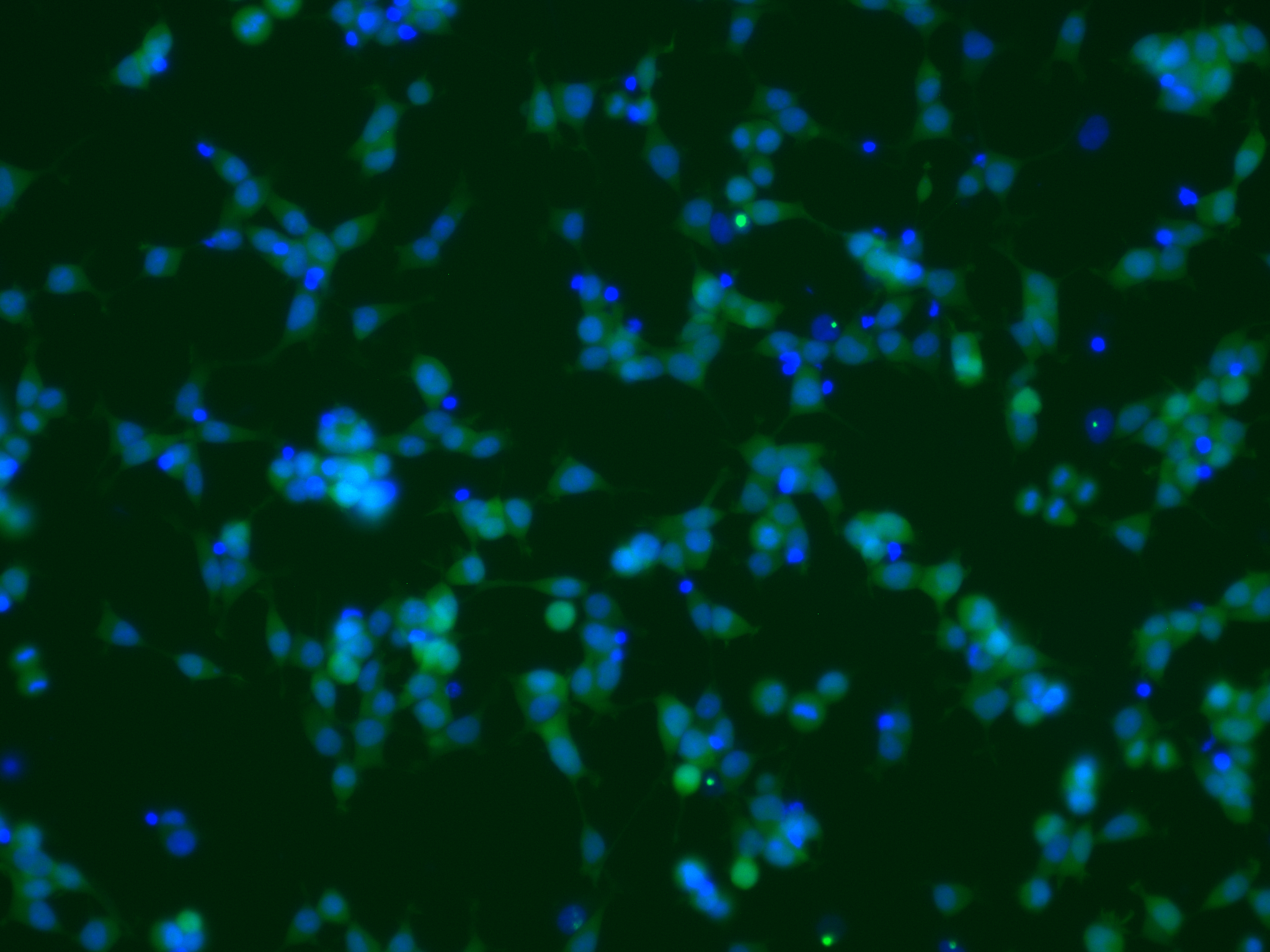

Supplement: Supplementary file 4 — Source data Fig. 2 [file 44321_2025_197_MOESM4_ESM.zip › Figure 2/2A/NLRP10-ASC GFP/NLRP10 ASC GFP- RGB.tif]

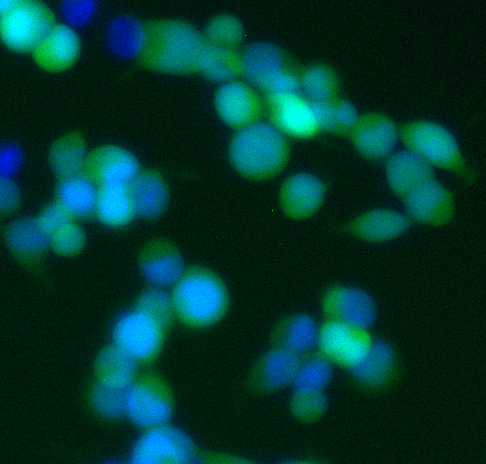

Supplement: Supplementary file 4 — Source data Fig. 2 [file 44321_2025_197_MOESM4_ESM.zip › Figure 2/2A/AIM2 -ASC GFP/AIM2 ASC GFP-RGB selected area.tif]

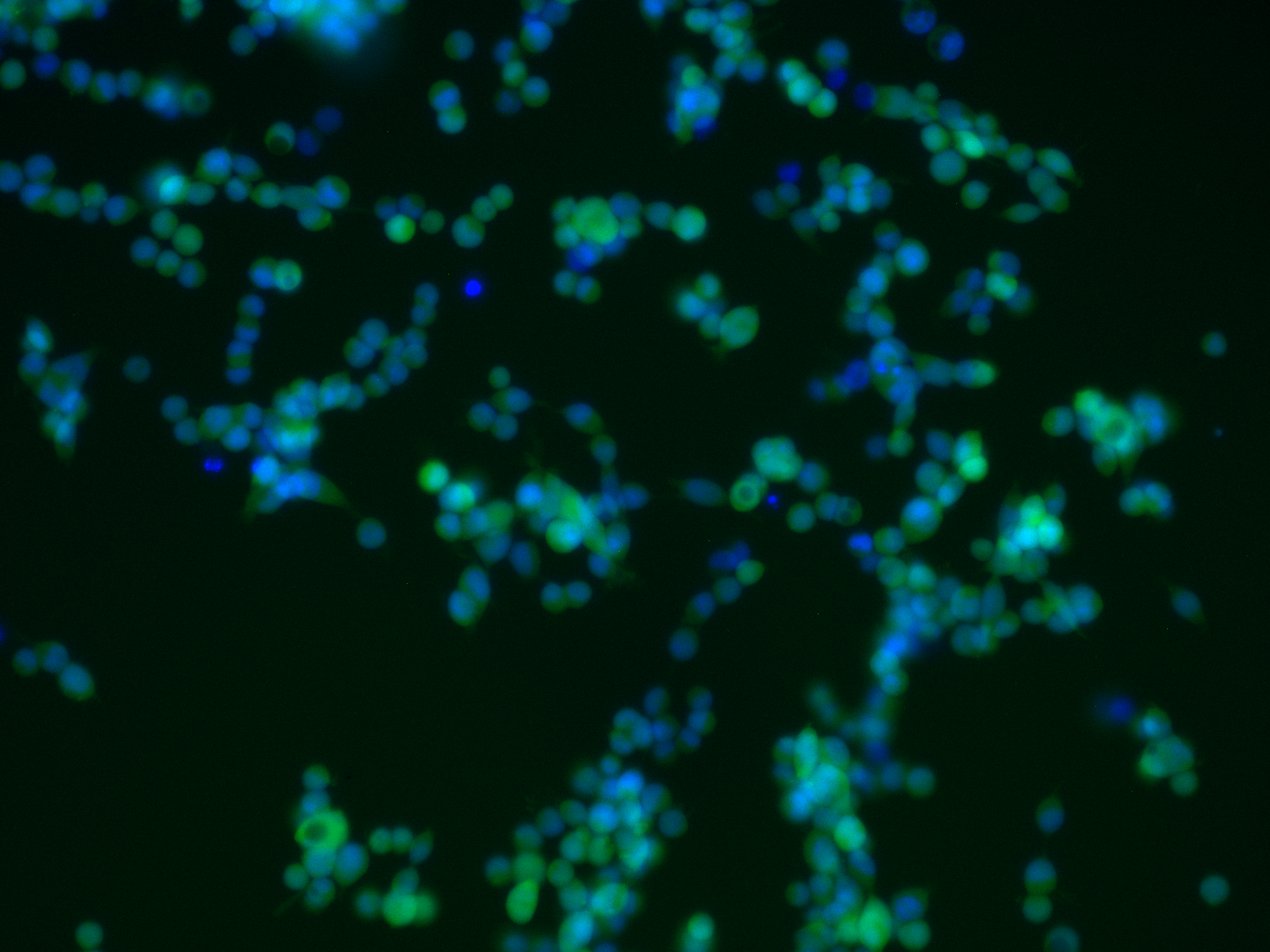

Supplement: Supplementary file 4 — Source data Fig. 2 [file 44321_2025_197_MOESM4_ESM.zip › Figure 2/2A/AIM2 -ASC GFP/AIM2 ASC GFP-RGB.tif]

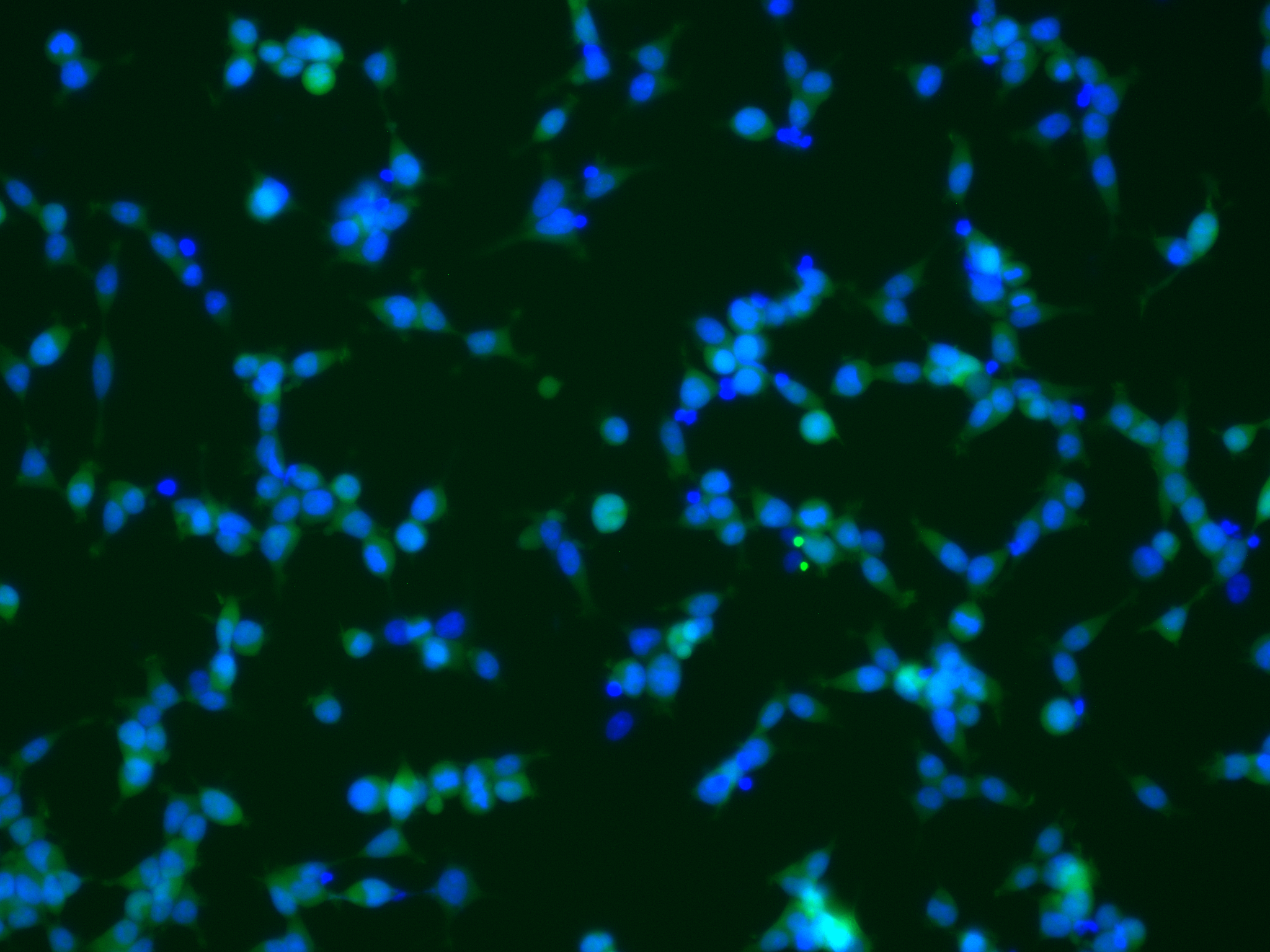

Supplement: Supplementary file 4 — Source data Fig. 2 [file 44321_2025_197_MOESM4_ESM.zip › Figure 2/2A/NLRP3-ASC GFP/NLRP3 ASC GFP-RGB.tif]

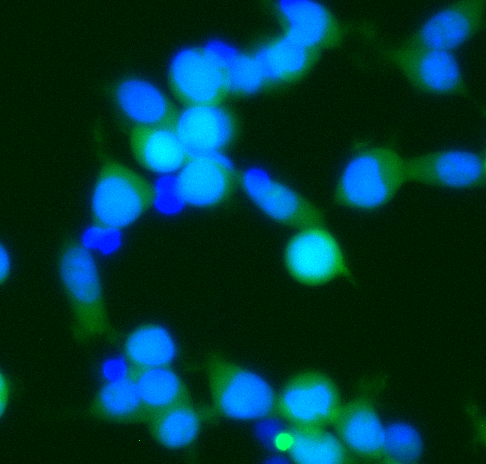

Supplement: Supplementary file 4 — Source data Fig. 2 [file 44321_2025_197_MOESM4_ESM.zip › Figure 2/2A/NLRP3-ASC GFP/NLRP3 ASC GFP-RGB selected area.tif]

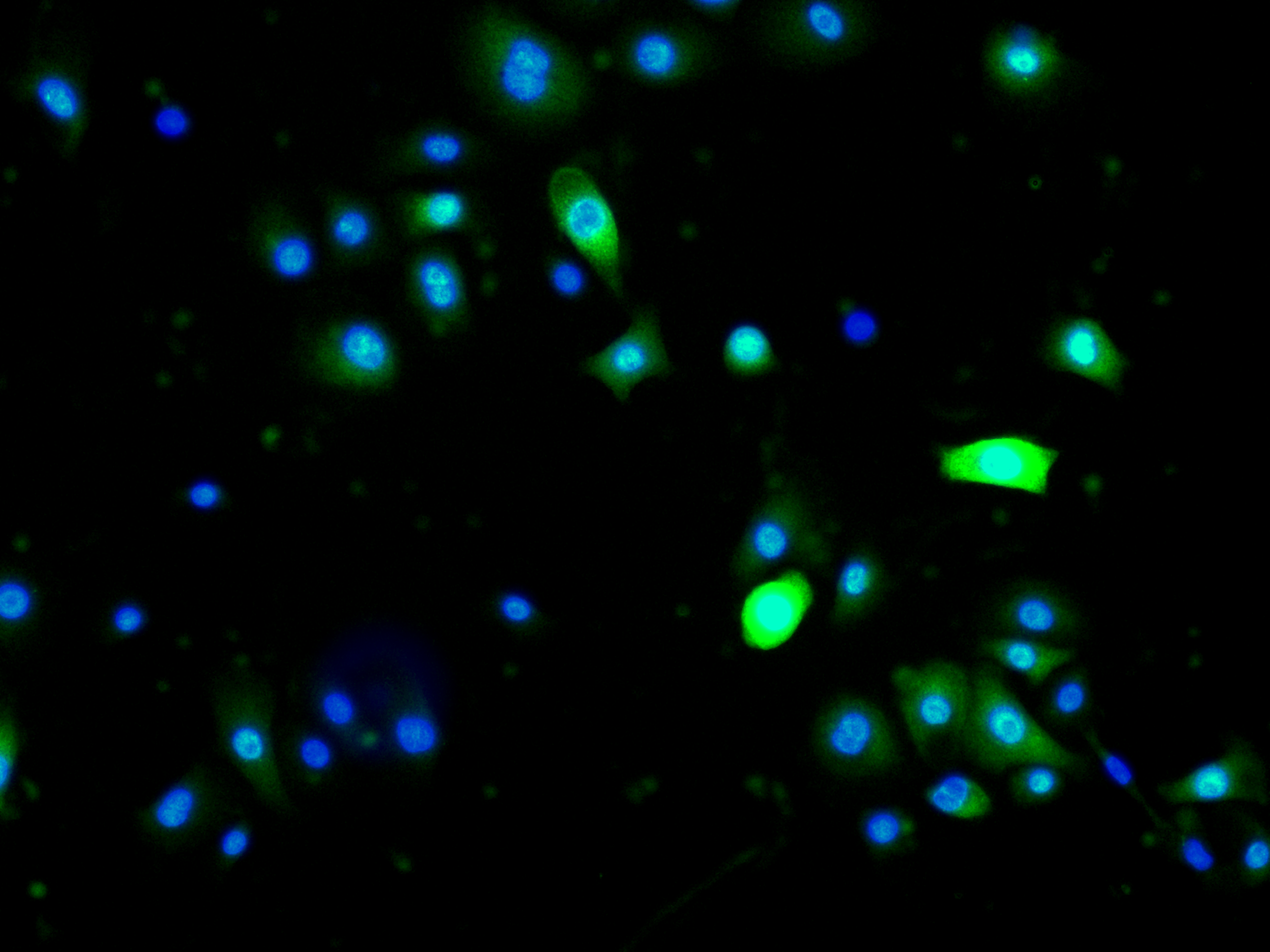

Supplement: Supplementary file 4 — Source data Fig. 2 [file 44321_2025_197_MOESM4_ESM.zip › Figure 2/2C/Images + quantifs/mock WT/Mock-RGB.tif]

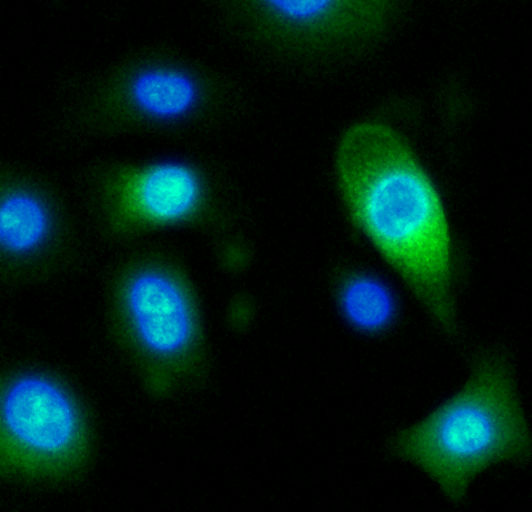

Supplement: Supplementary file 4 — Source data Fig. 2 [file 44321_2025_197_MOESM4_ESM.zip › Figure 2/2C/Images + quantifs/mock WT/Mock-RGB selected area.tif]

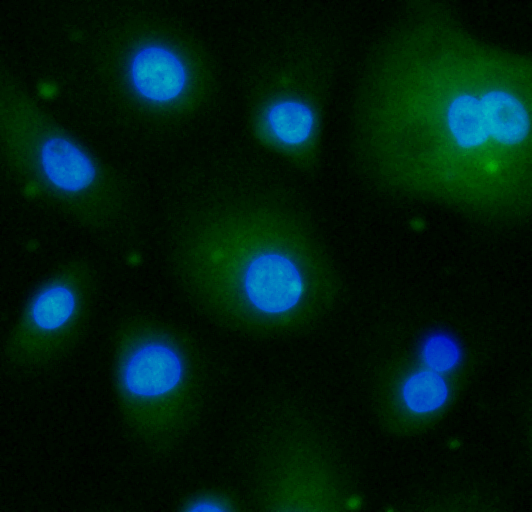

Supplement: Supplementary file 4 — Source data Fig. 2 [file 44321_2025_197_MOESM4_ESM.zip › Figure 2/2C/Images + quantifs/mock NLRP1 KO/RGB selected area.tif]

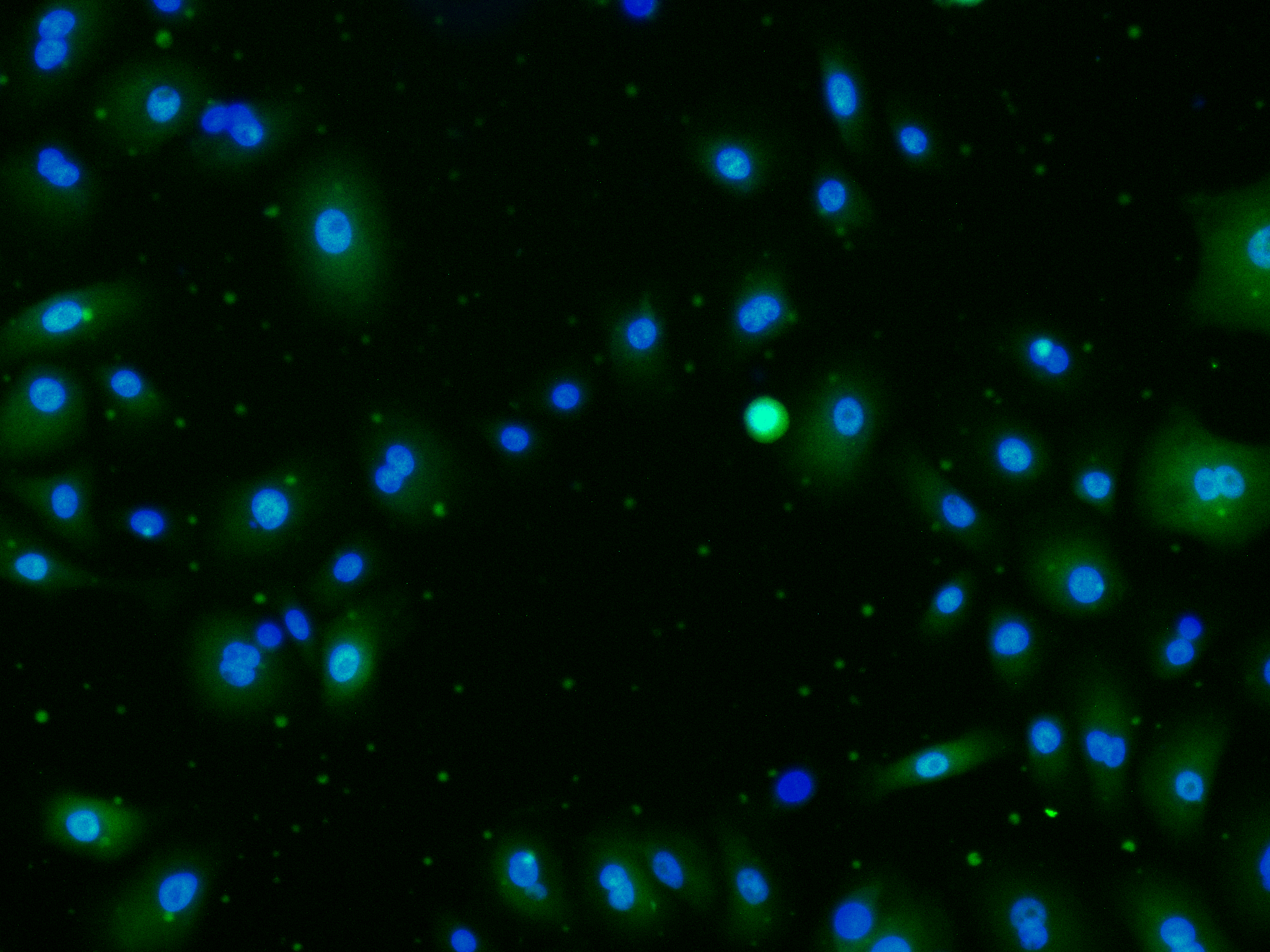

Supplement: Supplementary file 4 — Source data Fig. 2 [file 44321_2025_197_MOESM4_ESM.zip › Figure 2/2C/Images + quantifs/mock NLRP1 KO/RGB.tif]

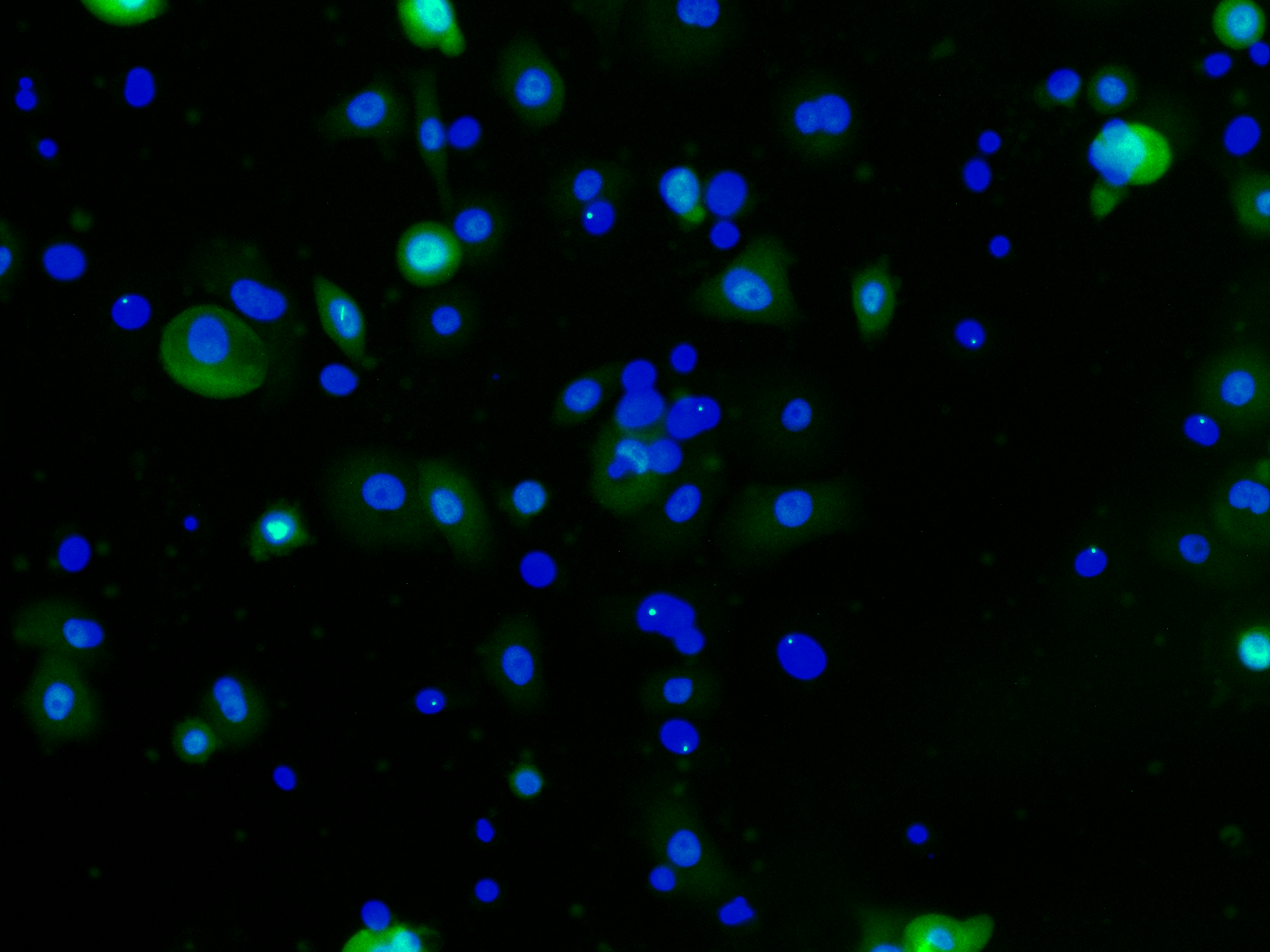

Supplement: Supplementary file 4 — Source data Fig. 2 [file 44321_2025_197_MOESM4_ESM.zip › Figure 2/2C/Images + quantifs/Portimine A WT/Portimine A - RGB.tif]

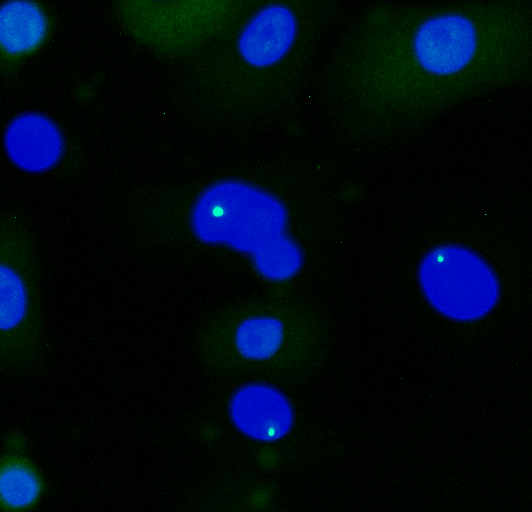

Supplement: Supplementary file 4 — Source data Fig. 2 [file 44321_2025_197_MOESM4_ESM.zip › Figure 2/2C/Images + quantifs/Portimine A WT/Poritmine A - RGB selected area.tif]

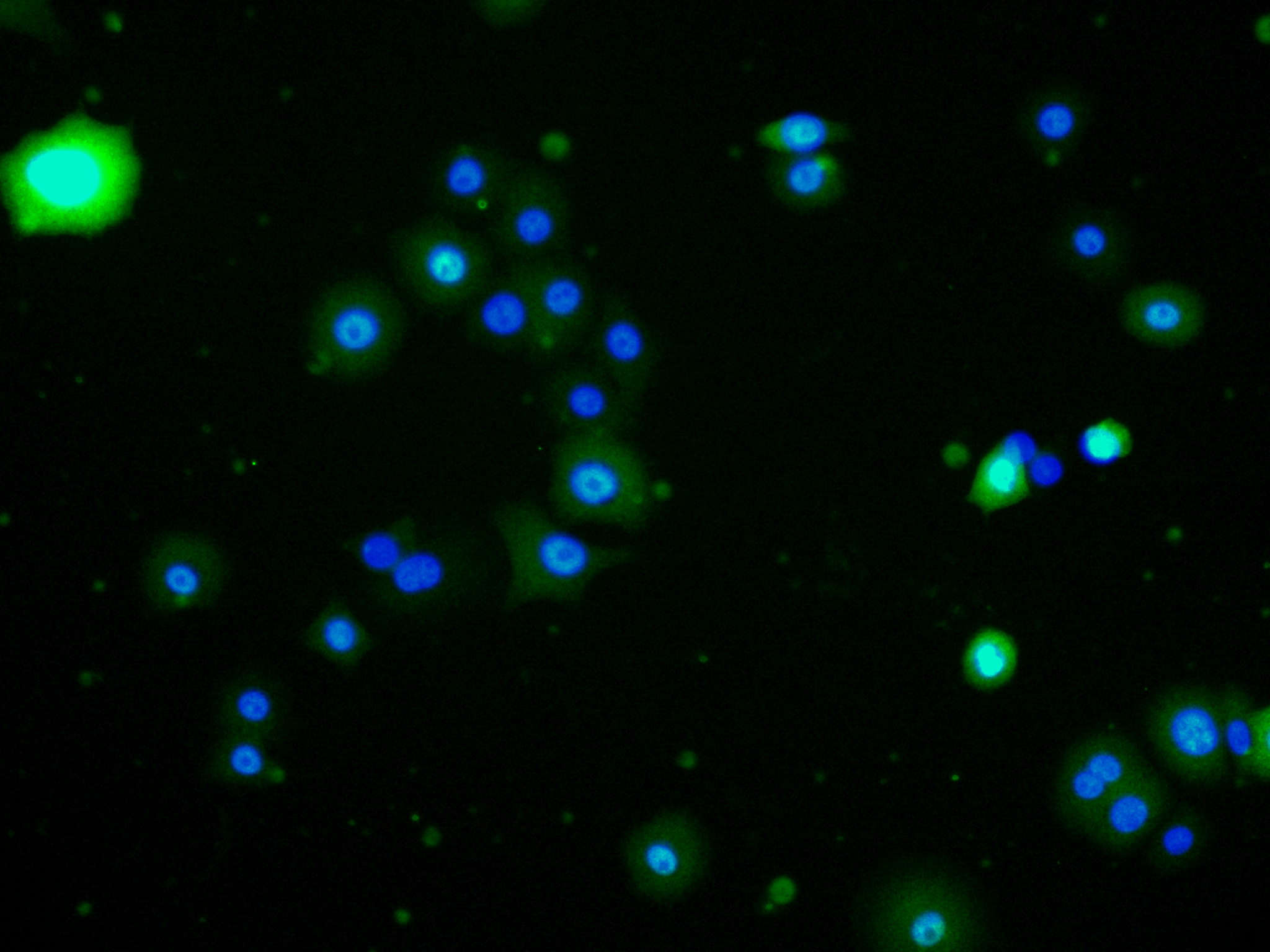

Supplement: Supplementary file 4 — Source data Fig. 2 [file 44321_2025_197_MOESM4_ESM.zip › Figure 2/2C/Images + quantifs/Portimine A NLRP1 KO/Portimine A - RGB.tif]

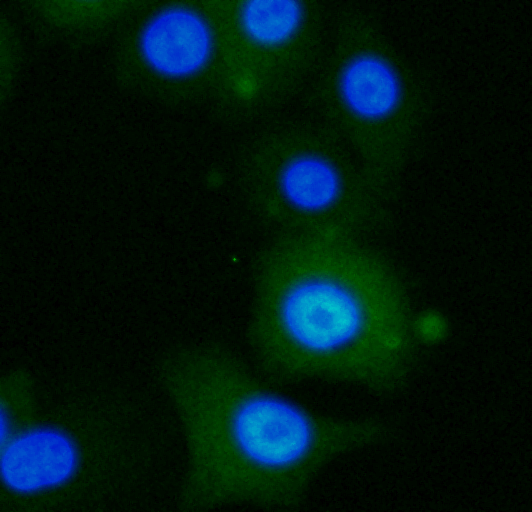

Supplement: Supplementary file 4 — Source data Fig. 2 [file 44321_2025_197_MOESM4_ESM.zip › Figure 2/2C/Images + quantifs/Portimine A NLRP1 KO/Portimine A - RGB selected area.tif]

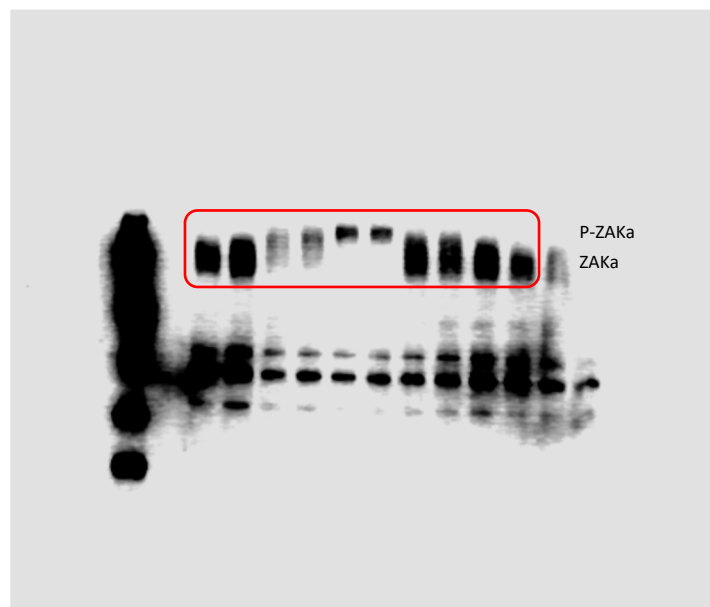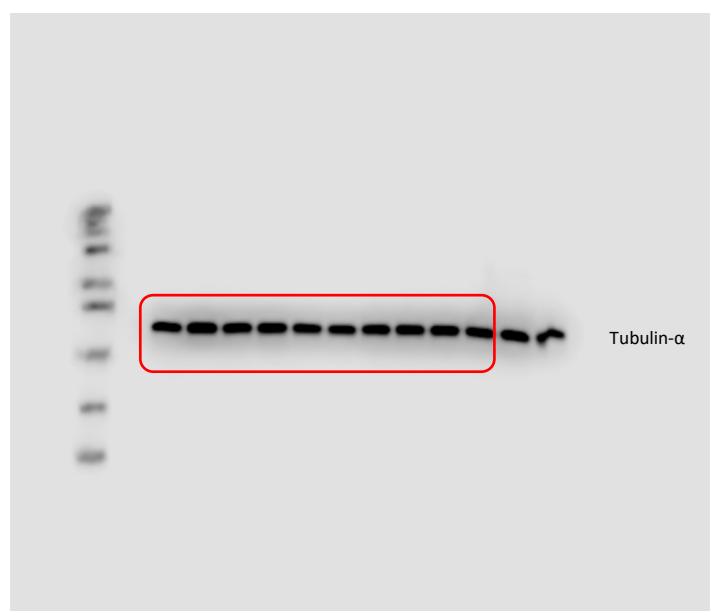

Supplement: Supplementary file 5 — Source data Fig. 3 [file 44321_2025_197_MOESM5_ESM.zip › Figure 3/3B/blotting membranes.pdf]

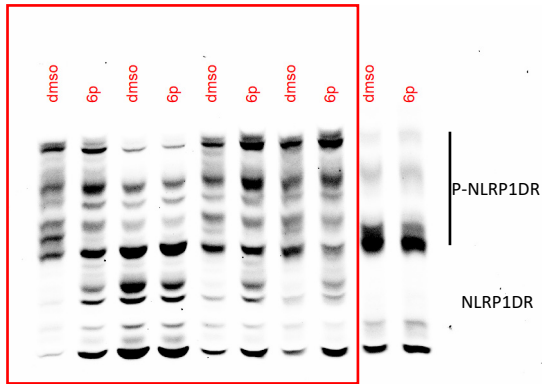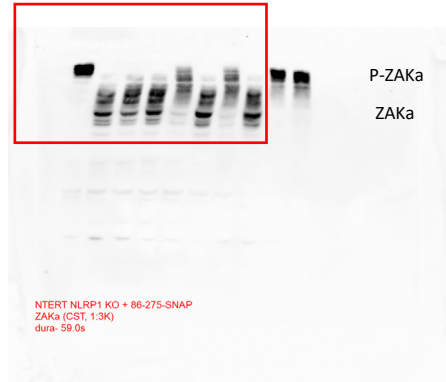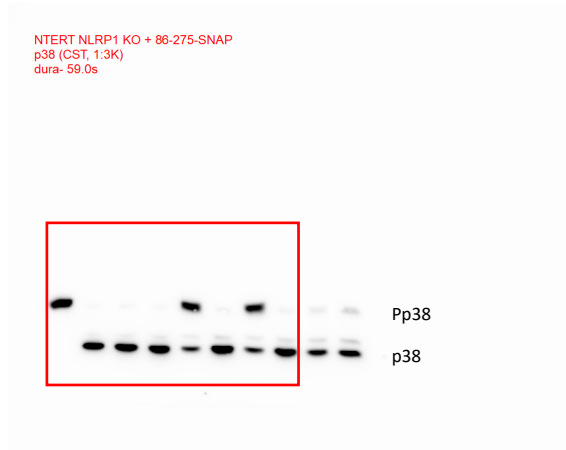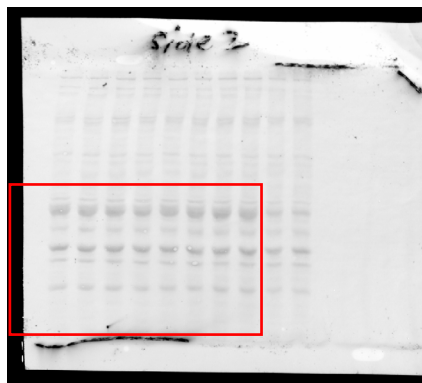

Ponceau

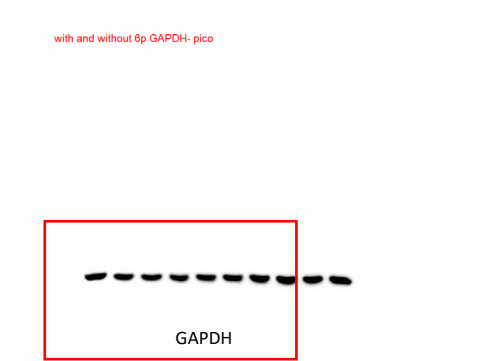

Supplement: Supplementary file 5 — Source data Fig. 3 [file 44321_2025_197_MOESM5_ESM.zip › Figure 3/3C/Immunoblotting membranes.pdf]

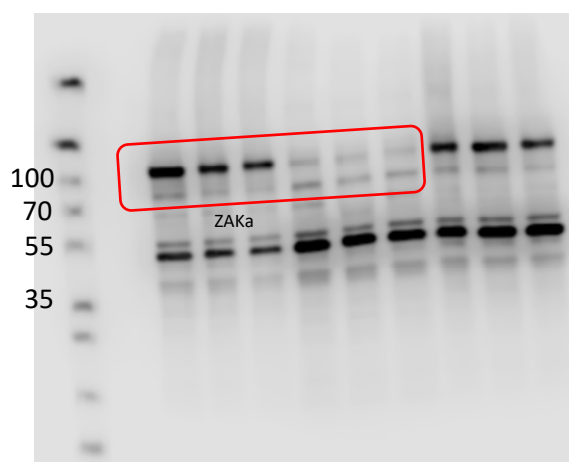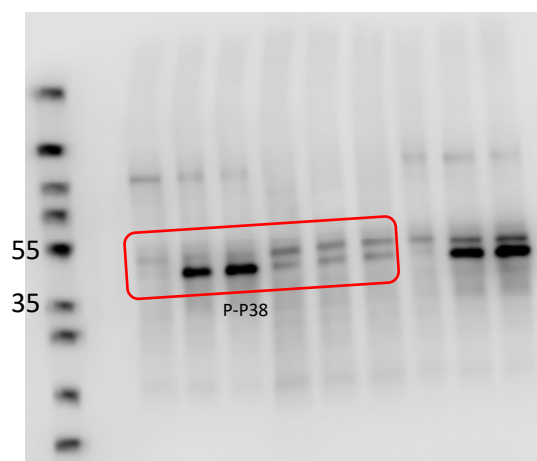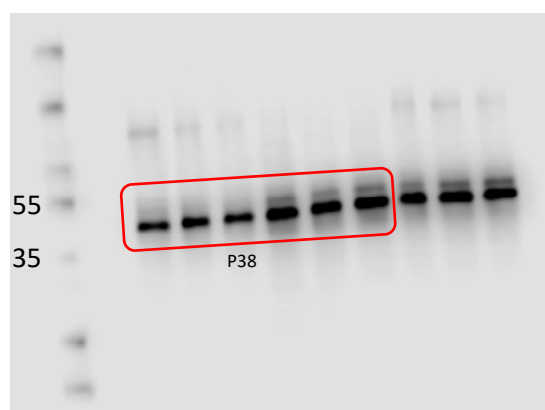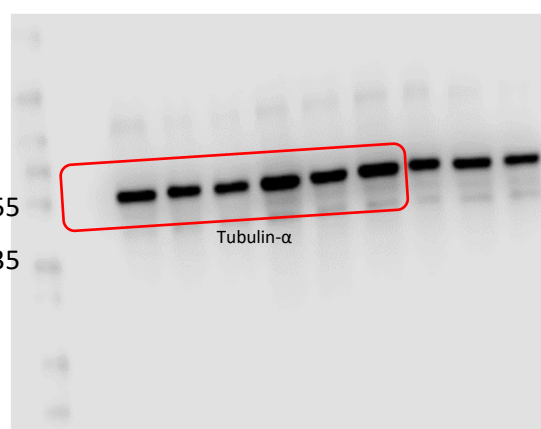

Supplement: Supplementary file 5 — Source data Fig. 3 [file 44321_2025_197_MOESM5_ESM.zip › Figure 3/3D/Immunoblotting membranes 3D.pdf]

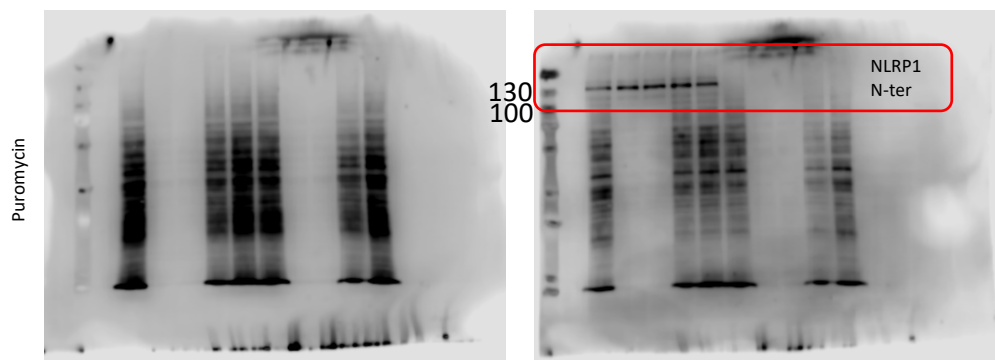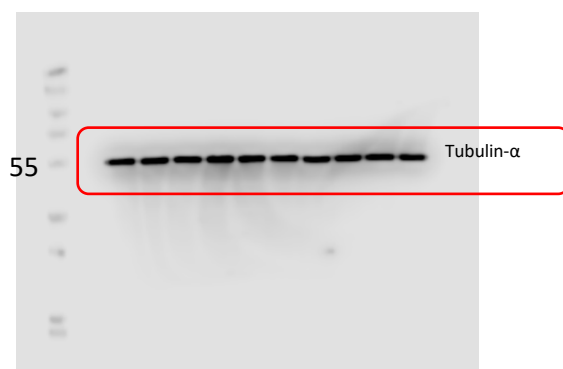

Supplement: Supplementary file 5 — Source data Fig. 3 [file 44321_2025_197_MOESM5_ESM.zip › Figure 3/3A/blotting membranes.pdf]

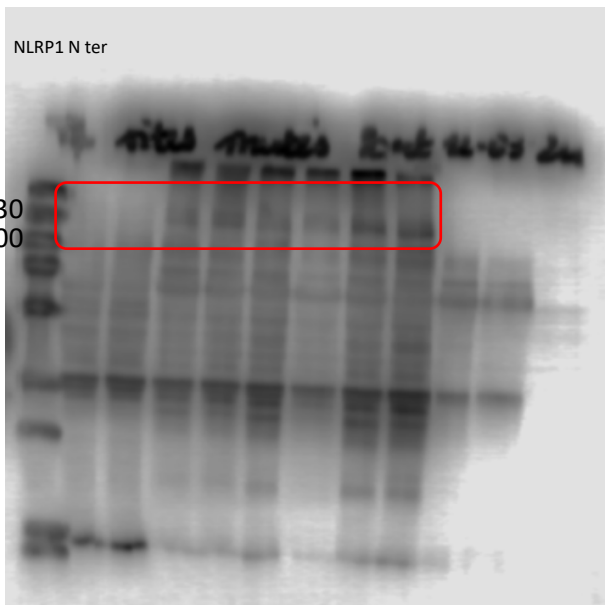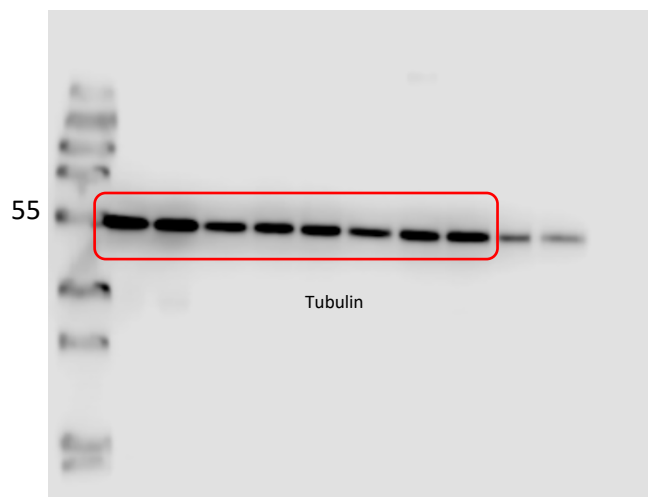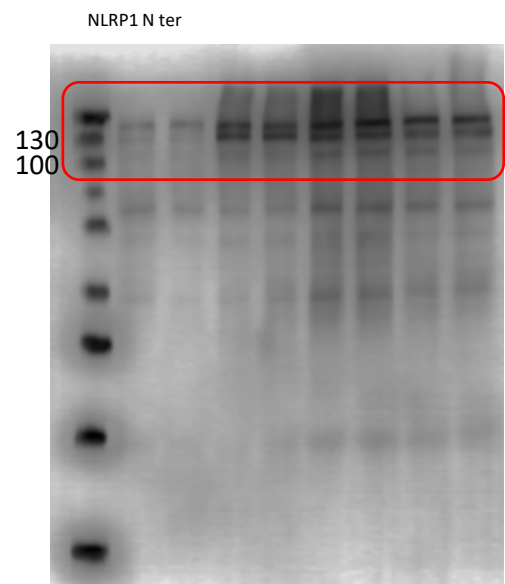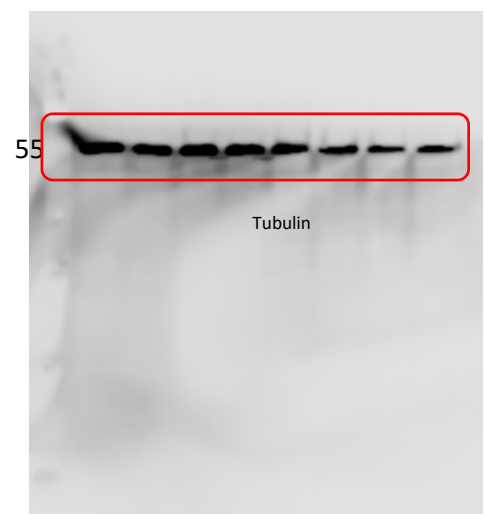

Supplement: Supplementary file 5 — Source data Fig. 3 [file 44321_2025_197_MOESM5_ESM.zip › Figure 3/3F/Immunoblotting membranes 3F.pdf]

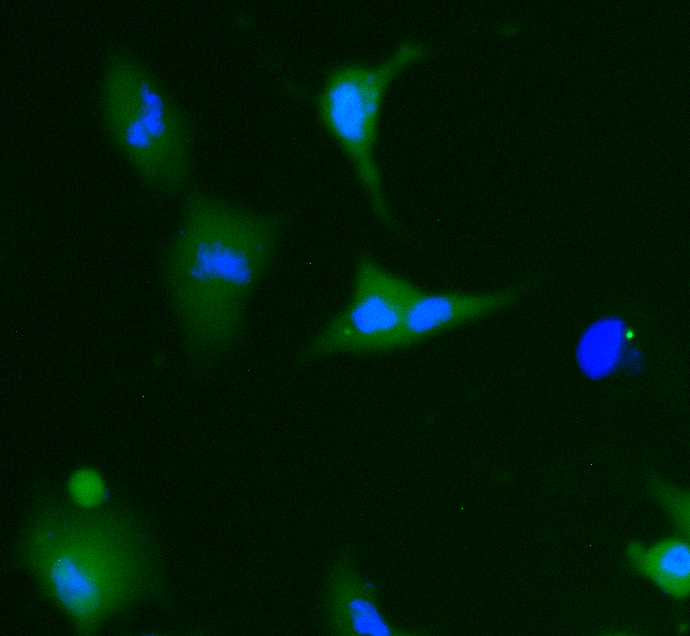

Supplement: Supplementary file 5 — Source data Fig. 3 [file 44321_2025_197_MOESM5_ESM.zip › Figure 3/3E/Mock + SB/Mock - RGB selected area.tif]

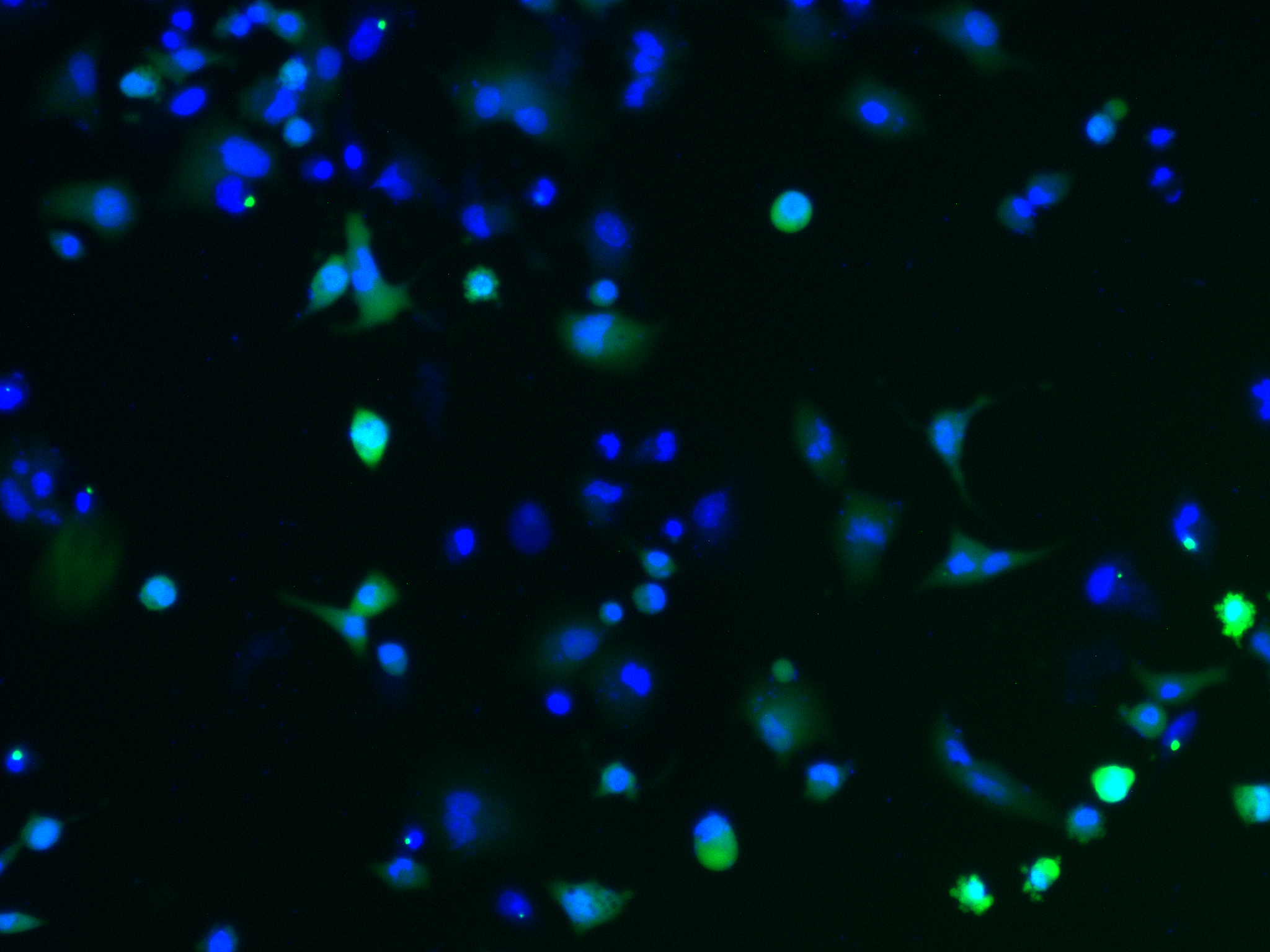

Supplement: Supplementary file 5 — Source data Fig. 3 [file 44321_2025_197_MOESM5_ESM.zip › Figure 3/3E/Mock + SB/Mock - RGB.tif]

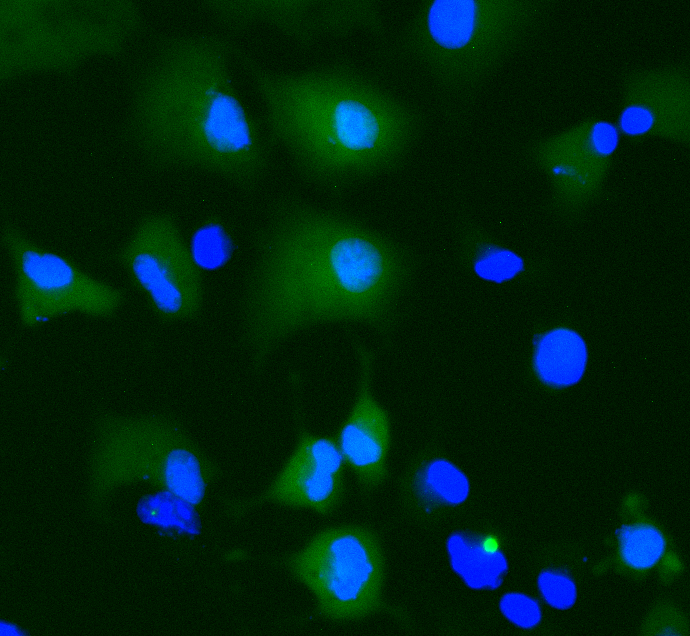

Supplement: Supplementary file 5 — Source data Fig. 3 [file 44321_2025_197_MOESM5_ESM.zip › Figure 3/3E/Mock/Mock - RGB selected area.tif]

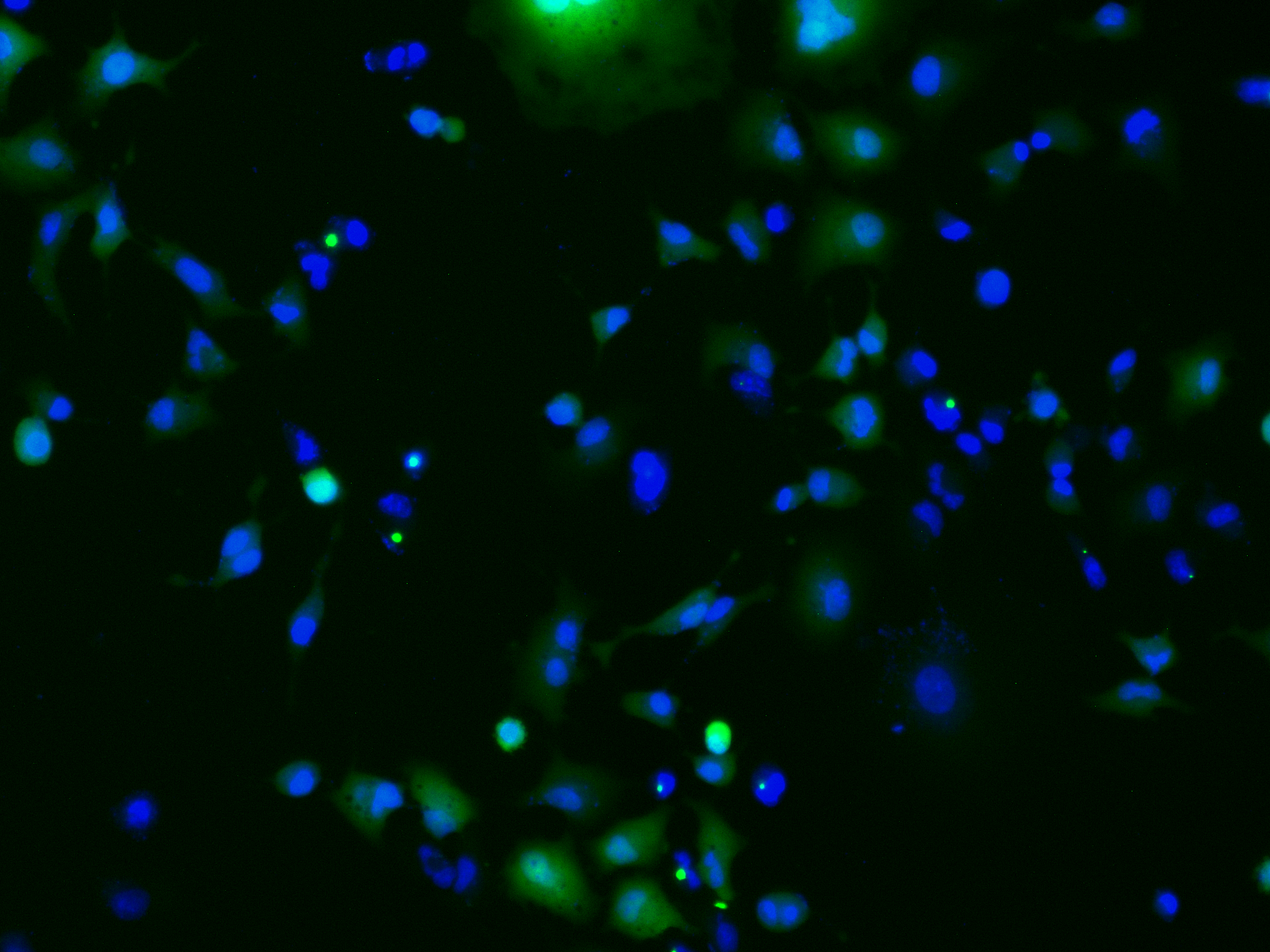

Supplement: Supplementary file 5 — Source data Fig. 3 [file 44321_2025_197_MOESM5_ESM.zip › Figure 3/3E/Mock/Mock - RGB.tif]

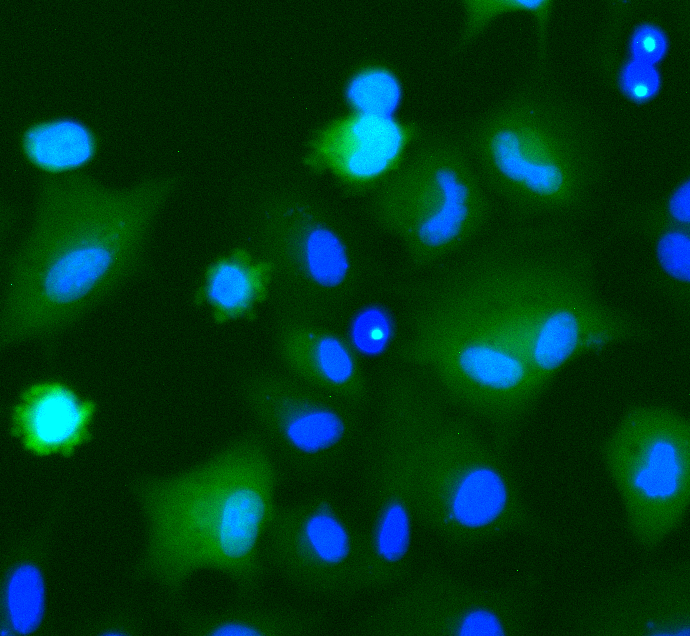

Supplement: Supplementary file 5 — Source data Fig. 3 [file 44321_2025_197_MOESM5_ESM.zip › Figure 3/3E/Portimine A + SB/Portimine A + SB - RGB selected area.tif]

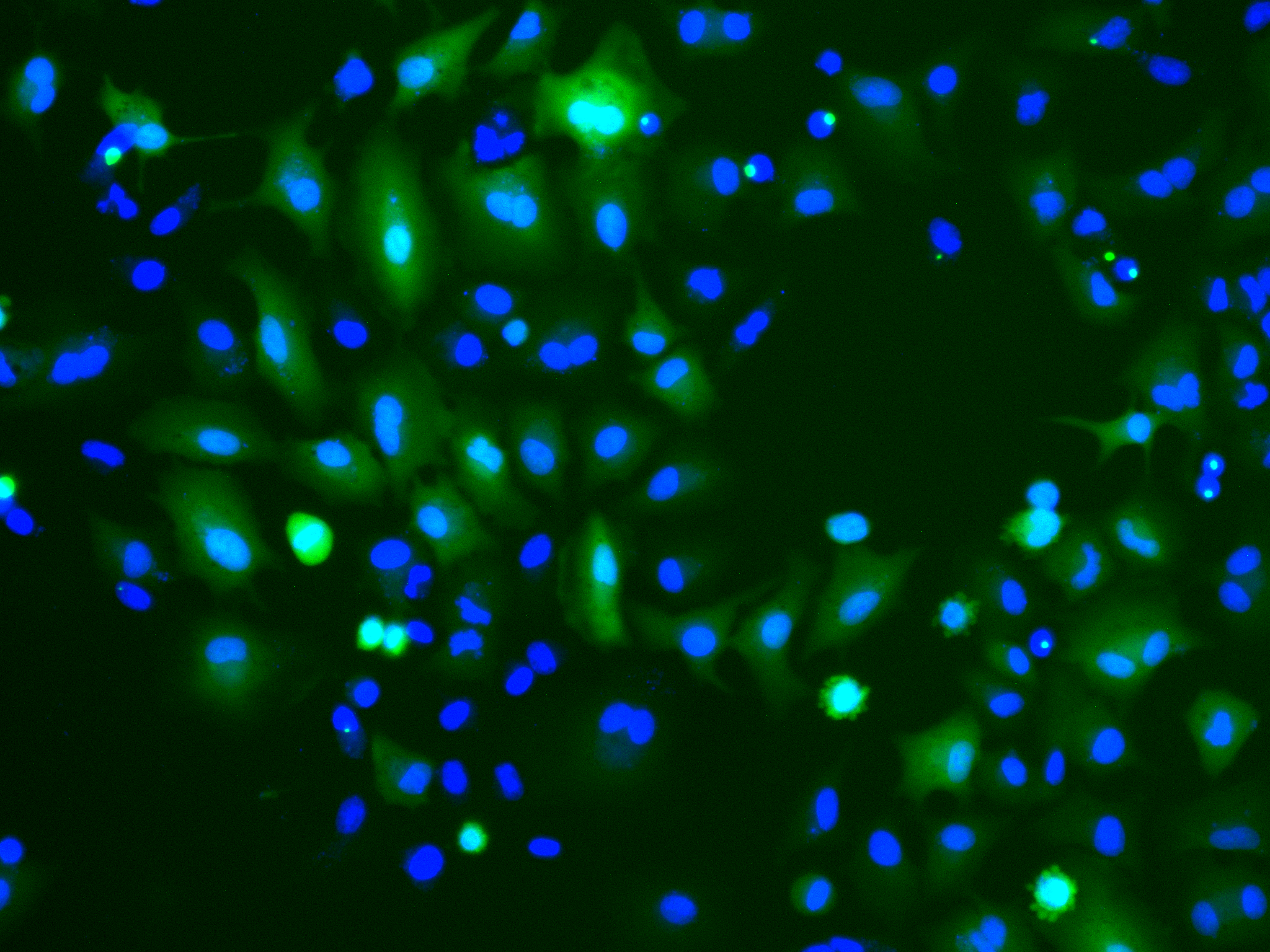

Supplement: Supplementary file 5 — Source data Fig. 3 [file 44321_2025_197_MOESM5_ESM.zip › Figure 3/3E/Portimine A + SB/Portimine A + SB - RGB.tif]

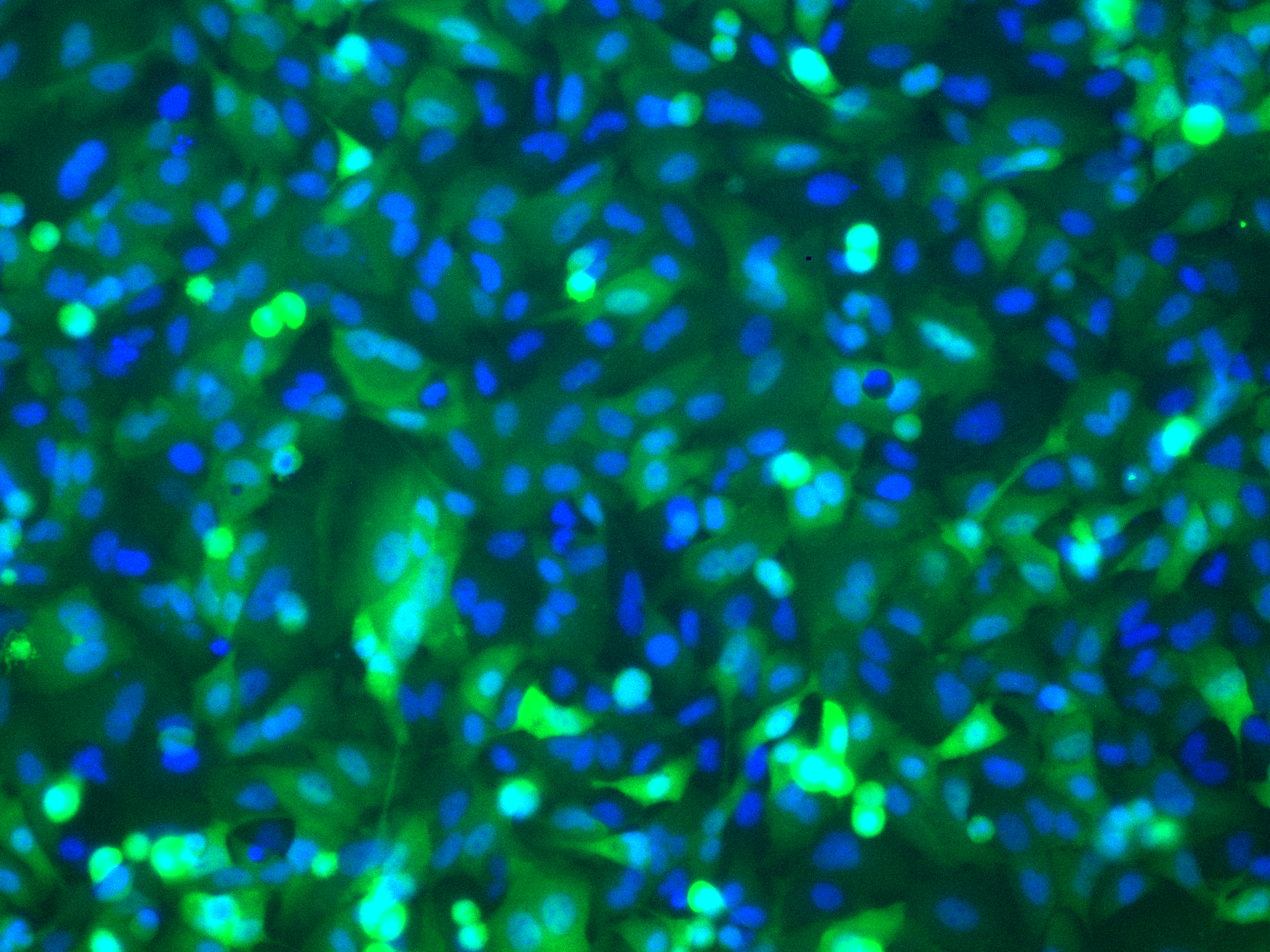

Supplement: Supplementary file 5 — Source data Fig. 3 [file 44321_2025_197_MOESM5_ESM.zip › Figure 3/3E/Anisomycin + SB/Anisomycin + SB - RGB.tif]

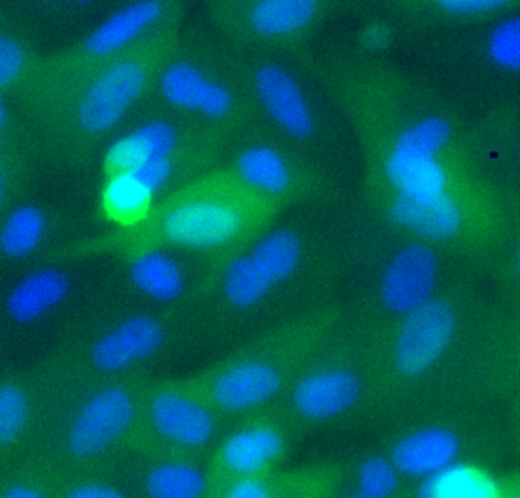

Supplement: Supplementary file 5 — Source data Fig. 3 [file 44321_2025_197_MOESM5_ESM.zip › Figure 3/3E/Anisomycin + SB/Anisomycin + SB - RGB selected area.tif]

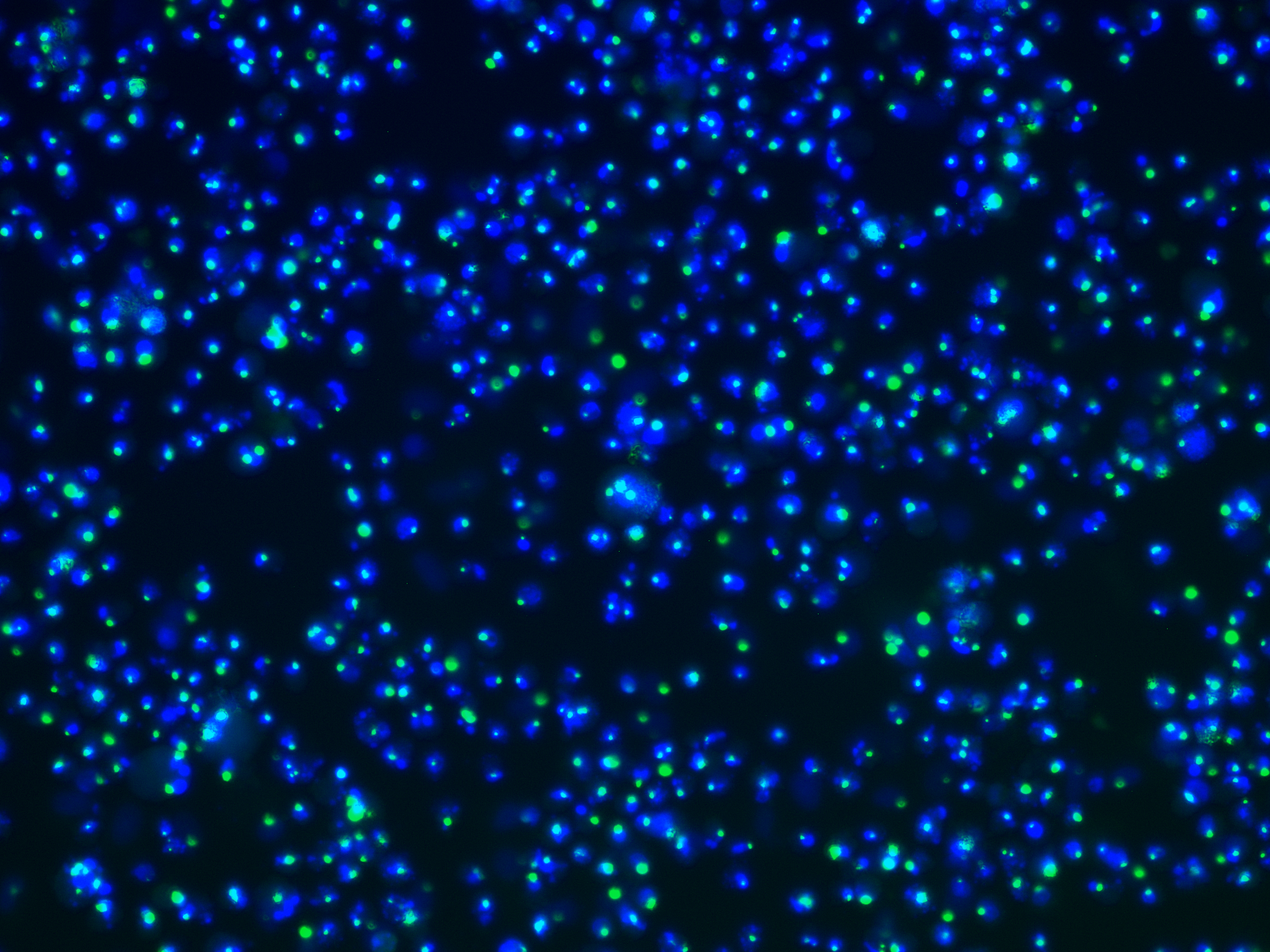

Supplement: Supplementary file 5 — Source data Fig. 3 [file 44321_2025_197_MOESM5_ESM.zip › Figure 3/3E/Anisomycin/Anisomycin - RGB.tif]

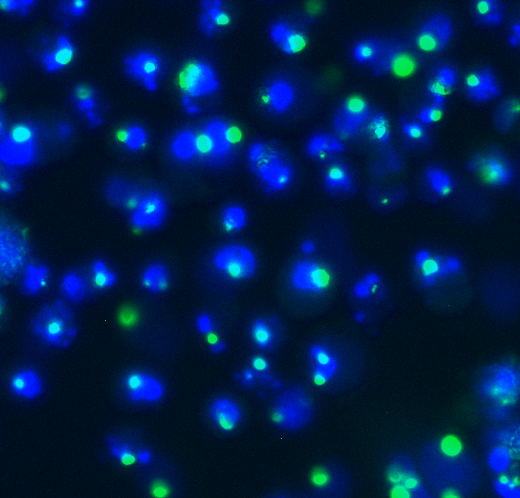

Supplement: Supplementary file 5 — Source data Fig. 3 [file 44321_2025_197_MOESM5_ESM.zip › Figure 3/3E/Anisomycin/Anisomycin - RGB selected area.tif]

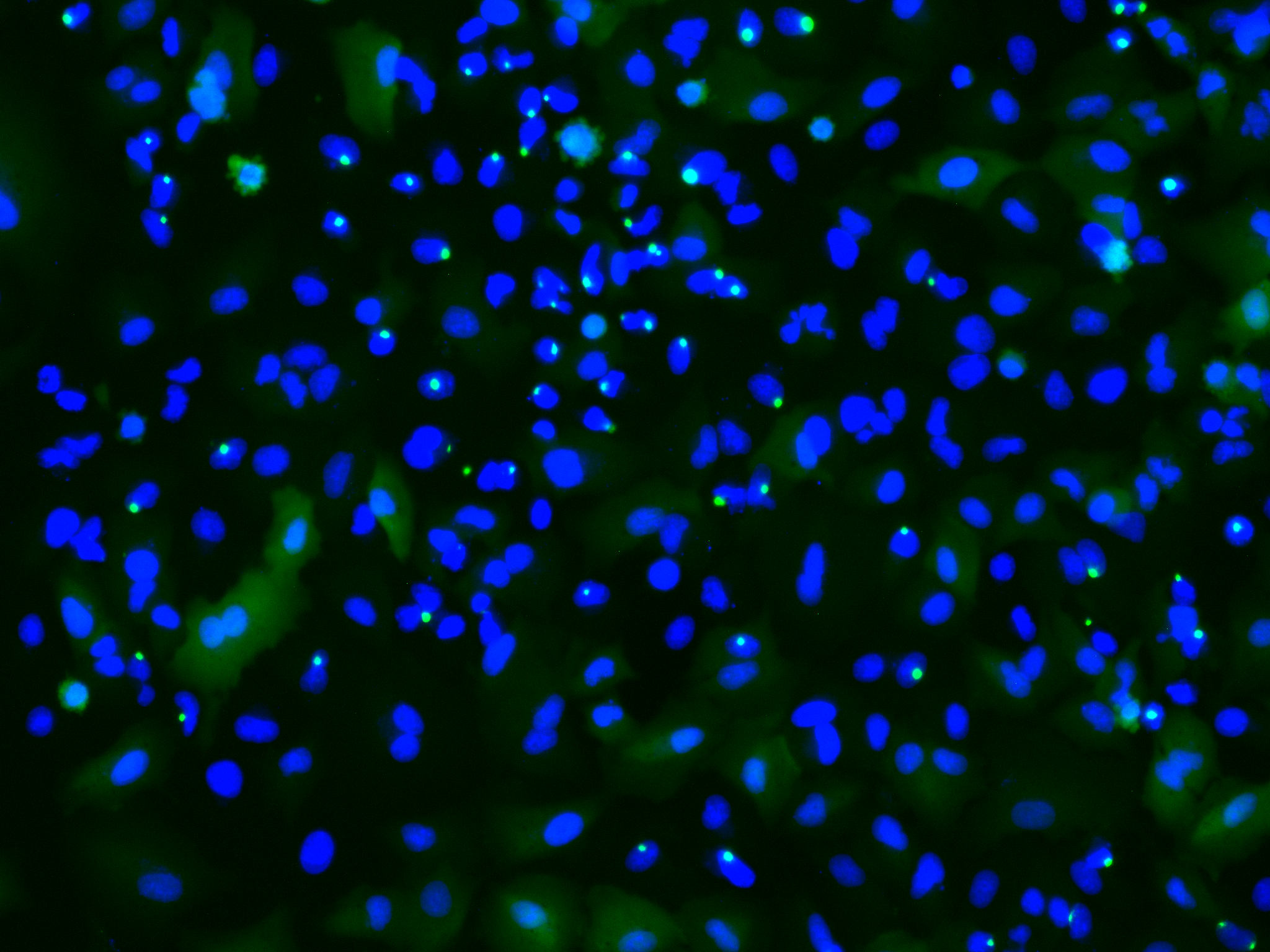

Supplement: Supplementary file 5 — Source data Fig. 3 [file 44321_2025_197_MOESM5_ESM.zip › Figure 3/3E/Portimine A/Portimine A - RGB.tif]

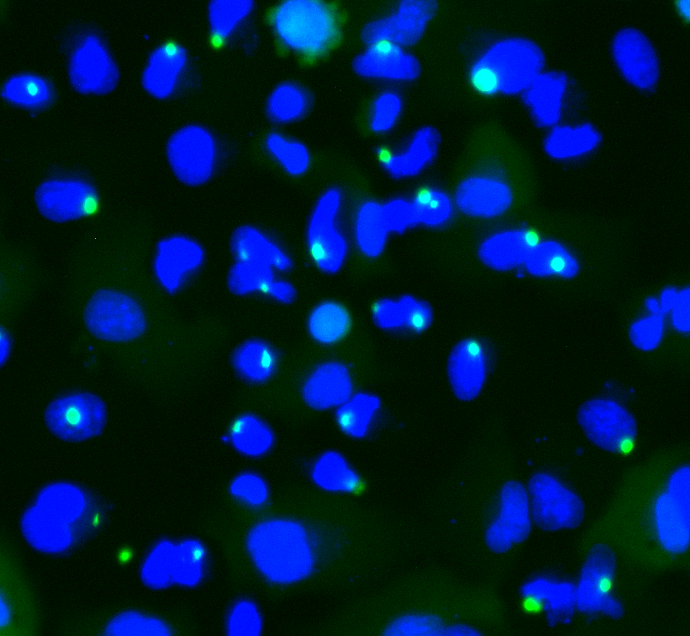

Supplement: Supplementary file 5 — Source data Fig. 3 [file 44321_2025_197_MOESM5_ESM.zip › Figure 3/3E/Portimine A/Portimine A - RGB selected area.tif]

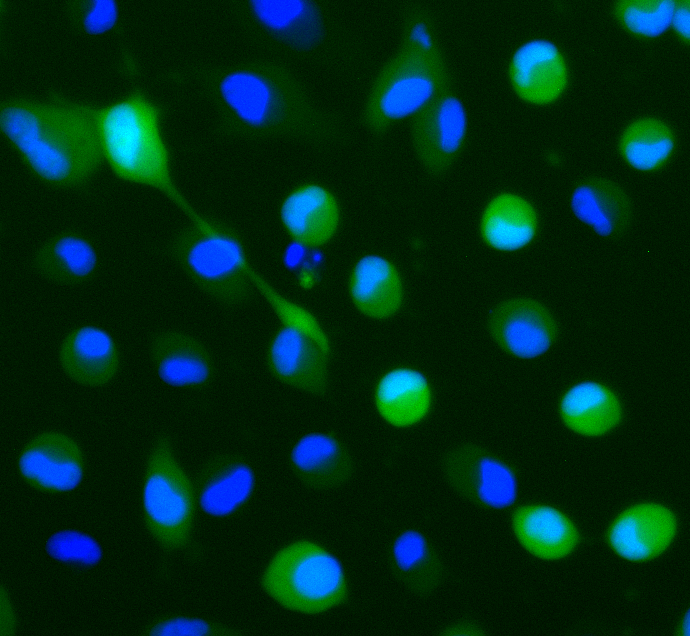

Supplement: Supplementary file 5 — Source data Fig. 3 [file 44321_2025_197_MOESM5_ESM.zip › Figure 3/3G/Mock NLRP1 WT/Mock - RGB selected area.tif]

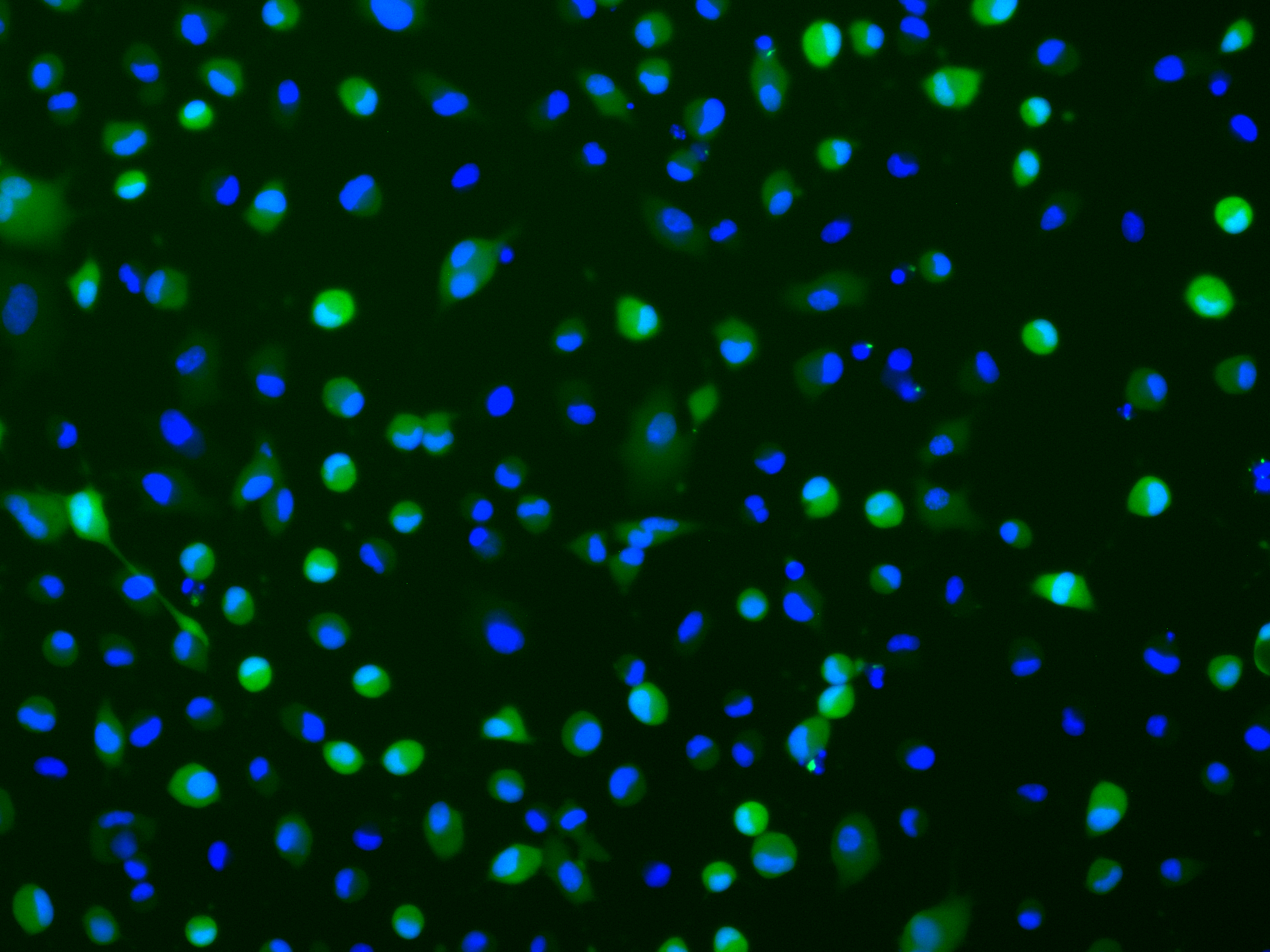

Supplement: Supplementary file 5 — Source data Fig. 3 [file 44321_2025_197_MOESM5_ESM.zip › Figure 3/3G/Mock NLRP1 WT/Mock - RGB.tif]

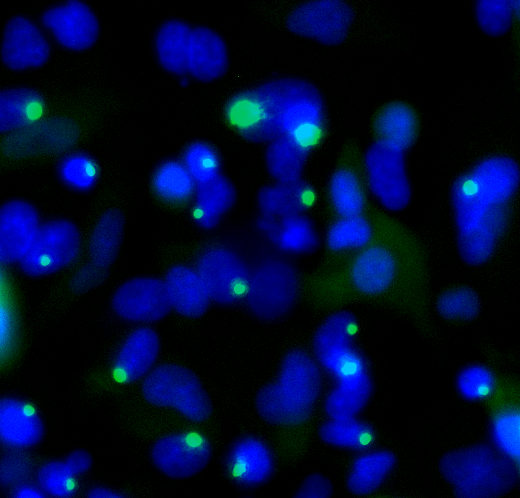

Supplement: Supplementary file 5 — Source data Fig. 3 [file 44321_2025_197_MOESM5_ESM.zip › Figure 3/3G/VbP NLRP1 S107F/VbP NLRP1S107F - RGB selected area.tif]

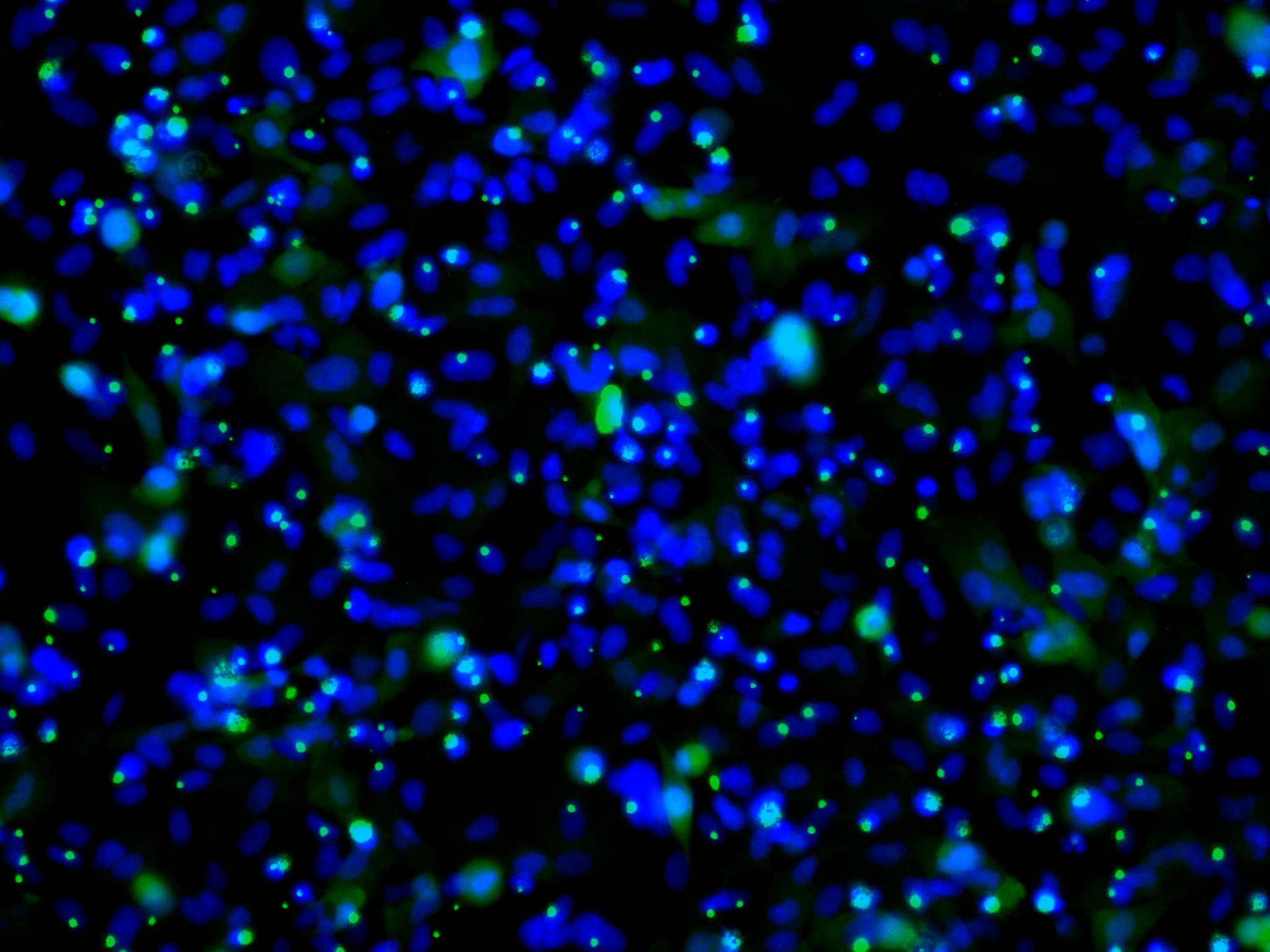

Supplement: Supplementary file 5 — Source data Fig. 3 [file 44321_2025_197_MOESM5_ESM.zip › Figure 3/3G/VbP NLRP1 S107F/VbP NLRP1S107F - RGB.tif]

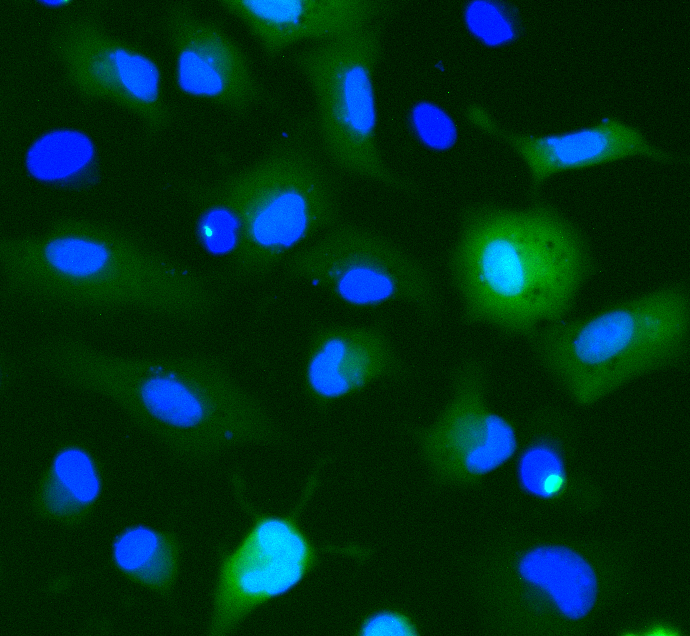

Supplement: Supplementary file 5 — Source data Fig. 3 [file 44321_2025_197_MOESM5_ESM.zip › Figure 3/3G/Mock NLRP1 Del/Mock NLRP1 Del - RGB selected area.tif]

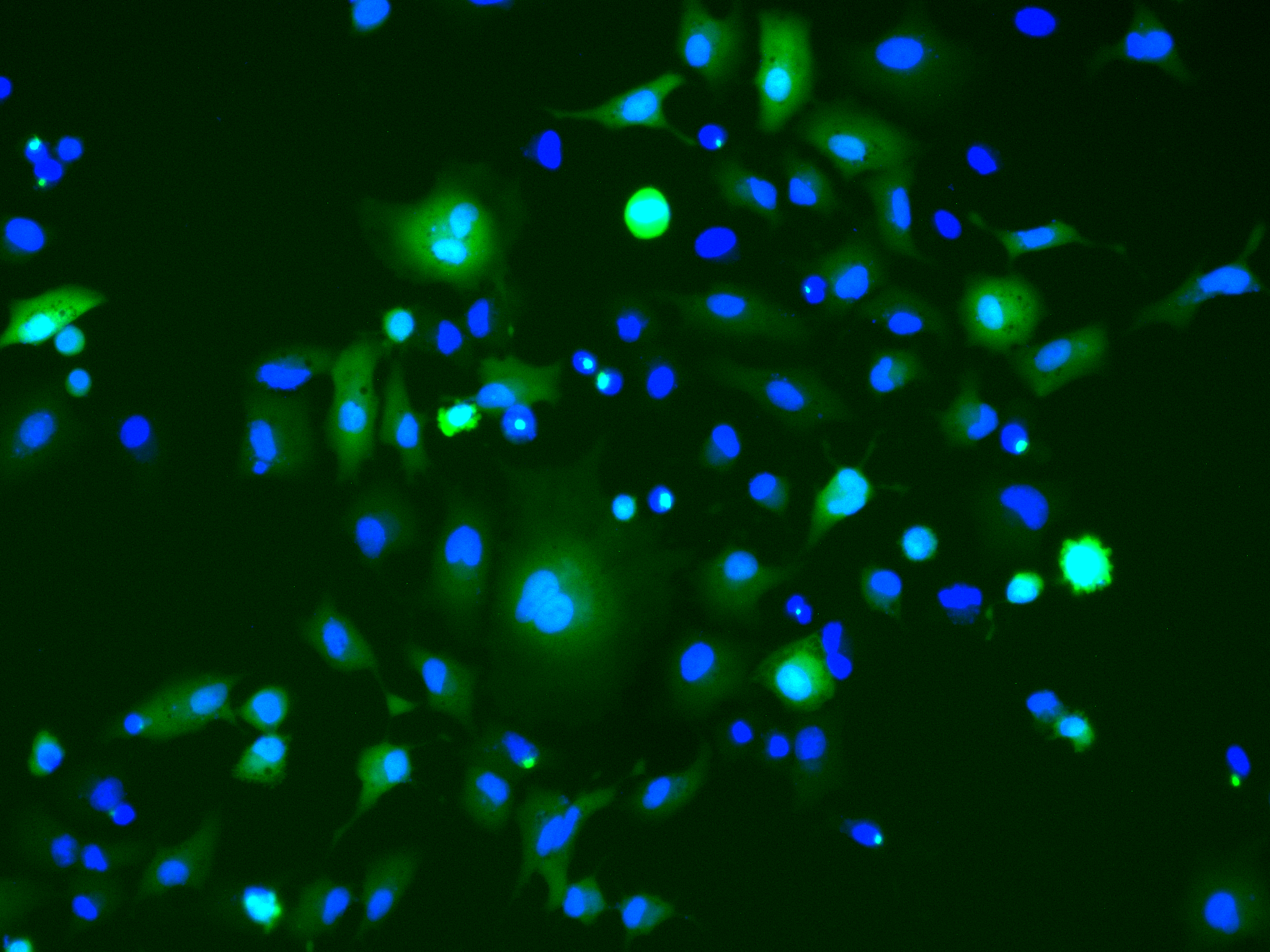

Supplement: Supplementary file 5 — Source data Fig. 3 [file 44321_2025_197_MOESM5_ESM.zip › Figure 3/3G/Mock NLRP1 Del/Mock NLRP1 Del - RGB.tif]

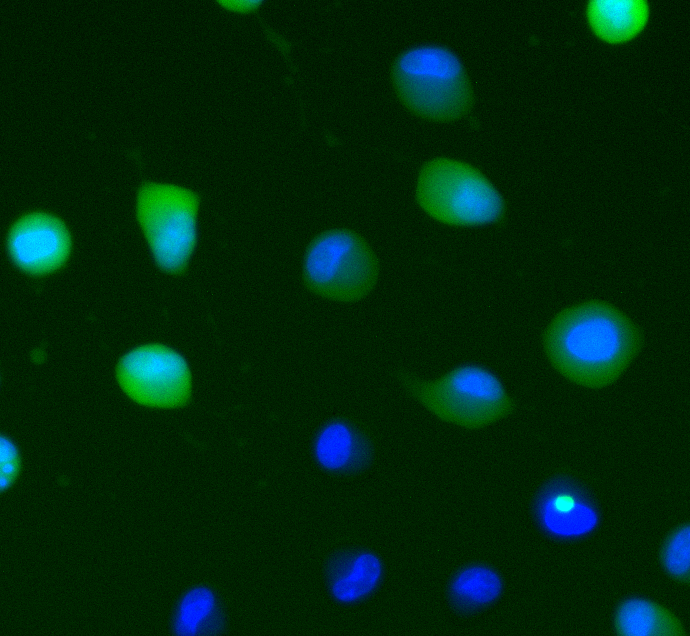

Supplement: Supplementary file 5 — Source data Fig. 3 [file 44321_2025_197_MOESM5_ESM.zip › Figure 3/3G/Mock NLRP1 S107F/Mock Del - RGB.tif]

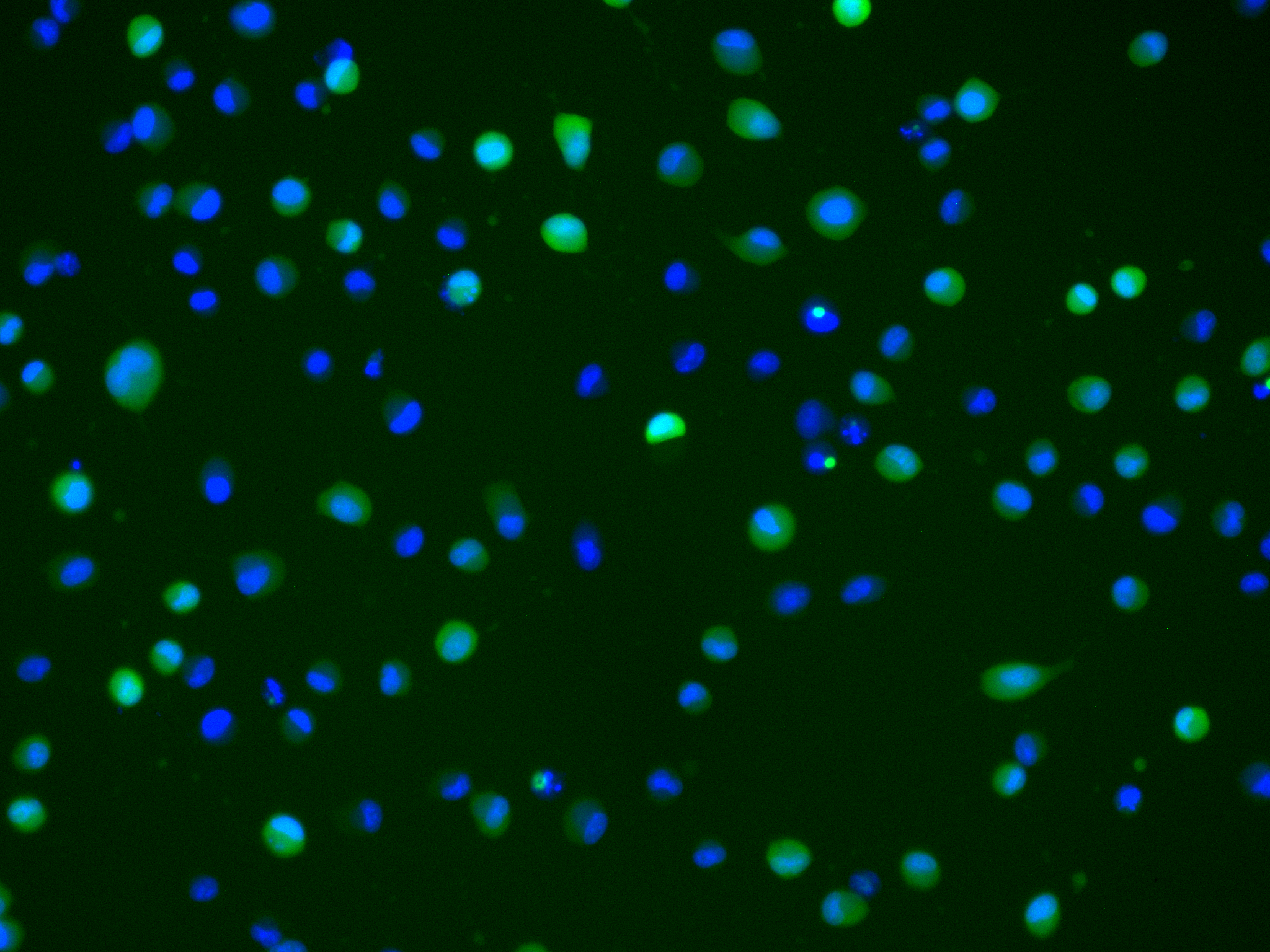

Supplement: Supplementary file 5 — Source data Fig. 3 [file 44321_2025_197_MOESM5_ESM.zip › Figure 3/3G/Mock NLRP1 S107F/Mock Del - RGB selected area.tif]

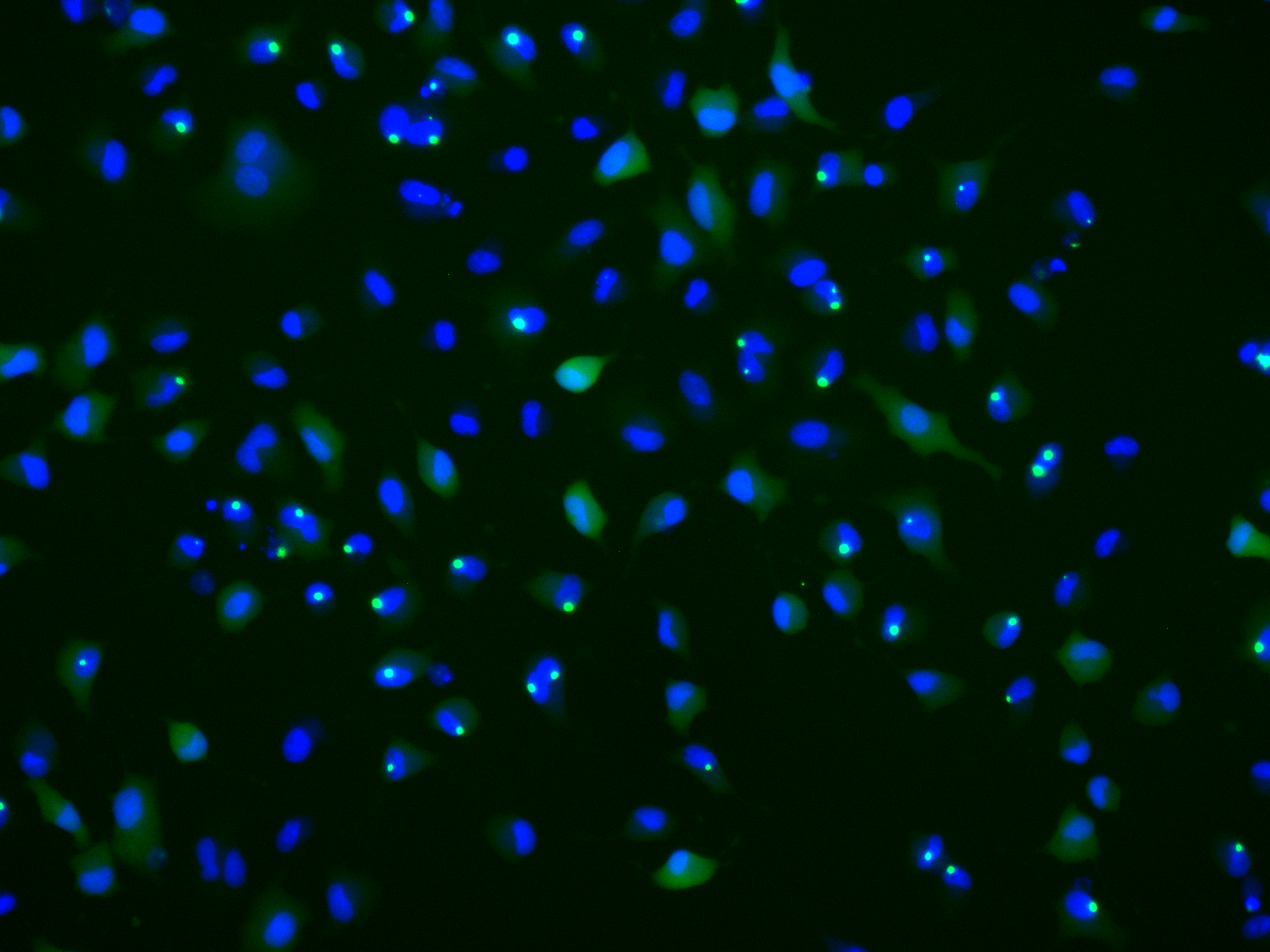

Supplement: Supplementary file 5 — Source data Fig. 3 [file 44321_2025_197_MOESM5_ESM.zip › Figure 3/3G/Portimine A NLRP1 WT/Portimine A - RGB.tif]

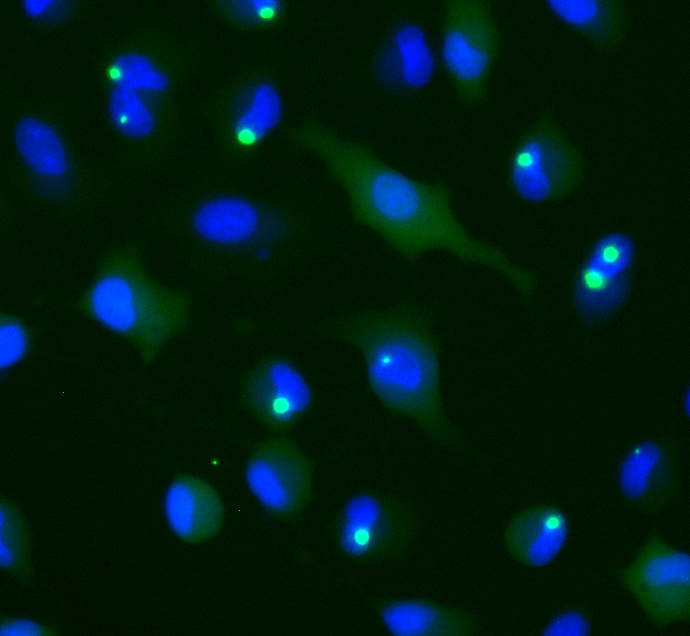

Supplement: Supplementary file 5 — Source data Fig. 3 [file 44321_2025_197_MOESM5_ESM.zip › Figure 3/3G/Portimine A NLRP1 WT/Portimine A - RGB selected area.tif]

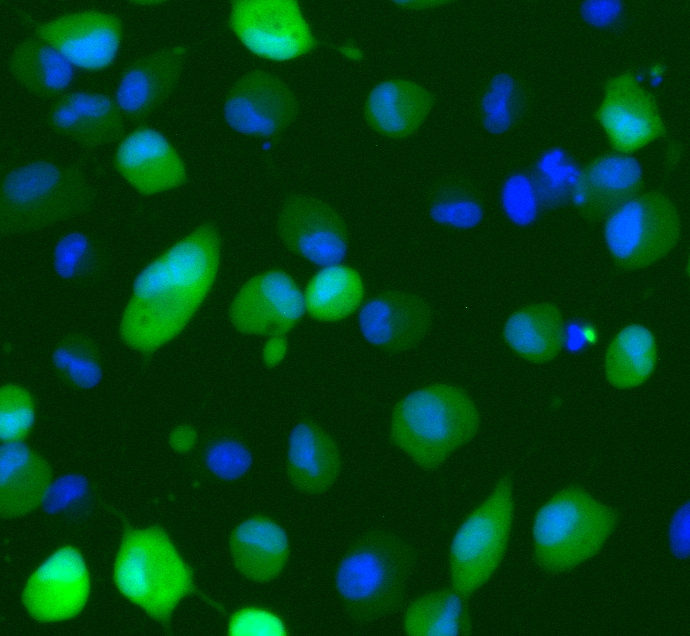

Supplement: Supplementary file 5 — Source data Fig. 3 [file 44321_2025_197_MOESM5_ESM.zip › Figure 3/3G/Portimine A NLRP1 S107F/Portimine A NLRP1 S107F - RGB selected area.tif]

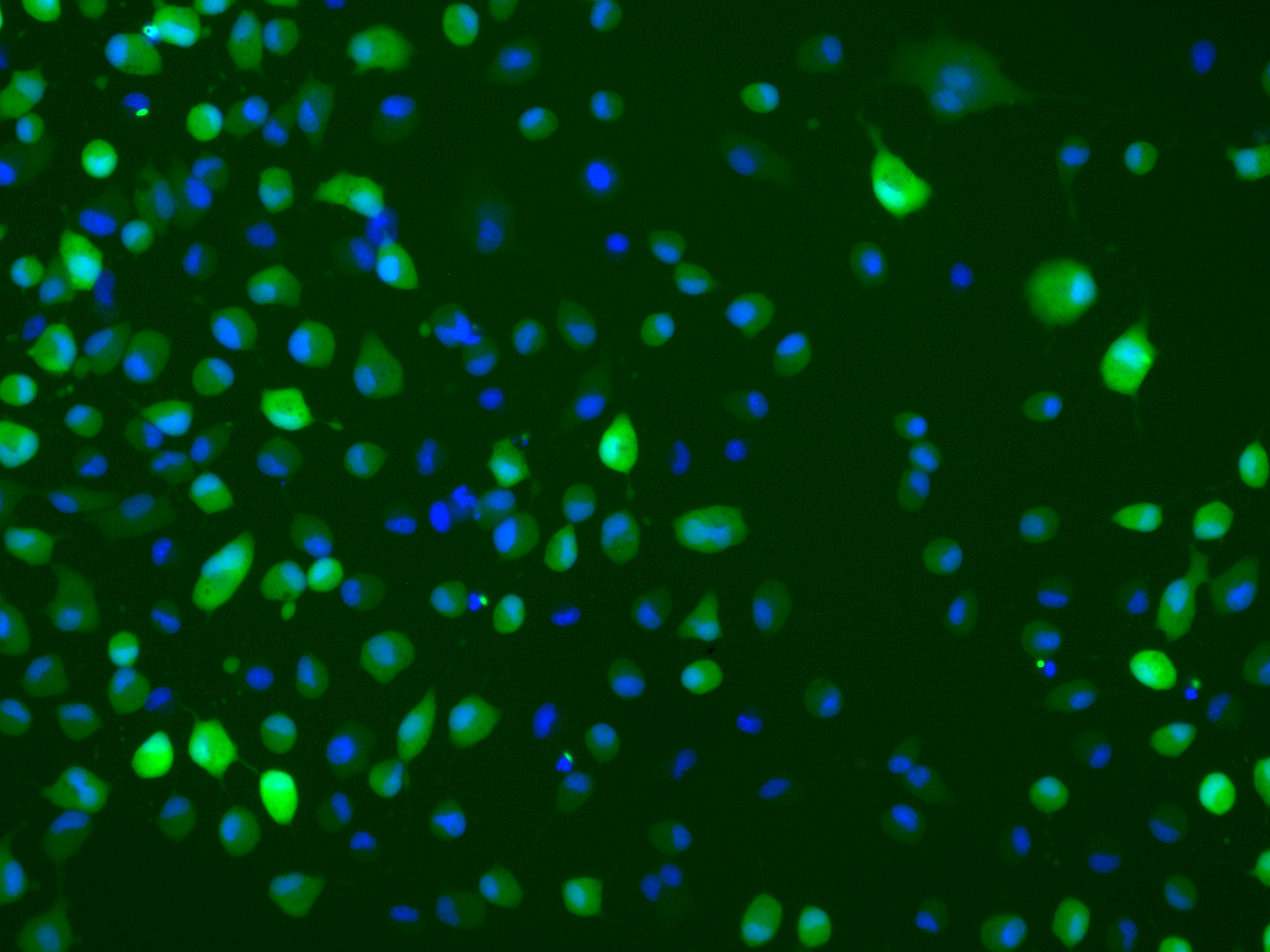

Supplement: Supplementary file 5 — Source data Fig. 3 [file 44321_2025_197_MOESM5_ESM.zip › Figure 3/3G/Portimine A NLRP1 S107F/Portimine A NLRP1 S107F - RGB.tif]

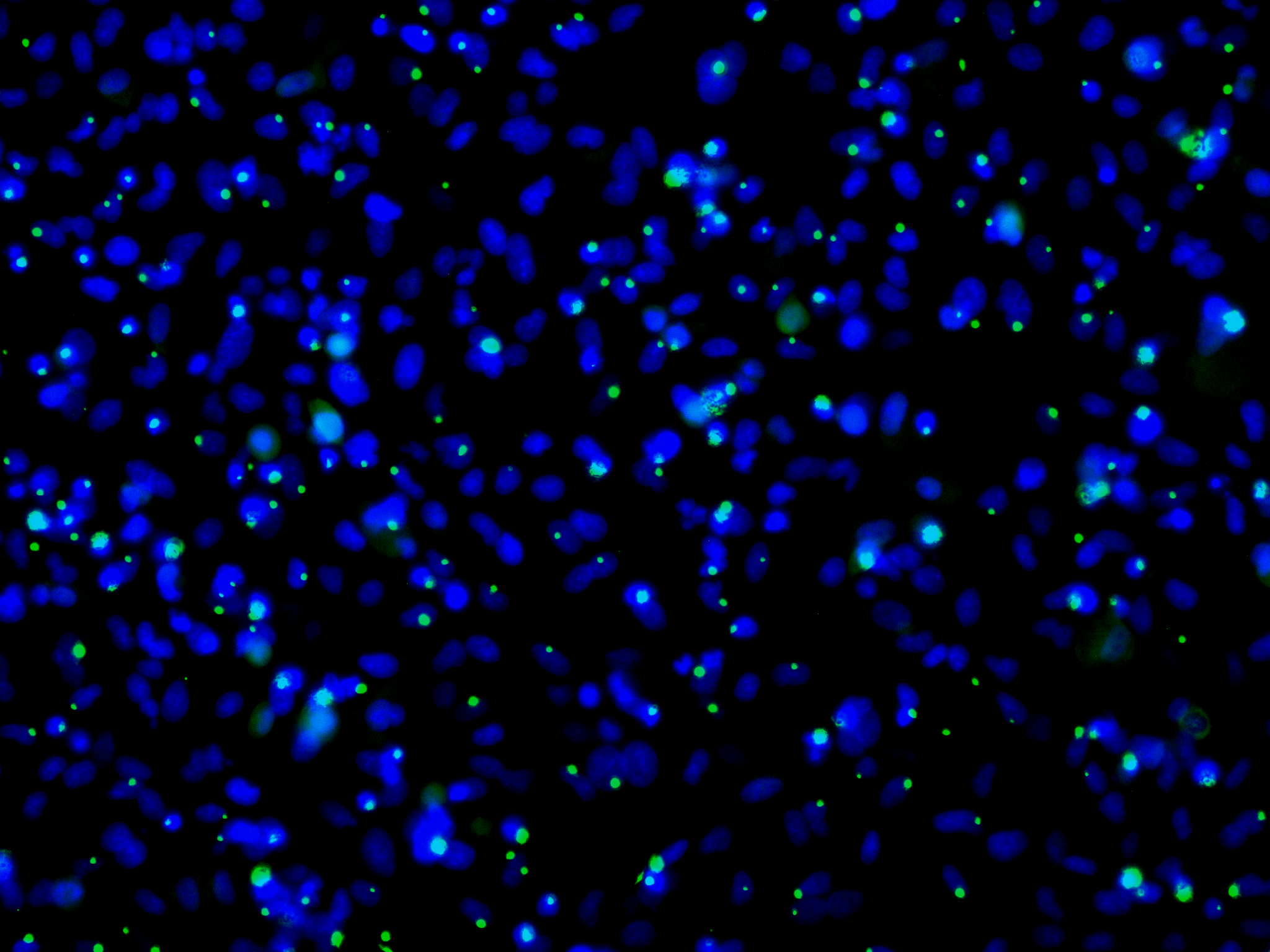

Supplement: Supplementary file 5 — Source data Fig. 3 [file 44321_2025_197_MOESM5_ESM.zip › Figure 3/3G/VbP NLRP1 WT/VbP - RGB.tif]

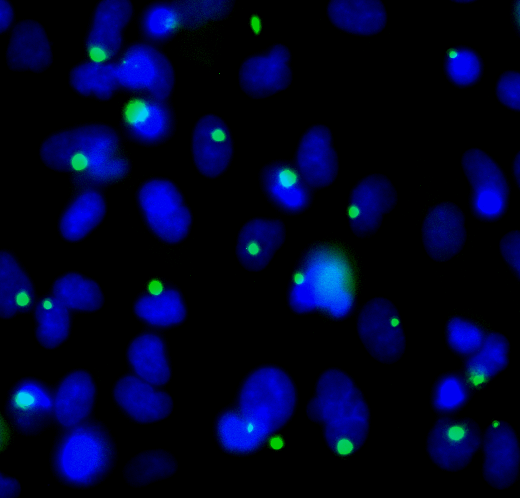

Supplement: Supplementary file 5 — Source data Fig. 3 [file 44321_2025_197_MOESM5_ESM.zip › Figure 3/3G/VbP NLRP1 WT/VbP - RGB selected area.tif]

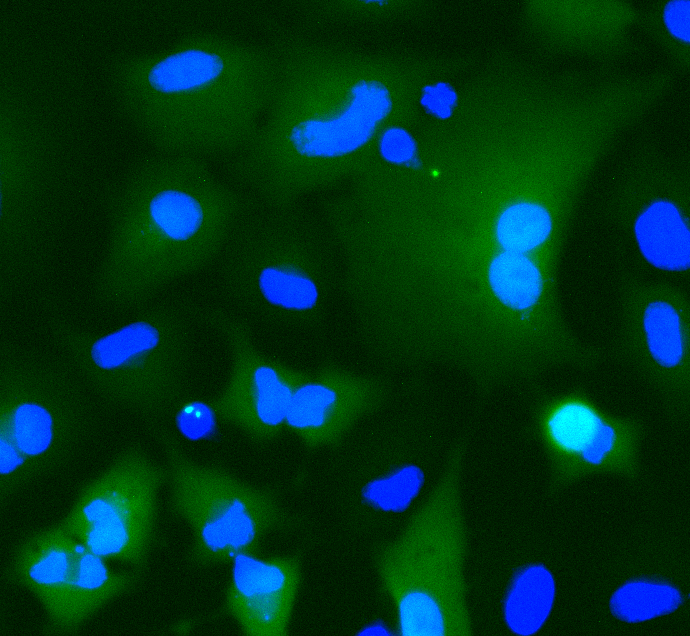

Supplement: Supplementary file 5 — Source data Fig. 3 [file 44321_2025_197_MOESM5_ESM.zip › Figure 3/3G/Portimine A NLRP1 Del/Portimine A NLRP1 Del - RGB Selected area.tif]

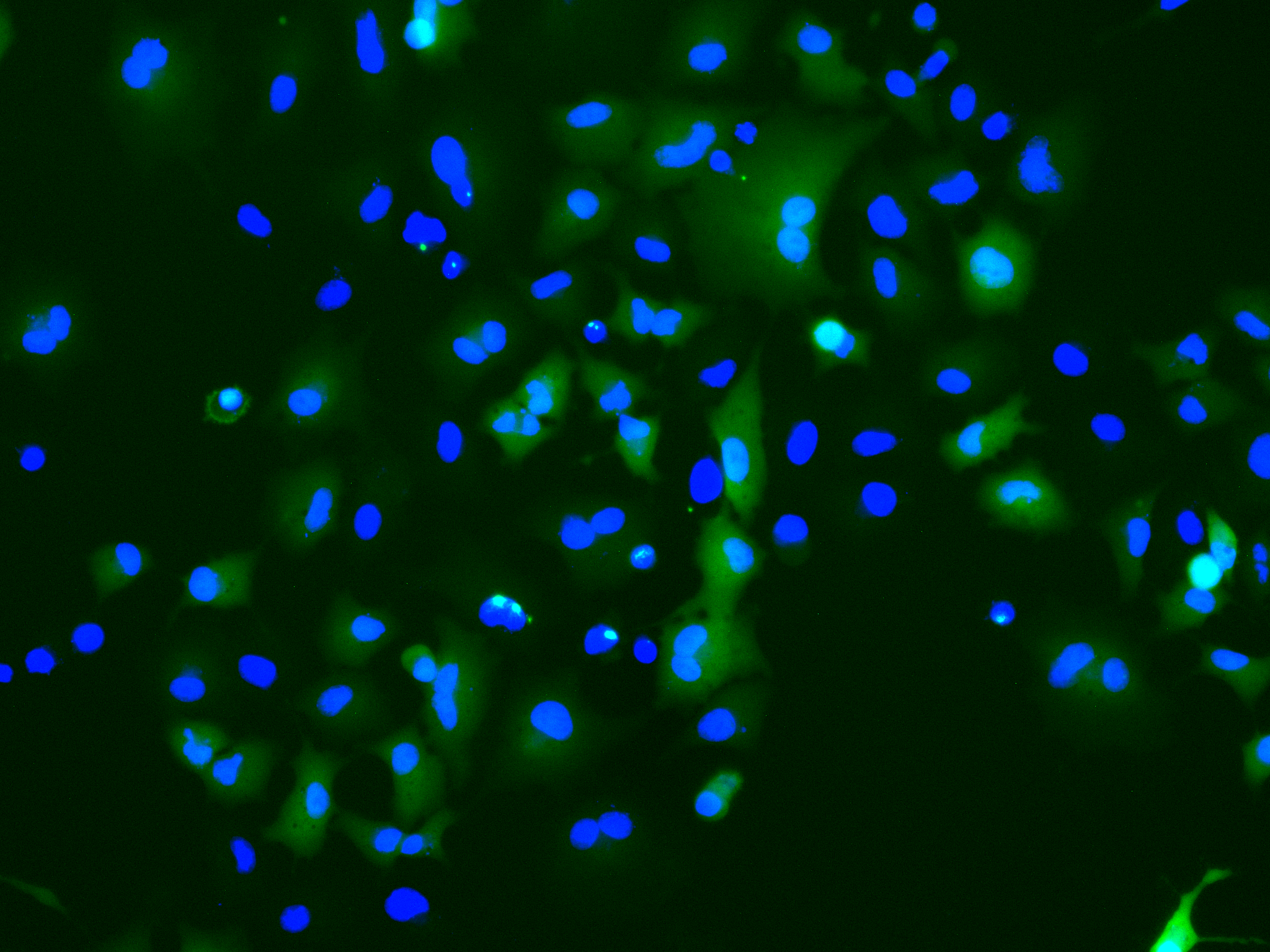

Supplement: Supplementary file 5 — Source data Fig. 3 [file 44321_2025_197_MOESM5_ESM.zip › Figure 3/3G/Portimine A NLRP1 Del/Portimine A NLRP1 Del - RGB.tif]

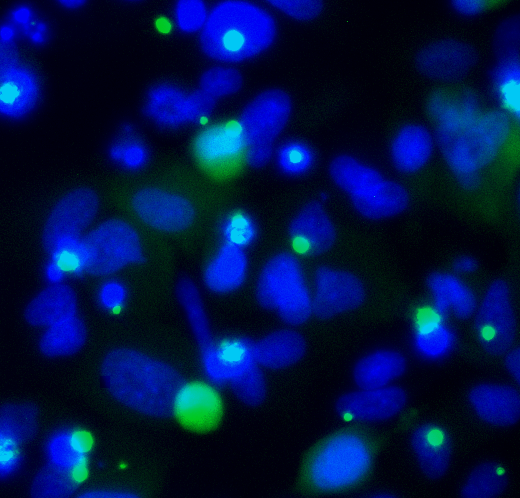

Supplement: Supplementary file 5 — Source data Fig. 3 [file 44321_2025_197_MOESM5_ESM.zip › Figure 3/3G/VbP NLRP1 Del/VbP NLRP1 Del - RGB selected area.tif]

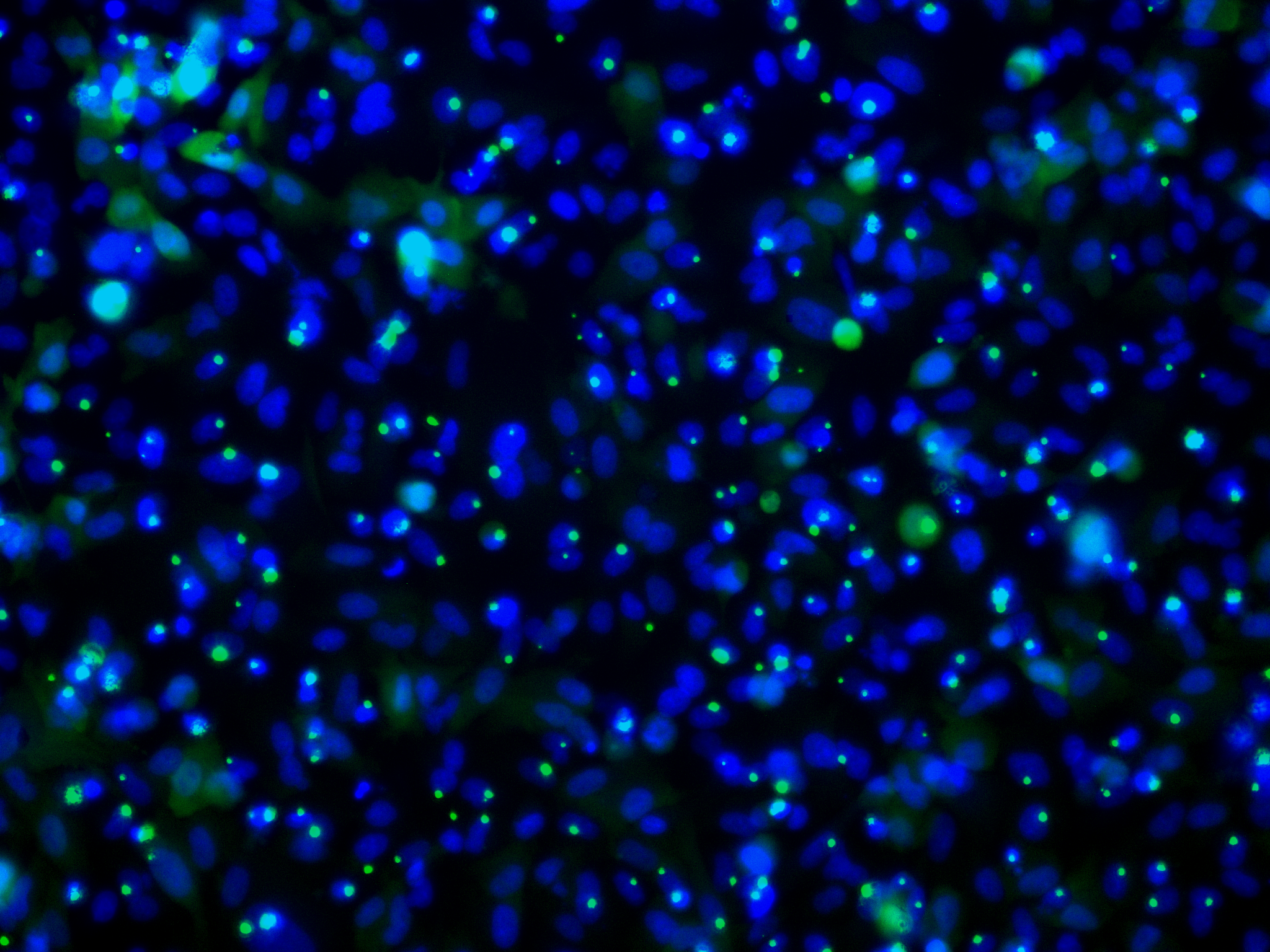

Supplement: Supplementary file 5 — Source data Fig. 3 [file 44321_2025_197_MOESM5_ESM.zip › Figure 3/3G/VbP NLRP1 Del/VbP NLRP1 Del - RGB.tif]

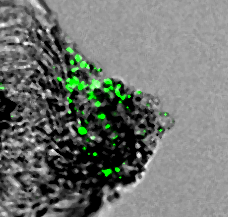

Supplement: Supplementary file 6 — Source data Fig. 4 [file 44321_2025_197_MOESM6_ESM.zip › Figure 4/4E/Portimine A RGB selected area.tif]

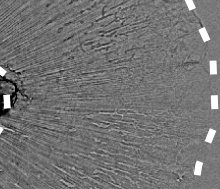

Supplement: Supplementary file 6 — Source data Fig. 4 [file 44321_2025_197_MOESM6_ESM.zip › Figure 4/4E/Untreated RGB selected area.tif]

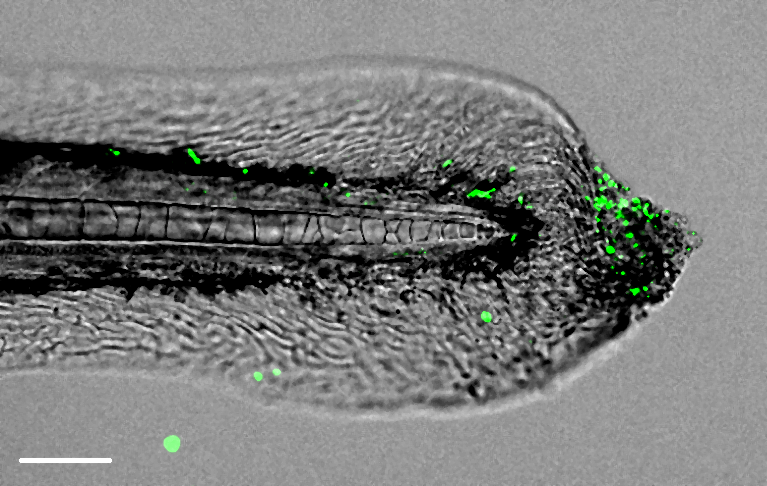

Supplement: Supplementary file 6 — Source data Fig. 4 [file 44321_2025_197_MOESM6_ESM.zip › Figure 4/4E/Portimine A RGB.tif]

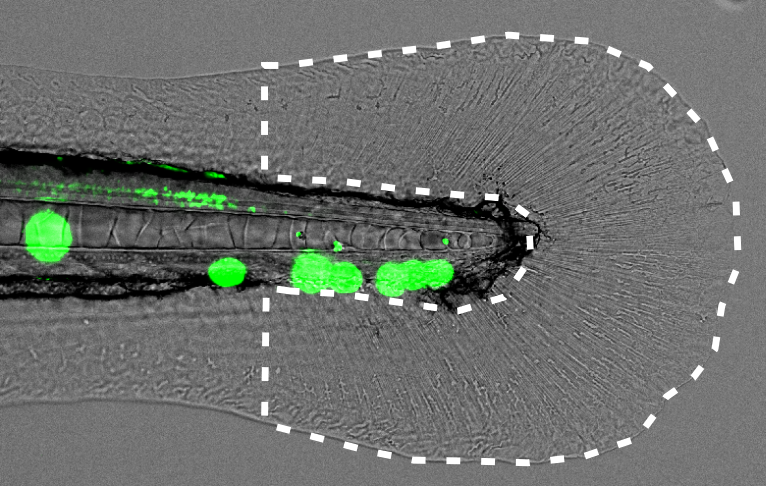

Supplement: Supplementary file 6 — Source data Fig. 4 [file 44321_2025_197_MOESM6_ESM.zip › Figure 4/4E/Untreated RGB.tif]

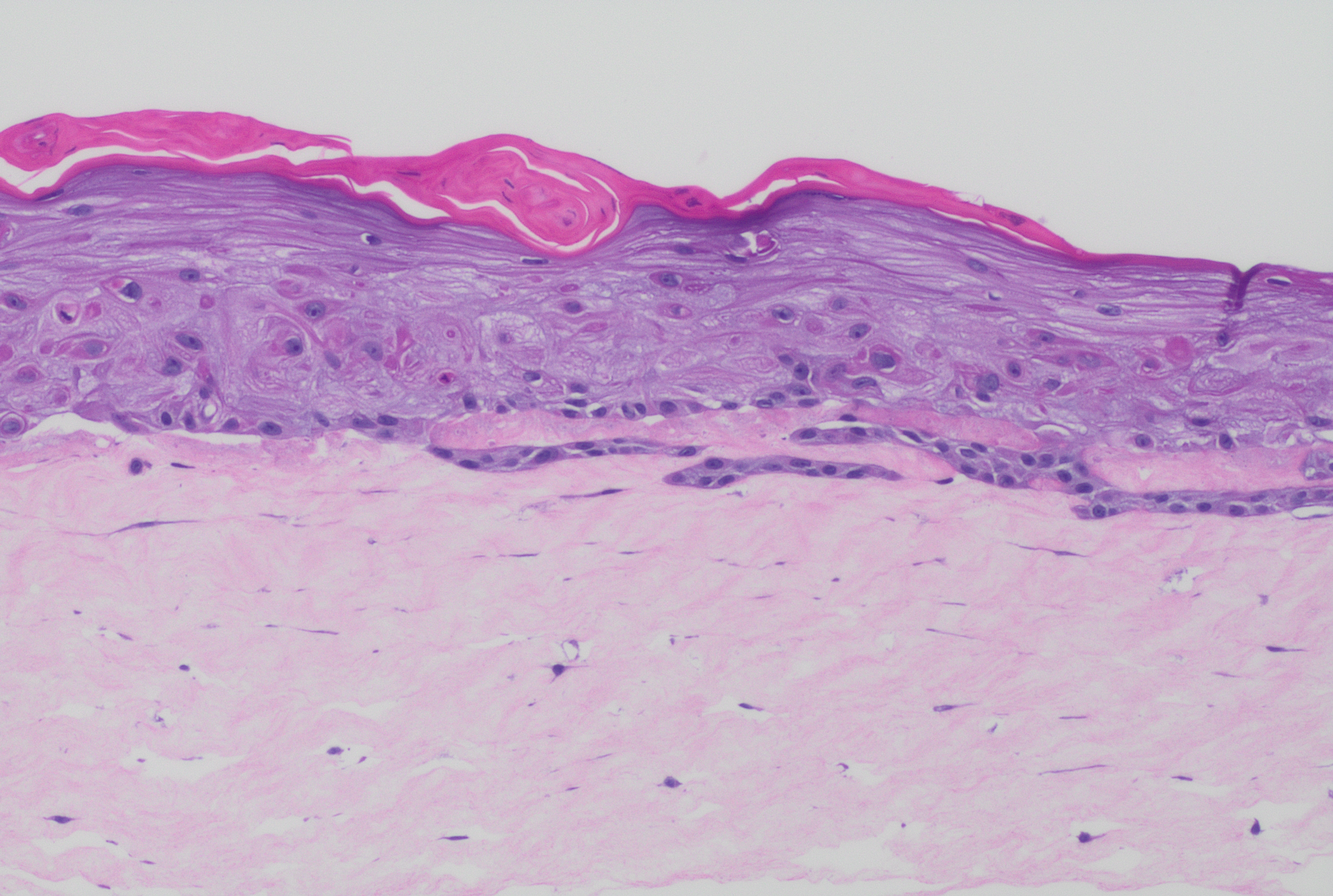

Supplement: Supplementary file 6 — Source data Fig. 4 [file 44321_2025_197_MOESM6_ESM.zip › Figure 4/4B/Portimine A ZAK-20-6.tif]

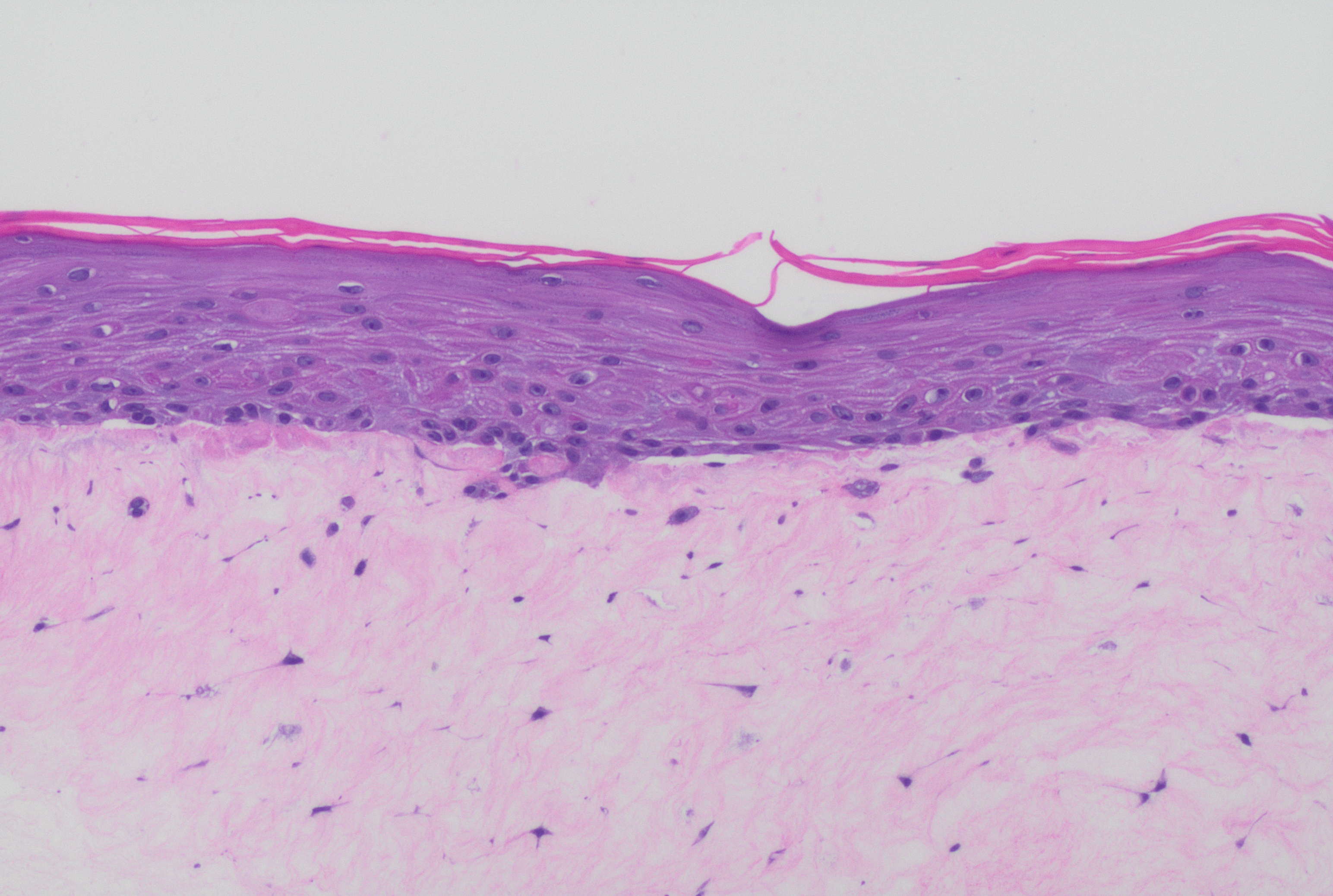

Supplement: Supplementary file 6 — Source data Fig. 4 [file 44321_2025_197_MOESM6_ESM.zip › Figure 4/4B/Mock ZAKN-0-3.tif]

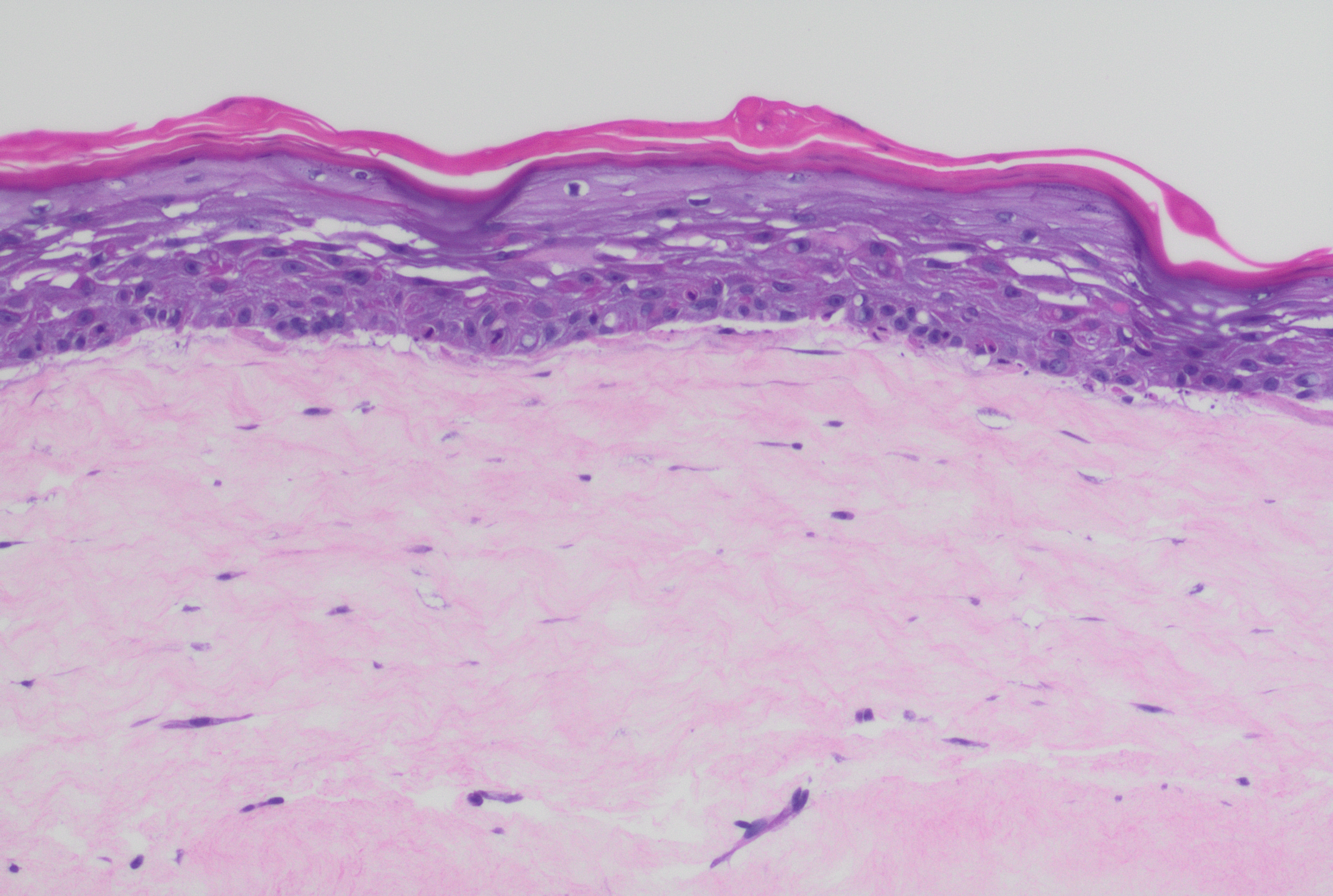

Supplement: Supplementary file 6 — Source data Fig. 4 [file 44321_2025_197_MOESM6_ESM.zip › Figure 4/4B/Portimine A WT-20-6.tif]

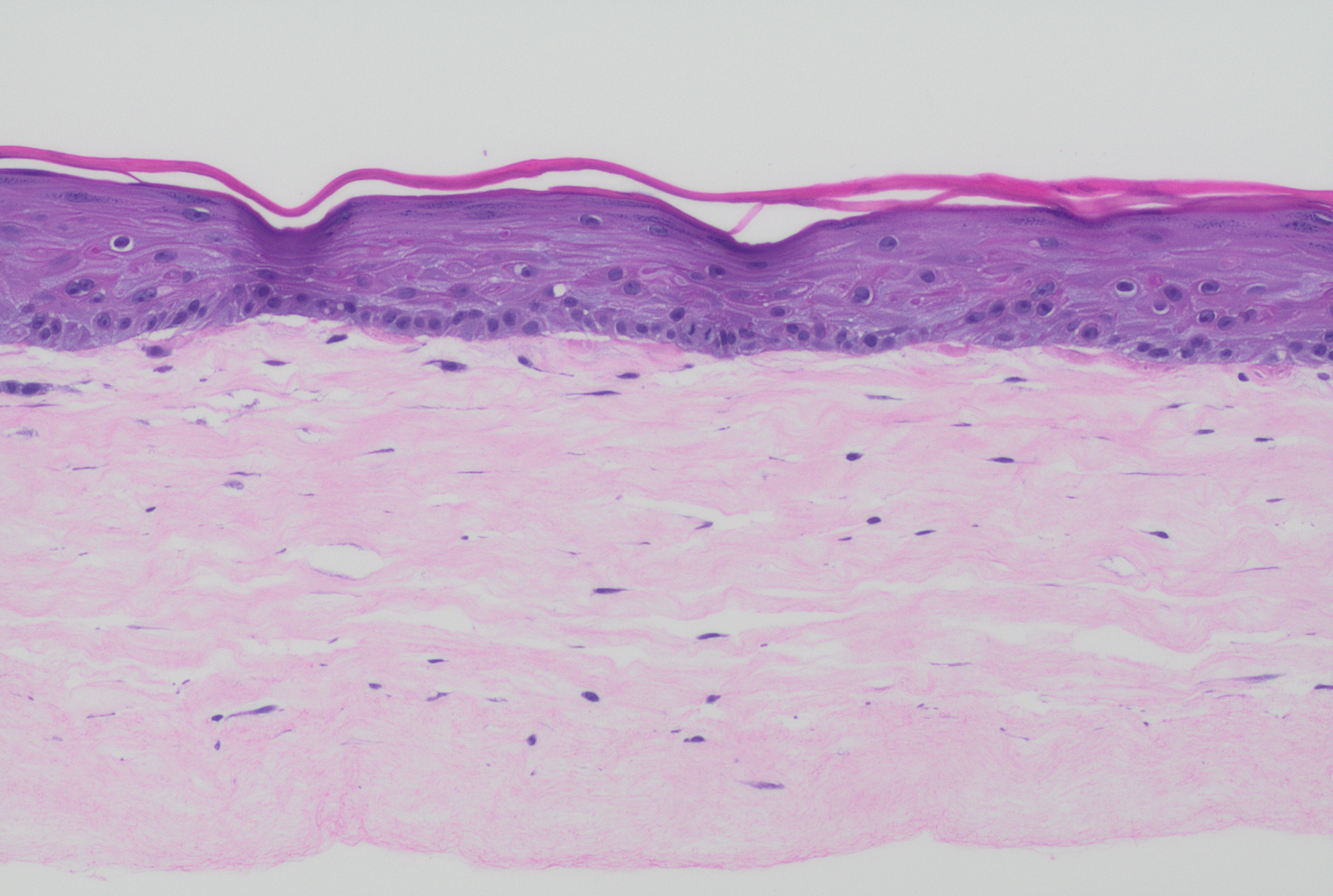

Supplement: Supplementary file 6 — Source data Fig. 4 [file 44321_2025_197_MOESM6_ESM.zip › Figure 4/4B/Mock WT - N-0-1.tif]

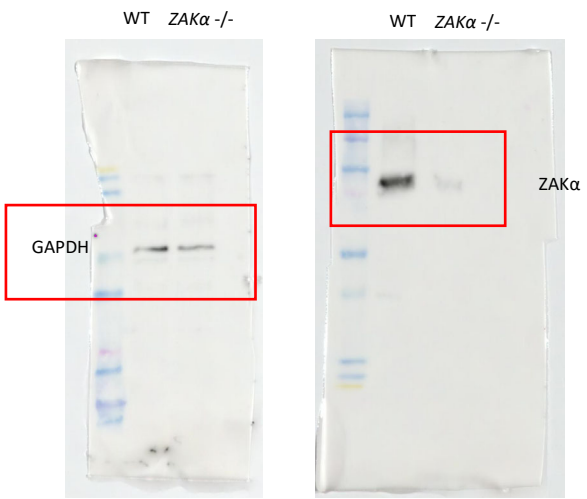

Supplement: Supplementary file 6 — Source data Fig. 4 [file 44321_2025_197_MOESM6_ESM.zip › Figure 4/4A/Immunoblotting membranes.pdf]

Portimine-A

---

WT

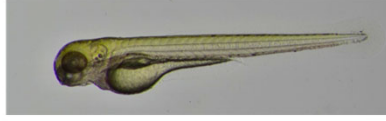

WT

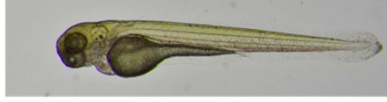

*Nlrp1*<sup>-/-</sup>

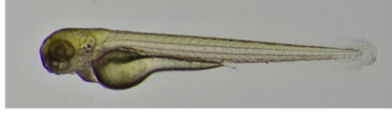

*ZAKα*<sup>-/-</sup>

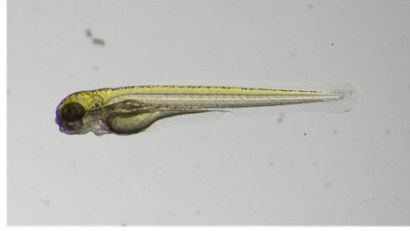

Supplement: Supplementary file 6 — Source data Fig. 4 [file 44321_2025_197_MOESM6_ESM.zip › Figure 4/4F/Raw images zebrafish 4F.pdf]

Neutrophils (LysC:GFP)

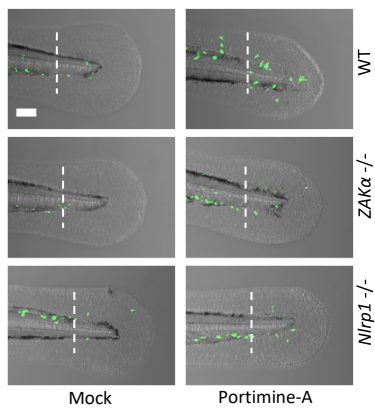

Supplement: Supplementary file 6 — Source data Fig. 4 [file 44321_2025_197_MOESM6_ESM.zip › Figure 4/4G/Neutrophil images 4G.pdf]
