# Supplementary material for: Portimine A toxin causes skin inflammation through ZAKα-dependent NLRP1 inflammasome activation
Source: EMBO Mol Med. 2025 Feb 13;17(3):535–62. doi: 10.1038/s44321-025-00197-4 (PMC11903881; doi:10.1038/s44321-025-00197-4)
Supplement: Supplementary file 7 — Expanded View Figures [file 44321_2025_197_MOESM7_ESM.pdf]

# Expanded View Figures

**Figure EV1. (refers to Fig. 1): *Vulcanodinium rugosum*-produced Portimine A triggers human skin epithelial cell necrosis and IL-1 cytokine release.**

(**A**) Amplification results of qPCR assay including derivative melting curves plot and amplification curves plot. The qPCR analysis was performed in duplicate. The melting temperature (Tm) was calculated to be 80.3 °C for the strain and sample and the cycle threshold was of 24 and 31 cycles, respectively. (**B**) Cytokine analysis 24 h after exposure of primary human keratinocytes (pHEKs) to purified extracts (Sample II isolated in Fig. 1C, dilution 1/20,000 from the isolated fraction). Representative experiment of three independent experiments. (**C**) Cytotoxicity of Pinnatoxin H and -G, Portimine A and -B pHEK. 24 h treatment, $n = 3$, mean ± SEM. (**D**) Cytotoxicity of extracts from *V. rugosum* cultures (IFR-VRU-01) and biomass sampled in Senegal (2020) on pHEK, expressed as PortimineA equivalent concentrations (determined by LC-MS/MS), 24 h treatment, $n = 3$, mean ± SEM. (**E**) Cell lysis (LDH) evaluation in pHEKs after 30 h exposure to Sample II, pure Portimine A (1,7 ng/mL, calculated from Sample II $IC_{50}$ in **D**), Portimine B (1,7 ng/mL, calculated from Sample II $IC_{50}$ in **D**), pure 400 ng/mL Pinnatoxin H and -G or combinations of all those toxins by always keeping a final concentration of 1.7 nM of Portimine A or Poritmine B in the different mixtures generated. ***$P \leq 0.0001$, two-way ANOVA with multiple comparisons. Values are expressed as mean ± SEM. Graphs show one experiment performed in triplicates at least three times. (**F**) Plasma membrane permeabilization (SYTOX Green incorporation, 9 h) in pHEKs after exposure to Sample II (1/20,000), Portimine A (4 ng/mL), Portimine B (400 ng/mL) or Pinnatoxin G/H (40 ng/mL). ***$P \leq 0.0001$, two-way ANOVA with multiple comparisons. Values are expressed as mean ± SEM. Graphs show one experiment performed in triplicates at least three times. (**G**) SYTOX Green incorporation in pHEKs, primary human endothelial, nasal, monocytes, lymphocytes or neutrophils cells 6 h after exposure to Portimine A (4 ng/mL) or Portimine B (4 ng/mL). ***$P \leq 0.0001$, two-way ANOVA with multiple comparisons. Values are expressed as mean ± SEM. Graphs show one experiment performed in triplicates at least three times.

 © The Author(s)

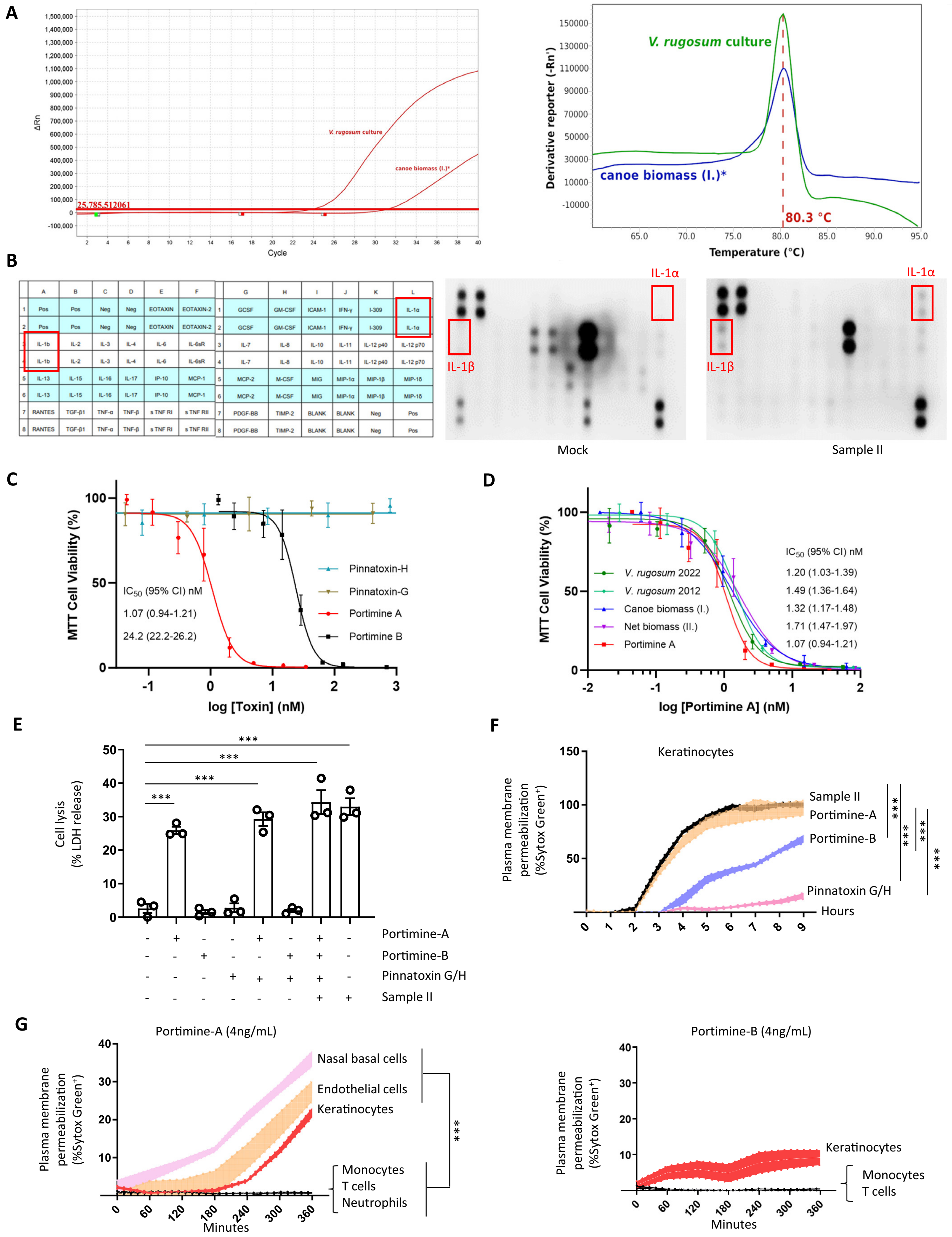
A
ΔRn
Cycle
V. rugosum culture
canoe biomass (I.)*
25,785.512061
Derivative reporter (-Rn')
Temperature (°C)
80.3 °C
B
IL-1α
IL-1β
Mock
Sample II
C
MTT Cell Viability (%)
log [Toxin] (nM)
IC50 (95% CI) nM
1.07 (0.94-1.21)
24.2 (22.2-26.2)
Pinnatoxin-H
Pinnatoxin-G
Portimine A
Portimine B
D
MTT Cell Viability (%)
log [Portimine A] (nM)
IC50 (95% CI) nM
V. rugosum 2022 1.20 (1.03-1.39)
V. rugosum 2012 1.49 (1.36-1.64)
Canoe biomass (I.) 1.32 (1.17-1.48)
Net biomass (II.) 1.71 (1.47-1.97)
Portimine A 1.07 (0.94-1.21)
E
Cell lysis (% LDH release)
***
Portimine-A
Portimine-B
Pinnatoxin G/H
Sample II
F
Keratinocytes
Plasma membrane permeabilization (%Sytox Green+)
Sample II
Portimine-A
Portimine-B
Pinnatoxin G/H
Hours
***
G
Portimine-A (4ng/mL)
Nasal basal cells
Endothelial cells
Keratinocytes
Monocytes
T cells
Neutrophils
Minutes
Portimine-B (4ng/mL)
Keratinocytes
Monocytes
T cells
Minutes

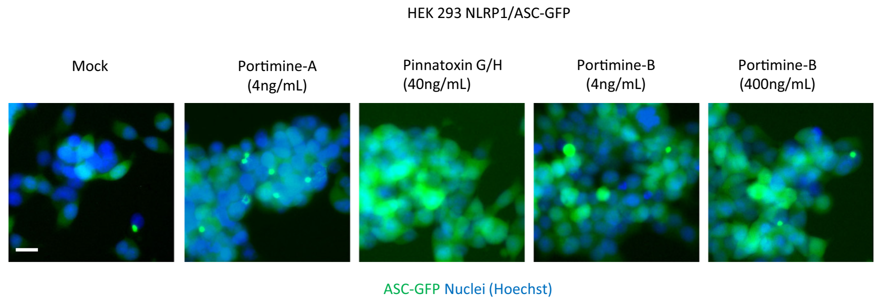


**Figure EV2. (refers to Fig. 2): Portimine A activates the NLRP1 inflammasome in human skin epithelial cells.**

Fluorescence micrographs of ASC-GFP specks in HEK293T cells individually expressing NLRP1 and exposed to 4 ng/mL of Portimine A, 400 ng/mL of Pinnatoxin H/G or to 4 ng/mL or 400 ng/mL of Portimine B for 12 h. ASC-GFP (green) pictures were directly taken after adding Hoechst (nuclei staining). Images shown are from one experiment and are representative of $n = 3$ independent experiments. Scale bar, 10 µm.

 © The Author(s)

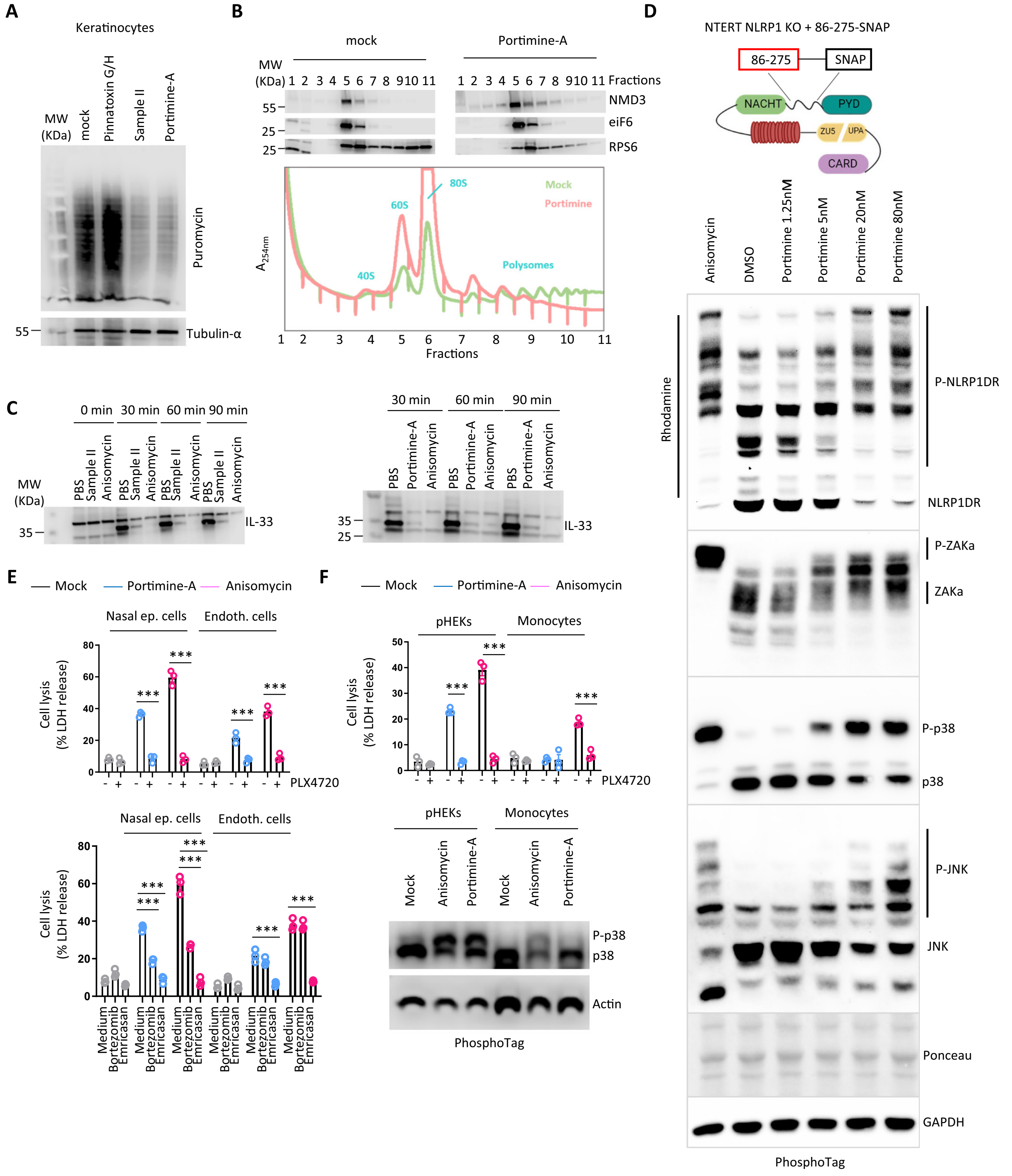
A
Keratinocytes
MW (KDa)
mock
Pinnatoxin G/H
Sample II
Portimine-A
Puromycin
55
Tubulin-α
B
mock
Portimine-A
MW (KDa)
1 2 3 4 5 6 7 8 9 10 11 Fractions
NMD3
eiF6
RPS6
55
25
25
80S
60S
40S
Polysomes
Mock
Portimine
A254nm
1 2 3 4 5 6 7 8 9 10 11
Fractions
C
0 min 30 min 60 min 90 min
PBS
Sample II
Anisomycin
MW (KDa)
35
IL-33
30 min 60 min 90 min
PBS
Portimine-A
Anisomycin
35
25
IL-33
D
NTERT NLRP1 KO + 86-275-SNAP
86-275
SNAP
NACHT
PYD
ZU5
UPA
CARD
Anisomycin
DMSO
Portimine 1.25nM
Portimine 5nM
Portimine 20nM
Portimine 80nM
Rhodamine
P-NLRP1DR
NLRP1DR
P-ZAKa
ZAKa
P-p38
p38
P-JNK
JNK
Ponceau
GAPDH
PhosphoTag
E
Mock Portimine-A Anisomycin
Nasal ep. cells Endoth. cells
Cell lysis (% LDH release)
***
PLX4720
Medium
Bortezomib
Emricasan
F
Mock Portimine-A Anisomycin
pHEKs Monocytes
Cell lysis (% LDH release)
***
PLX4720
pHEKs Monocytes
Mock
Anisomycin
Portimine-A
P-p38
p38
Actin
PhosphoTag

**Figure EV3. (refers to Fig. 3): Portimine-inhibited translation promotes ZAKα-dependent P38 activation and hNLRP1 inflammasome activation in epithelial cells.**

(**A**) Determination of protein synthesis in pHEKs in response to Sample II (1/20000 dilution), Portimine A (4 ng/mL) or Pinnatoxins-H/G (40 ng/mL) by measuring puromycin incorporation after 10 h exposure. Immunoblots show lysates from one experiment performed at least three times. (**B**) Ribosome profiling and ribosomal fraction analysis after exposing HEK293 cells expressing or not NLRP1 to Portimine A (4 ng/mL) for 2 h. Images and Immunoblotting are representatives of one experiment performed at least three times. (**C**) In vitro translation of the reporter plasmid coding for Interleukin 33 (IL-33) by rabbit reticulocyte lysates in the presence/absence of Sample II (1/20,000 dilution), Portimine A (4 ng/mL), Anisomycin (1 µg/mL). Immunoblotting are representatives of one experiment performed at least three times. (**D**) Phosphotag blotting of phosphorylated ZAKα, P38, JNK and NLRP1 disordered Region (DR) in NTERT NLRP1 KO + 86-275-SNAP cells exposed to various amounts of Portimine A or to the known RSR inducer Anisomycin (1 µg/mL) for one hour. Ponceau staining and GAPDH were used as internal protein loading controls. Immunoblots show lysates from one experiment performed at least two times. (**E**, **F**) Phosphotag blotting of phosphorylated P38 and cell lysis (LDH) evaluation in pHEKs, endothelial cells, nasal basal cells and human blood monocytes after 24 h exposure to pure Portimine A (4 ng/mL) or Anismoycin (1 µg/mL and 10 µg/mL in monocytes). When specified the compounds PLX4720 (ZAKα, 10 µM), Emricasan (pan Caspase inhibitor, 5 µM) and bortezomib (proteasome inhibitor, 1 µM) were used. $^{***}P \leq 0.0001$, two-way ANOVA with multiple comparisons. Values are expressed as mean ± SEM. Graphs show one experiment performed in triplicates at least three times.

 © The Author(s)

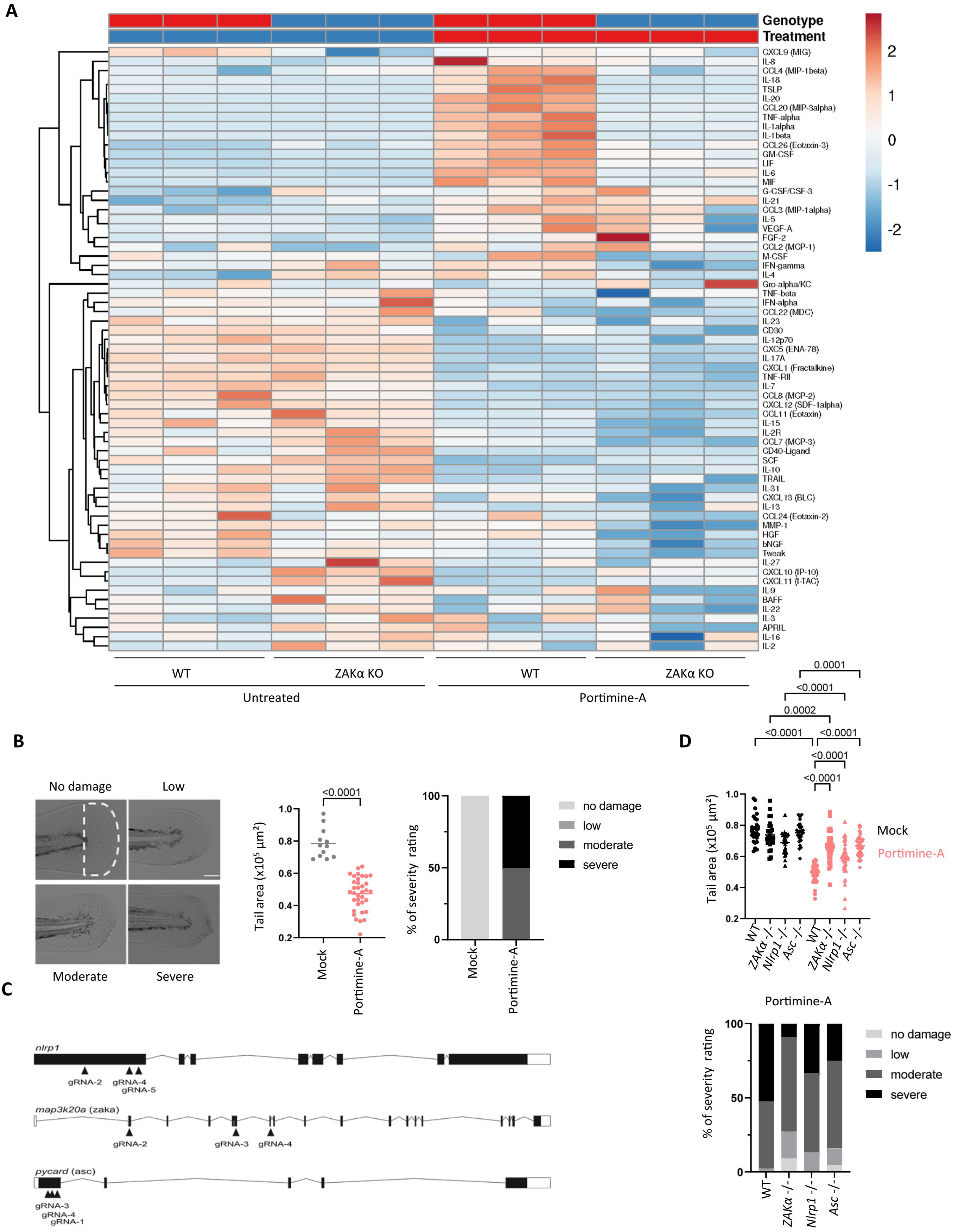
A
Genotype
Treatment
2
1
0
-1
-2
CXCL9 (MIG)
IL-8
CCL4 (MIP-1beta)
IL-18
TSLP
IL-20
CCL20 (MIP-3alpha)
TNF-alpha
IL-1alpha
IL-1beta
CCL26 (Eotaxin-3)
GM-CSF
LIF
IL-6
MIF
G-CSF/CSF-3
IL-21
CCL3 (MIP-1alpha)
IL-5
VEGF-A
FGF-2
CCL2 (MCP-1)
M-CSF
IFN-gamma
IL-4
Gro-alpha/KC
TNF-beta
IFN-alpha
CCL22 (MDC)
IL-23
CD30
IL-12p70
CXC5 (ENA-78)
IL-17A
CXCL1 (Fractalkine)
TNF-RII
IL-7
CCL8 (MCP-2)
CXCL12 (SDF-1alpha)
CCL11 (Eotaxin)
IL-15
IL-2R
CCL7 (MCP-3)
CD40-Ligand
SCF
IL-10
TRAIL
IL-31
CXCL13 (BLC)
IL-13
CCL24 (Eotaxin-2)
MMP-1
HGF
bNGF
Tweak
IL-27
CXCL10 (IP-10)
CXCL11 (I-TAC)
IL-9
BAFF
IL-22
IL-3
APRIL
IL-16
IL-2
WT
ZAKα KO
WT
ZAKα KO
Untreated
Portimine-A
B
No damage
Low
Moderate
Severe
Tail area (x10^5 μm^2)
<0.0001
Mock
Portimine-A
% of severity rating
no damage
low
moderate
severe
C
nlrp1
gRNA-2
gRNA-4
gRNA-5
map3k20a (zaka)
gRNA-2
gRNA-3
gRNA-4
pycard (asc)
gRNA-3
gRNA-4
gRNA-1
D
0.0001
<0.0001
0.0002
<0.0001
<0.0001
<0.0001
<0.0001
Tail area (x10^5 μm^2)
Mock
Portimine-A
WT
ZAKα -/-
Nlrp1 -/-
Asc -/-
WT
ZAKα -/-
Nlrp1 -/-
Asc -/-
Portimine-A
% of severity rating
no damage
low
moderate
severe
WT
ZAKα -/-
Nlrp1 -/-
Asc -/-

**Figure EV4. (refers to Fig. 4): ZAKα and NLRP1 contribution to Portimine-induced skin inflammation in 3D skin and zebrafish models.**

(**A**) 65 cytokine analyzed in 3D skin treated or not with Portimine A. (**B**) Determination and quantification of zebrafish larvae fin tail damage induced by Portimine A (75 nM) in WT larvae (20/group). Two specific parameters were studied for damages quantifications, namely the Tail area (×$10^5$ µm$^2$) and the % of damage severity. Scale bar 100 µm. *P* values indicated in figure, one-way ANOVA (Kruskal–Wallis test). Graphs show one experiment performed three times. (**C**) CRISPR/Cas9 gRNA strategy used to genetically ablate ZAKα, NLRP1 and Asc in zebrafish embryo (for details see material and methods). (**D**) Determination and quantification of zebrafish larvae fin tail damage induced by Portimine A (75 nM) in WT, *Nlrp1-*, *Asc-* and *ZAKa*-deficient larvae (20/group) after 30 h. *P* values indicated in figure, one-way ANOVA (Kruskal–Wallis test). Scale bar 100 µm. Graphs show one experiment performed two times.

 © The Author(s)
